# Frosted DNA: β‐Galactosidase Control of Oligonucleotide Activity

**DOI:** 10.1002/chem.202500347

**Published:** 2025-03-30

**Authors:** Fabian Sinsel, Marius Külp, Michael A. Rieger, Alexander Heckel

**Affiliations:** ^1^ Institute for Organic Chemistry and Chemical Biology Goethe University Frankfurt Max‐von‐Laue‐Str. 7 Frankfurt am Main 60438 Germany; ^2^ Department of Medicine II, Hematology/Oncology Goethe University Frankfurt Frankfurt am Main Germany; ^3^ Cardio‐Pulmonary‐Institute Frankfurt am Main Germany; ^4^ German Cancer Consortium (DKTK) and German Cancer Research Center (DKFZ) Heidelberg Germany; ^5^ Frankfurt Cancer Institute Frankfurt am Main Germany

**Keywords:** click chemistry, cyclic oligonucleotides, enzymatic trigger, oligonucleotide release, β‐galactosidase

## Abstract

We introduce a novel approach for the control of oligonucleotides through enzymatic activation with β‐galactosidase (β‐gal). We use the well‐known enzymatic capability of β‐gal to hydrolyze the β‐galactosidic bond combined with a self‐immolative linker and present three ways of steric or topological blocking of a DNA oligonucleotide. Through a series of *in vitro* experiments with β‐gal variants (recombinant or from human cell lysates), we systematically investigate stability, transitory perturbation, and enzymatic activation. Our approach holds significant promise for applications related to senescence‐associated β‐gal activity, including targeted modulation of gene expression and programmable molecular interventions. The combination of enzymatic activation applied to oligonucleotides represents a significant advance for targeted release in affected cells without the need for external triggering.

## Graphical abstract

β‐Galactosidase is expressed at low level in many native cells and strongly upregulated in senescent cells. In the present study, we use β‐galactosidase activity as internal trigger to control the hybridization of oligonucleotides–eventually selective in senescent cells.

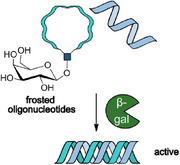

## 1 Introduction

DNA, RNA, and their various analogues are used in a wide range of (therapeutic)^[^ ^1^ ^]^ applications, including the regulation of gene expression (antisense technology^[^ ^2^ ^]^, RNA interference^[^ ^3^ ^]^, CRISPR/Cas9^[^ ^4^ ^]^, riboswitches^[^ ^5^ ^]^, etc.) and the modulation of protein functions (aptamers^[^ ^6^, ^7^ ^]^). For the precise investigation of complex biological processes, such as, for example, the specific targeting of cells, it is often necessary to add another layer of control using triggers: the biological function of an oligonucleotide can be temporarily blocked until a conditional activation occurs. Trigger signals are categorized as external or internal. As an external signal, light is used as a prominent example with great success in spatiotemporal control.^[^ ^8^, ^9^, ^10^ ^]^ For example, persistent reversible photoacids can yield local pH jumps^[^ ^11^ ^]^ or caged aptamers can be used to differentially control protein subdomain activity with precision in time and location.^[^ ^12^ ^]^

An alternative is an abrupt change in temperature.^[^ ^13^ ^]^ However, there are still only very few studies on the control of DNA and RNA activity with internal triggers. We consider a trigger as internal if it comes from the cell itself. Two triggers have been mainly used so far in the context of oligonucleotides: redox potential and enzymatic activity.

The group of Obika‐designed boronated nucleobases enabled H_2_O_2_‐triggered oxidative DNA release.^[^ ^14^ ^]^ Urata et al. synthesized 2′‐*O*‐methyldithiomethyl‐modified RNA, activatable through cleavage by reduction via GSH.^[^ ^15^, ^16^ ^]^ Two more reduction‐sensitive studies dealt with *O* ^6^‐nitrobenzyl‐protected dG, which could be activated via dithionite or the enzyme nitroreductase.^[^ ^17^, ^18^ ^]^

A focus on enzymatic release was put in studies in which linker systems for oligonucleotides were cleaved by nitroreductase^[^ ^19^, ^20^ ^]^, demonstrating an application in zebrafish embryos with morpholino oligonucleotides.^[^ ^21^ ^]^ In another study, thioesterases were used for the activation of siRNA precursors.^[^ ^22^ ^]^ In three very recent studies, caspase, β‐lactamase, and cathepsin B were explored for the regulation of oligonucleotides.^[^ ^23^, ^24^, ^25^ ^]^

β‐Galactosidase (β‐gal)—apart from its use as a prokaryotic reporter gene—is an important biomarker for eukaryotic senescent cells^[^ ^26^, ^27^ ^]^ with upregulation in certain types of cancer.^[^ ^28^ ^]^ Senescent cells play a key role in aging and cancer. They comprise different cell states caused by complex molecular changes (loss of proliferation, apoptosis inhibition, chromatin alterations, metabolic changes). Senescent cells can release a cell type‐specific mixture of bioactive molecules, which results in the senescence‐associated secretory phenotype (SASP). SASP impacts adjacent cells and the extracellular matrix and contributes to age‐related tissue degeneration.^[^ ^29^, ^30^ ^]^ Senescent cells in cancer acquire stem‐cell‐like features and act as cancer‐initiating stem cells, making them resistant to conventional therapies.^[^ ^27^, ^31^ ^]^

β‐gal activity has already been used as an internal trigger: one field of application is β‐gal‐activated fluorescent probes for the visualization of tumor and senescent cells in which very high contrast ratios were already obtained.^[^ ^32^, ^33^ ^]^ A few other studies dealt with β‐gal‐activity‐dependent drug release^[^ ^34^, ^35^, ^36^, ^37^, ^38^ ^]^, and another one enabled photoactivation only after releasing galactose.^[^ ^39^ ^]^

For this study, with our previous experience in the regulation of DNA and RNA with light, we wanted to explore whether the same strategies as for light regulation can be applied to the regulation with β‐gal activity. Masking the activity of a functional compound with a photolabile group is referred to as “(photo)caging,” the photorelease is called “uncaging.” In analogy, we propose the term “frosting” when a β‐gal or any other glycosidase trigger is installed in a functional molecule and “defrosting” when the activity is released enzymatically.

Our previous contributions to photocontrol of DNA/RNA had followed three strategies: A very robust way for a local masking of DNA/RNA is to attach photolabile groups to the nucleobases or the phosphodiester backbone. While the purpose of the former is a local perturbation of duplex structure due to inhibition of Watson–Crick–Franklin base pairing,^[^ ^40^ ^]^ the purpose of the latter is to interfere with the binding of (protein) ligands to DNA duplexes.^[^ ^41^ ^]^ Both modifications have a local effect of 1–2 base pairs on either side.^[^ ^40^ ^]^ While phosphate photocaging was synthetically easier to achieve, strategically, it plays only a minor role so far as much better regulation efficiencies are typically obtained with a local perturbation of duplex structure via nucleobase‐photocaging. This strategy is ideal in situations where perfect local base pairing is required for a biological function (such as, for example, in a promoter region^[^ ^42^ ^]^ or in the middle of an siRNA^[^ ^43^ ^]^). Repetitive use of nucleobase modifications is not prohibitive, as we have shown controlling miRNA function with light.^[^ ^44^, ^45^ ^]^

For a very efficient global control of base pairing, we had proposed a cyclization approach.^[^ ^46^, ^47^ ^]^ A DNA duplex is rather stiff, whereas a single strand is rather flexible. We had shown that, for example, a 32‐mer DNA single strand cyclized via a photocleavable linker (phototether) was very efficiently blocked from interacting with its counter strand. Even the function of a 90mer aptamer could be completely controlled by bicircularization and reactivation with light. Thus, temporary cyclization acts via a global distortion of an active conformation.

**Figure 1:**
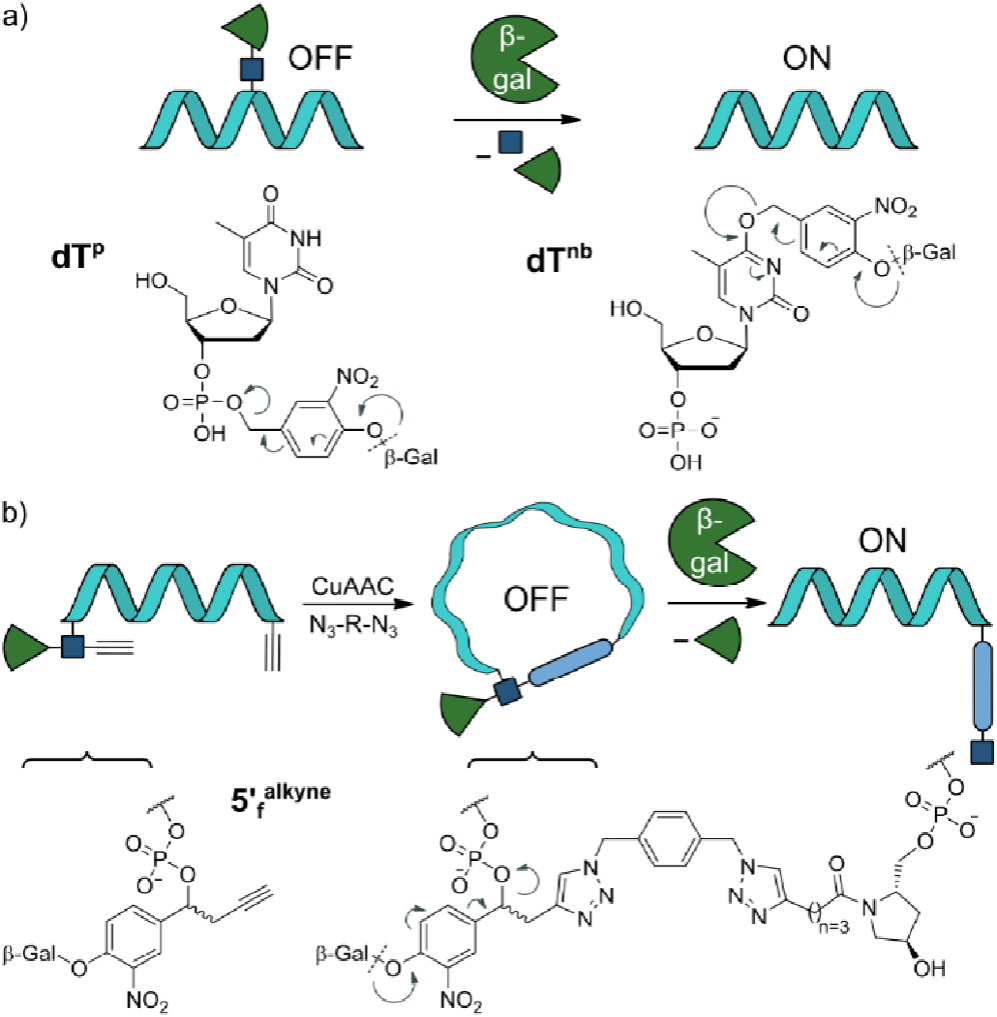
a) The phosphate‐“frosted” nucleotide **dT^p^** and the nucleobase‐“frosted” nucleotide **dT^nb^** used in this study. “Frosted” oligonucleotides are either protein‐binding‐(**dT^p^**) or base‐pairing‐impaired (**dT^nb^**). Endogenous β‐galactosidase (β‐gal), overexpressed in senescent cells or cancer cells can release the active oligonucleotide. Upon enzymatic cleavage of galactose, a self‐immolative linker is eliminated, restoring protein interaction or Watson–Crick–Franklin base‐pairing activity. (β‐gal, β‐galactosidase; β‐Gal, β‐galactose). b) Alternatively, duplex formation can be very efficiently inhibited by temporary cyclization. β‐gal opens the cycle, and the linearized oligonucleotide is available for duplex formation.

Figure 1 shows schematically how we transferred these three design principles to nucleobase‐ or phosphate‐frosted oligonucleotides or cyclized ones. For compatibility with the enzyme (β‐gal), we installed a self‐immolative linker between the two entities, which had been used in the context of β‐gal activation before.^[^ ^48^ ^]^

## 2 Results and Discussion

**Scheme 1:**
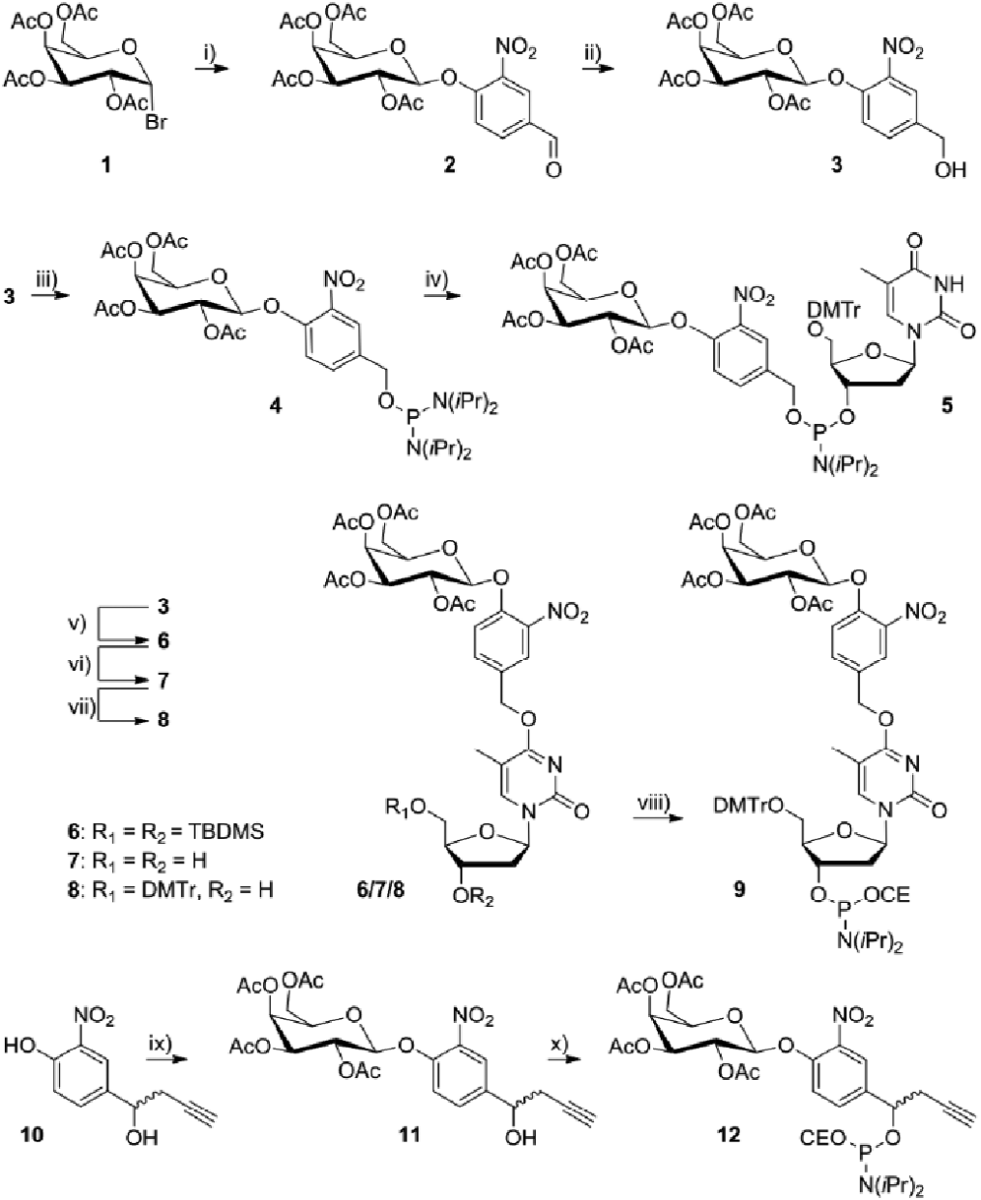
Synthesis of phosphoramidites **5** (**dT^p^**), **9** (**dT^nb^**) and **12** (**5′_f_^alkyne^**) from precursors **3** and **11**: i) 4‐hydroxy‐2‐nitrobenzaldehyde, Ag_2_O, 95%; ii) NaBH_4_, 98%; iii) P(N(*i*Pr)_2_)_2_Cl, N(Et)_3_, 23%; iv) 5′‐DMTr dT, 4,5‐dicyanoimidazole, 41%; v) triazole‐activated 3′‐, 5′‐bis‐TBDMS dT, DBU, 73%; vi) TBAF, AcOH, 79%; vii) DMTrCl, 76%; viii) PN(*i*Pr)_2_(OCE)Cl, NEt(*i*Pr)_2_, 61%; ix) **1**, Ag_2_CO_3_, HMTETA, 88%; x) PN(*i*Pr)_2_(OCE)Cl, NEt(*i*Pr)_2_, 38%.

The synthesis route (Scheme 1) for the phosphate‐ or nucleobase‐frosted dT phosphoramidites started with a Königs–Knorr reaction of the protected glycosyl donor **1** to obtain galactoside **2**. Due to the neighboring group effect of the 2‐*O*‐acetyl substituent, the β‐galactoside is selectively formed.

Reduction with NaBH_4_ afforded the alcohol **3**. For a phosphate‐frosted dT‐derivative, compound **3** was phosphitylated (→**4**) and then reacted with a suitably protected dT derivative under microwave assistance (→**5**). To obtain the nucleobase‐frosted phosphoramidite **9**, compound **3** was installed on another suitably protected dT derivative (→**6**). Desilylation (→**7**), tritylation (→**8**), and phosphitylation yielded building block **9**. To further investigate the cyclization approach, we obtained the 5′‐modifier **12** after galactosylation of precursor **10** (→**11**) and phosphitylation.

**Figure 2:**
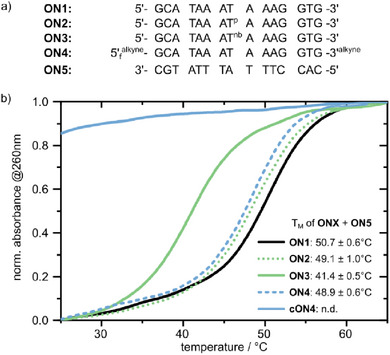
a) Oligonucleotides tested in this study. b) Duplex melting temperature curves of the native (**ON1**), phosphate‐frosted (**ON2**), nucleobase‐frosted (**ON3**), cyclization‐ready (**ON4**), and cyclized (**cON4**) sense‐strand with their complementary DNA‐strand (1x PBS, 1 µM, *n* = 7).

At first, phosphoramidites **5** and **9** were used for the solid‐phase oligonucleotide synthesis. We chose a 15‐mer benchmark DNA sequence, which had been used previously to assess the effects of photocaging (Figure 2 top).^[^ ^49^ ^]^ Oligonucleotide **ON1** was the unmodified control sequence, whereas **ON2** and **ON3** contained a phosphate‐frosted **dT^p^** or a nucleobase‐frosted **dT^nb^**, respectively. To test the cyclization approach, we used the same sequence with a 3′‐alkyne‐modified hydroxyprolinol and with phosphoramidite **12** as 5′‐modifier (→**ON4**). The galactose residues were deprotected along with the other nucleobase protecting groups required for solid‐phase synthesis during the basic post‐synthesis treatment with potassium carbonate. The linear **ON4** was then cyclized to yield **cON4** using bis(azidomethyl)benzene in two Cu(I)‐catalyzed cycloadditions. **ON5** was the unmodified counter strand for all other strands.

As shown before with photocaged and phototethered oligonucleotides, we tested our frosted derivatives by performing thermal melting studies (Figure 2). The benchmark sequence (**ON1 **+ **ON5**) had a melting temperature of 50.7°C. Upon introduction of a single **dT^p^** residue (**ON2**), this was only slightly reduced to 49.1°C. In comparison, with phosphate photocages, similar melting temperature reductions had been obtained.^[^ ^50^ ^]^ This is not unexpected, as such phosphate modifications are not designed to interfere with base‐pairing but rather afford a local sterical blocking of interaction with, e.g., proteins. A single **dT^nb^** residue, however, afforded a reduction of 9.3°C in melting temperature—clearly showing the local destabilization of duplex formation. The magnitude of the effect is in line with results obtained from optimized photocages (ca. 10°C).^[^ ^49^ ^]^ For **ON4** (which had only terminal modifications), before cyclization, we obtained a melting temperature similar to the phosphate‐frosted **ON2**. Cyclization showed the same strong effect as we had obtained before with phototethering: no interaction was detected by melting temperature studies. This goes in line with native gel electrophoresis studies proving the superiority of the cyclization approach also for frosting (Supporting Information).

**Figure 3:**
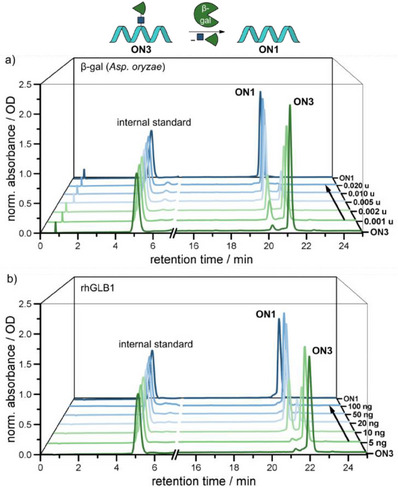
RP‐HPLC chromatograms (254 nm) of “defrosting” assays with nucleobase‐frosted **ON3**. a) β‐gal (*Aspergillus oryzae*), citrate‐phosphate‐buffer (pH 4.5), 37°C, 30 min. b) *rhGLB1*, citrate‐phosphate‐buffer (pH 3.5), 37°C, 30 min.

Next, we investigated the “defrosting” reaction. Therefore, we exposed the nucleobase‐frosted **ON3** to increasing amounts of β‐gal—at first isolated from *Aspergillus oryzae* (Figure 3a). This variant of β‐gal had also been used in previous studies for the release of galactose‐protected NIR‐emitting fluorescent probes. It is structurally more similar to the human β‐gal than the *E*. coli β‐gal, which is used in other studies.^[^ ^51^ ^]^ We observed a clean transition of 100 pmol of the nucleobase‐frosted **ON3** to the unmodified **ON1** in less than 30 min. We performed the same experiment with recombinant human β‐gal (rhGLB1) and observed similar results (Figure 3b). In the absence of β‐gal, **ON3** was stable in PBS for at least 4 weeks without any signs of defrosting (Supporting Information). Interestingly, the phosphate‐frosted **ON2** was less stable, as we observed 38% decomposition in PBS at room temperature after 1 week. As phosphate modification has been strategically less relevant in the field of photocaging, we did not further investigate this finding in the current study. This approach might need different linkers—which will be the subject of another study.

**Figure 4:**
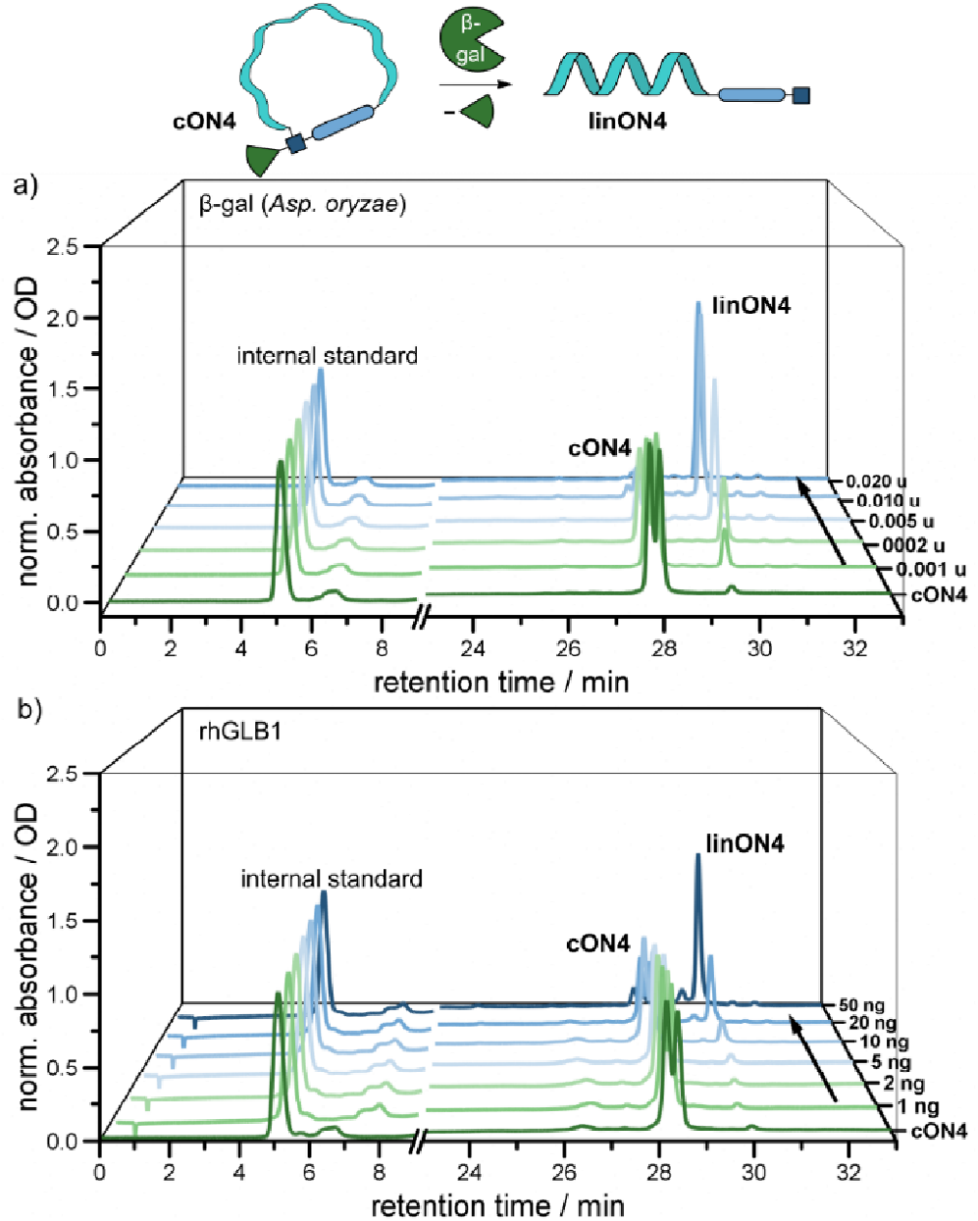
RP‐HPLC chromatograms (254 nm) of “defrosting” assays with cyclized 15mer **cON4**. a) β‐gal (*Aspergillus oryzae*), citrate‐phosphate‐buffer (pH 4.5), 37 °C, 30 min. b) *rhGLB1*, citrate‐phosphate‐buffer (pH 3.5), 37°C, 30 min.

On the other hand, the cyclic frosted **cON4** showed once more very promising results (Figure 4). Due to the extra stereogenic center in the 5′‐modifier, for which we did not see any requirement for a stereospecific synthesis or separation, the HPLC traces show two peaks for the starting material. Both could be converted without any apparent difference in reaction rate to the linearized **linON4** with β‐gal from *Aspergillus oryzae* as well as with recombinant human β‐gal. Importantly, **cON4** was stable in PBS buffer without any signs of defrosting for 4 weeks at room temperature.

**Figure 5:**
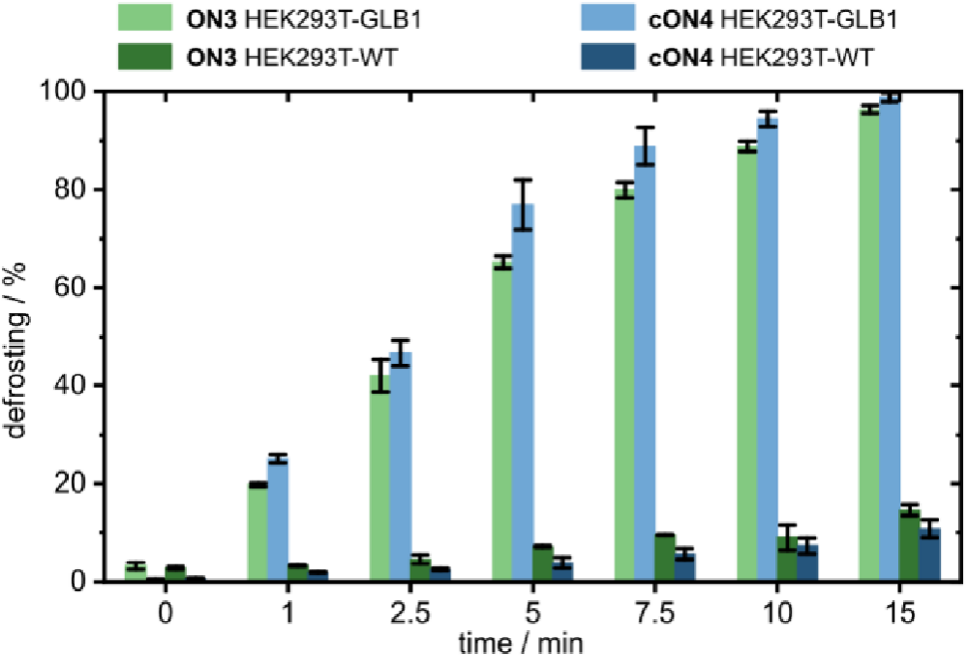
Results of exposure of **ON3** and **cON4** to cell lysate of native HEK293T cells or cell lysate from HEK293T cells overexpressing human β‐gal. (triplicates, error bars: standard deviation).

While stabilities in PBS buffer are important, it remains to be tested how stable the frosted constructs are in a cell. We established a cellular senescence surrogate system by overexpressing the open reading frame of the β‐gal‐encoding human gene GLB1 in HEK293T cells. Expression of the β‐gal protein was validated using capillary electrophoresis followed by antibody detection (WES Simple Western system). The enzymatic activity of β‐gal in cell lysates was quantified using a fluorescence detection assay, in which β‐gal hydrolyzes a non‐fluorescent substrate into a fluorescent product. The enzymatic assay confirmed a 3–5 times higher β‐gal activity in overexpressing cells (HEK293T‐GLB1) compared to wild‐type cells (HEK293T‐WT). We exposed **ON3** and **cON4** to lysates of both HEK293T‐GLB1 and HEK293T‐WT (Figure 5). While both **ON3** and **cON4** were defrosted to 50% within 3 min with lysate from HEK293T‐GLB1, 50% defrosting was not even reached with WT lysate after 1 h. These results demonstrate efficient defrosting of oligonucleotides using expressed human β‐gal in human cells with similar contrast ratios being observed with frosted fluorophores before.^[^ ^32^, ^33^ ^]^

## 3 Conclusion

En route to precision functional molecules, extra levels of control of activity can be of great importance. Such trigger signals can be both external (for example, light) or internal (for example, local environmental conditions or local enzymatic activity). While the former appears to be appealingly general, there are some situations where irradiation is not possible (light penetration depth or, in the case of non‐solid tumors). With the present study, we contribute to the latter principle by investigating the control of oligonucleotide activity by β‐gal, which is expressed at low levels in many native cells and strongly upregulated in senescent cells—as proven by previous studies with β‐gal‐activated fluorescent probes.^[^ ^32^, ^33^ ^]^

In parallel to our study, the Seio group showed a similar approach.^[^ ^52^ ^]^ In their study, they also synthesized what we call **dT^nb^** in our study. They also performed melting temperature studies and found very comparable results in a different sequence context. They investigated the effect of multiple galactose‐modified residues in their study. While they went on to study the kinetics of defrosting using different linkers between the β‐Gal residue and the dT and used β‐gal from *E. coli*, we studied different architectures of frosting and used β‐gal from *Aspergillus oryzae* and human β‐gal. Phosphodiester‐frosting (with the linker we chose for this study) was less successful, as the resulting **ON2** showed signs of decomposition at room temperature in PBS. For the nucleobase‐frosted **ON3** and the cyclic frosted **cON4**, melting temperature assays showed the expected local or global effects of destabilization of duplex formation as we have seen with light‐activatable analogs. In the latter domain, we had previously established a very high correlation between the results of such melting temperature studies and the applicability in various complex biological application scenarios. Both the nucleobase and the cyclic frosted **ON3** and **cON4**, respectively, showed very high stability in PBS and prompt defrosting. Importantly, we obtained a very good contrast probing stability/defrosting in cell lysate of native HEK293T cells or HEK293T cells with ectopic expression of human β‐gal. We believe that this paves the way for future control of oligonucleotide activity with different external or internal triggers—or eventually a combination of both.

## Conflicts of Interests

The authors declare no conflicts of interest.

## Supporting information



Supporting Information

## Data Availability

The authors have cited additional references within the Supporting Information.^[^ ^53^ ^]^ The data that support the findings of this study are available in the supporting information of this article That is available at: https://doi.org/10.1002/chem.202500347

## References

[1] A. Khvorova , J. K. Watts , Nat. Biotechnol. 2017, 35, 238. 28244990 10.1038/nbt.3765 PMC5517098

[2] T. C. Roberts , R. Langer , M. J. A. Wood , Nat. Rev. Drug Discovery 2020, 19, 673. 32782413 10.1038/s41573-020-0075-7 PMC7419031

[3] R. L. Setten , J. J. Rossi , S. Han , Nat. Rev. Drug Discovery 2019, 18, 421. 30846871 10.1038/s41573-019-0017-4

[4] J. D. Sander , J. K. Joung , Nat. Biotechnol. 2014, 32, 347. 24584096 10.1038/nbt.2842 PMC4022601

[5] K. Kavita , R. R. Breaker , Trends Biochem. Sci. 2023, 48, 119. 36150954 10.1016/j.tibs.2022.08.009 PMC10043782

[6] M. Famulok , J. S. Hartig , G. Mayer , Chem. Rev. 2007, 107, 3715. 17715981 10.1021/cr0306743

[7] A. D. Keefe , S. Pai , A. Ellington , Nat. Rev. Drug Discovery 2010, 9, 537. 20592747 10.1038/nrd3141 PMC7097324

[8] P. Klán , T. Šolomek , C. G. Bochet , A. Blanc , R. Givens , M. Rubina , V. Popik , A. Kostikov , J. Wirz , Chem. Rev. 2013, 113, 119. 23256727 10.1021/cr300177k PMC3557858

[9] R. Weinstain , T. Slanina , D. Kand , P. Klán , Chem. Rev. 2020, 120, 13135. 33125209 10.1021/acs.chemrev.0c00663 PMC7833475

[10] M. J. Hansen , W. A. Velema , M. M. Lerch , W. Szymanski , B. L. Feringa , Chem. Soc. Rev. 2015, 44, 3358. 25917924 10.1039/c5cs00118h

[11] T. Halbritter , C. Kaiser , J. Wachtveitl , A. Heckel , J. Org. Chem. 2017, 82, 8040. 28686024 10.1021/acs.joc.7b01268

[12] G. Mayer , J. Müller , T. Mack , D. F. Freitag , T. Höver , B. Pötzsch , A. Heckel , ChemBioChem 2009, 10, 654. 19189364 10.1002/cbic.200800814

[13] S. D. Knutson , A. A. Sanford , C. S. Swenson , M. M. Korn , B. A. Manuel , J. M. Heemstra , J. Am. Chem. Soc. 2020, 142, 17766. 33017148 10.1021/jacs.0c08996

[14] S. Mori , K. Morihiro , T. Okuda , Y. Kasahara , S. Obika , Chem. Sci. 2018, 9, 1112. 29629168 10.1039/c7sc04318j PMC5875086

[15] Y. Ochi , O. Nakagawa , K. Sakaguchi , S. Wada , H. Urata , Chem. Commun. 2013, 49, 7620. 10.1039/c3cc43725f 23872984

[16] Y. Ochi , M. Imai , O. Nakagawa , J. Hayashi , S. Wada , H. Urata , Bioorg. Med. Chem. Lett. 2016, 26, 845. 26755395 10.1016/j.bmcl.2015.12.074

[17] M. Ikeda , M. Kamimura , Y. Hayakawa , A. Shibata , Y. Kitade , ChemBioChem 2016, 17, 1304. 27124306 10.1002/cbic.201600164

[18] Y. Hayakawa , A. Banno , H. Kitagawa , S. Higashi , Y. Kitade , A. Shibata , M. Ikeda , ACS Omega 2018, 3, 9267. 31459058 10.1021/acsomega.8b01177 PMC6645092

[19] H. Saneyoshi , Y. Yamamoto , K. Kondo , Y. Hiyoshi , A. Ono , J. Org. Chem. 2017, 82, 1796. 28112510 10.1021/acs.joc.6b02527

[20] H. Saneyoshi , K. Iketani , K. Kondo , T. Saneyoshi , I. Okamoto , A. Ono , Bioconjugate Chem. 2016, 27, 2149. 10.1021/acs.bioconjchem.6b00368 27598574

[21] S. Yamazoe , L. E. McQuade , J. K. Chen , ACS Chem. Biol. 2014, 9, 1985. 25069083 10.1021/cb500429u PMC4168795

[22] B. R. Meade , K. Gogoi , A. S. Hamil , C. Palm‐Apergi , A. van den Berg , J. C. Hagopian , A. D. Springer , A. Eguchi , A. D. Kacsinta , C. F. Dowdy , A. Presente , P. Lönn , M. Kaulich , N. Yoshioka , E. Gros , X.‐S. Cui , S. F. Dowdy , Nat. Biotechnol. 2014, 32, 1256. 25402614 10.1038/nbt.3078 PMC4378643

[23] L. Yang , J. H. Eberwine , I. J. Dmochowski , Bioconjugate Chem. 2020, 31, 2172. 10.1021/acs.bioconjchem.0c00362 PMC8319955 32786369

[24] K. E. Darrah , S. Albright , R. Kumbhare , M. Tsang , J. K. Chen , A. Deiters , ACS Chem. Biol. 2023, 18, 2176. 37326511 10.1021/acschembio.3c00027 PMC10592181

[25] Z. Wang , X. Fan , G. Mu , X. Zhao , Q. Wang , J. Wang , X. Tang , Mol. Ther.–Nucleic Acids 2023, 33, 548. 37588686 10.1016/j.omtn.2023.07.022 PMC10425675

[26] G. P. Dimri , X. Lee , G. Basile , M. Acosta , G. Scott , C. Roskelley , E. E. Medrano , M. Linskens , I. Rubelj , O. Pereira‐Smith , Proc. Natl. Acad. Sci. U. S. A. 1995, 92, 9363. 7568133 10.1073/pnas.92.20.9363 PMC40985

[27] S. Lee , C. A. Schmitt , Nat. Cell Biol. 2019, 21, 94. 30602768 10.1038/s41556-018-0249-2

[28] S. K. Chatterjee , M. Bhattacharya , J. J. Barlow , Cancer Res. 1979, 39, 1943. 445394

[29] M. Xu , T. Pirtskhalava , J. N. Farr , B. M. Weigand , A. K. Palmer , M. M. Weivoda , C. L. Inman , M. B. Ogrodnik , C. M. Hachfeld , D. G. Fraser , J. L. Onken , K. O. Johnson , G. C. Verzosa , L. G. P. Langhi , M. Weigl , N. Giorgadze , N. K. LeBrasseur , J. D. Miller , D. Jurk , R. J. Singh , D. B. Allison , K. Ejima , G. B. Hubbard , Y. Ikeno , H. Cubro , V. D. Garovic , X. Hou , S. J. Weroha , P. D. Robbins , L. J. Niedernhofer , S. Khosla , T. Tchkonia , J. L. Kirkland , Nat. Med. 2018, 24, 1246. 29988130 10.1038/s41591-018-0092-9 PMC6082705

[30] A. Calcinotto , J. Kohli , E. Zagato , L. Pellegrini , M. Demaria , A. Alimonti , Physiol. Rev. 2019, 99, 1047. 30648461 10.1152/physrev.00020.2018

[31] M. Demaria , M. N. O'Leary , J. Chang , L. Shao , S. Liu , F. Alimirah , K. Koenig , C. Le , N. Mitin , A. M. Deal , S. Alston , E. C. Academia , S. Kilmarx , A. Valdovinos , B. Wang , A. de Bruin , B. K. Kennedy , S. Melov , D. Zhou , N. E. Sharpless , H. Muss , J. Campisi , Cancer Discovery 2017, 7, 165. 27979832 10.1158/2159-8290.CD-16-0241 PMC5296251

[32] Y. Yao , Y. Zhang , C. Yan , W.‐H. Zhu , Z. Guo , Chem. Sci. 2021, 12, 9885. 34349961 10.1039/d1sc02069b PMC8317648

[33] D. Asanuma , M. Sakabe , M. Kamiya , K. Yamamoto , J. Hiratake , M. Ogawa , N. Kosaka , P. L. Choyke , T. Nagano , H. Kobayashi , Y. Urano , Nat. Commun. 2015, 6, 6463. 25765713 10.1038/ncomms7463 PMC4382686

[34] M. Thomas , J. Clarhaut , P.‐O. Strale , I. Tranoy‐Opalinski , J. Roche , S. Papot , ChemMedChem 2011, 6, 1006. 21442760 10.1002/cmdc.201100114

[35] T. Legigan , J. Clarhaut , I. Tranoy‐Opalinski , A. Monvoisin , B. Renoux , M. Thomas , A. Le Pape , S. Lerondel , S. Papot , Angew. Chem., Int. Ed. 2012, 51, 11606. 10.1002/anie.201204935 22996951

[36] M. Maiti , K. Kikuchi , K. K. Athul , A. Kaur , S. Bhuniya , Chem. Commun. 2022, 58, 6413. 10.1039/d2cc01825j 35543438

[37] Y. Song , X. Li , D. Shi , T. Sun , W. Liu , X. Li , S. Qiao , X. Chen , Y. Guo , J. Li , Chem. Sci. 2022, 13, 11738. 36320912 10.1039/d2sc03525a PMC9580481

[38] J. Xiong , J. C. H. Chu , W.‐P. Fong , C. T. T. Wong , D. K. P. Ng , J. Am. Chem. Soc. 2022, 144, 10647. 35639988 10.1021/jacs.2c04017

[39] A. Z. Suzuki , T. Sakano , H. Sasaki , R. Watahiki , M. Sone , K. Horikawa , T. Furuta , Chem. Commun. 2021, 57, 5630. 10.1039/d1cc01405f 34018507

[40] P. Seyfried , M. Heinz , G. Pintér , D.‐P. Klötzner , Y. Becker , M. Bolte , H. R. A. Jonker , L. S. Stelzl , G. Hummer , H. Schwalbe , A. Heckel , Chem. ‐ Eur. J. 2018, 24, 17568. 30199112 10.1002/chem.201804040

[41] W. T. Monroe , M. M. McQuain , M. S. Chang , J. S. Alexander , F. R. Haselton , J. Biol. Chem. 1999, 274, 20895. 10409633 10.1074/jbc.274.30.20895

[42] L. Kröck , A. Heckel , Angew. Chem., Int. Ed. 2005, 44, 471. 10.1002/anie.200461779 15624132

[43] V. Mikat , A. Heckel , RNA 2007, 13, 2341. 17951332 10.1261/rna.753407 PMC2080613

[44] T. Lucas , F. Schäfer , P. Müller , S. A. Eming , A. Heckel , S. Dimmeler , Nat. Commun. 2017, 8, 15162. 28462946 10.1038/ncomms15162 PMC5418571

[45] F. Schäfer , J. Wagner , A. Knau , S. Dimmeler , A. Heckel , Angew. Chem., Int. Ed. 2013, 52, 13558. 10.1002/anie.201307502 24174377

[46] P. Seyfried , L. Eiden , N. Grebenovsky , G. Mayer , A. Heckel , Angew. Chem., Int. Ed. 2017, 56, 359. 10.1002/anie.201610025 27897376

[47] P. Müller , P. Seyfried , A. Frühauf , A. Heckel , in Methods in Enzymology (Ed.: A. Deiters ), Academic Press, Cambridge, Massachusetts 2019, pp. 89–111. 10.1016/bs.mie.2019.04.019 31370937

[48] A. K. Ghosh , S. Khan , F. Marini , J. A. Nelson , D. Farquhar , Tetrahedron Lett. 2000, 41, 4871.

[49] A. Rodrigues‐Correia , M. B. Koeppel , F. Schäfer , K. B. Joshi , T. Mack , A. Heckel , Anal. Bioanal. Chem. 2011, 399, 441. 20953770 10.1007/s00216-010-4274-7

[50] J. Kaufmann , F. Sinsel , A. Heckel , Chem. ‐ Eur. J. 2023, 29, e202204014. 36562762 10.1002/chem.202204014

[51] X. Li , W. Qiu , J. Li , X. Chen , Y. Hu , Y. Gao , D. Shi , X. Li , H. Lin , Z. Hu , G. Dong , C. Sheng , B. Jiang , C. Xia , C.‐Y. Kim , Y. Guo , J. Li , Chem. Sci. 2020, 11, 7292. 34123013 10.1039/d0sc01234c PMC8159415

[52] K. Miyaji , Y. Masaki , K. Seio , Bioconjugate Chem. 2024, 35, 1587. 10.1021/acs.bioconjchem.4c00376 39376088

[53] S. Wingert , F. B. Thalheimer , N. Haetscher , M. Rehage , T. Schroeder , M. A. Rieger , Stem Cells 2016, 34, 699. 26731607 10.1002/stem.2282 PMC4832267

