# Supplementary material for: Frosted DNA: β‐Galactosidase Control of Oligonucleotide Activity
Source: Chemistry. 2025 Mar 30;31(24):e202500347. doi: 10.1002/chem.202500347 (PMC12043040; doi:10.1002/chem.202500347)
Supplement: Supplementary file 1 — Supporting Information [file CHEM-31-e202500347-s001.pdf]

Chemistry – A European Journal

Supporting Information

©Wiley-VCH 2021

69451 Weinheim, Germany

Frosted DNA: β-Galactosidase Control of Oligonucleotide Activity

Fabian Sinsel,[a] Marius Külp,[b,c,d] Michael Rieger,[b,c,d,e] and Alexander Heckel*[a]

[a] F. Sinsel, Prof. Dr. A. Heckel
Institute for Organic Chemistry and Chemical Biology
Goethe University Frankfurt
Max-von-Laue-Str. 7, 60438 Frankfurt am Main
E-mail: heckel@uni-frankfurt.de

[b] Dr. M. Külp, Prof. Dr. M. Rieger
Department of Medicine II, Hematology/Oncology
Goethe University Frankfurt
Frankfurt am Main, Germany

[c] Dr. M. Külp, Prof. Dr. M. Rieger
Cardio-Pulmonary-Institute
Frankfurt am Main, Germany

[d] Dr. M. Külp, Prof. Dr. M. Rieger
German Cancer Consortium (DKTK) and German Cancer Research Center (DKFZ)
Heidelberg, Germany

[e] Prof. Dr. M. Rieger
Frankfurt Cancer Institute
Frankfurt am Main, Germany
E-mail: m.rieger@em.uni-frankfurt.de

DOI: 10.1002/anie.2021XXXXX

Table of Contents

1. Chemical Synthesis 1
    - Materials and Methods 1
    - Precursor Synthesis 3
    - Phosphate Frosting 5
    - Nucleobase Frosting 7
2. Alkyne-Modified 5’-Frosting 12
3. Oligonucleotide Synthesis 15
    - Materials and Methods 15
    - Purification 16
    - Individual purification of ON2, ON3 and ON4 17
4. CuAAC Oligonucleotide Cyclization 19
5. Duplex Melting Temperature Measurement 22
6. Polyacrylamide Gel Electrophoresis (PAGE, native) 23
7. Oligonucleotide Stability Tests 24
8. β-Galactosidase 25
9. β-Gal Overexpressing HEK293T Cell Lysate 28
    - Cell Culture 28
    - Establishment of a β-gal Overexpressing Cellular System 28
    - Enzymatic Assay 28
    - Oligonucleotide Assay 30
10. NMR Spectra 31
11. Mass Spectra (Small Molecules) 46
12. Mass Spectra (Oligonucleotides) 54
13. References 57

1. Chemical Synthesis

Materials and Methods

Synthetic procedures and material

Reactions involving dry solvents or moisture sensitive chemicals were carried out in a protective argon atmosphere. All reagents and solvents were purchased from commercial sources and used without further purification. Dry solvents were purchased via molecular sieves from *Acros Organics*.

Reactions were monitored using silica gel 60-coated TLC sheets (*ALUGRAM Xtra SIL G/UV*, 0.20 mm silica gel 60, UV_{254} indicator, *Macherey-Nagel*) and silica gel 60 (0.04 - 0.063 mm, *Macherey-Nagel*) was used for purification by silica gel columns. For the purification of phosphoramidites, the silica gel column was washed with the corresponding eluent containing 1.5% triethylamine before the crude product was applied.

Small molecule mass spectra

Mass spectra by electrospray ionization (ESI) were obtained using a *Thermo Fisher Surveyor MSQ device*. The measurements were carried out autonomously or by *MS Service Riedberg* (Goethe University).

High-resolution mass spectrometry (HRMS) was performed using a *LTQ Orbitrap XL* (MALDI-HRMS) by *Thermo Fisher* and a *MicroTof-qII* (ESI-HRMS) from *Bruker*. The measurements were executed by *MS Service Riedberg* (Goethe University).

NMR spectra

NMR spectra were recorded in chloroform-d_1 ($CDCl_3$-d_1) from *Deutero* or dimethyl sulfoxide-d_6 (DMSO-d_6) from *euroisotop* using spectrometers from *Bruker:*

Avance AV400	^{1}H: 400.1 MHz	^{13}C: 100.6 MHz	^{31}P: 162.0 MHz
Avance III HD AV500 MHz	^{1}H: 500.2 MHz	^{13}C: 125.8 MHz	^{31}P: 202.5 MHz

The measurements were obtained at room temperature. 2D NMR spectra were also recorded on the AV400 and AV500 for a clear assignment, these included ^{1}H-^{1}H-COSY, ^{1}H-^{13}C-HSQC and ^{1}H-^{13}C-HMBC spectra. All shifts are reported in ppm using the solvent signal as an internal reference:

$CDCl_3$-d_1	^{1}H: 7.26 ppm (s)	^{13}C: 77.16 ppm (t)
DMSO-d_6	^{1}H: 2.50 ppm (quint)	^{13}C: 39.52 ppm (sept)

The deuterated solvents were kept under an argon atmosphere.

The following abbreviations were used to describe the multiplicities: s = singlet, d = doublet, dd = doublet of doublets, t = triplet, q = quartet, m = multiplet, (b) = broad signal. Coupling constants are reported in Hertz (Hz). The signals of the ^{1}H- and ^{13}C-NMR spectra were assigned to their positions in the respective molecules and assigned as clearly as possible. Figure S1 shows the assignment of the most important positions for ^{1}H-NMR spectra, all other positions of the core structure can be determined from this.

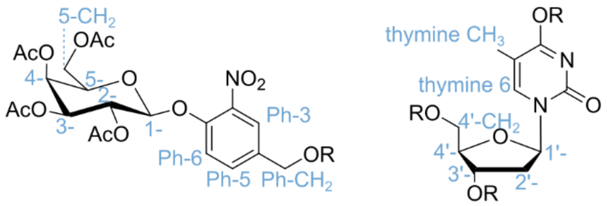

Figure S1: Assignment of the most relevant positions for NMR spectroscopy for the core-structures of β-Gal combined with the self-immolative linker and modified 2'-deoxythymidine.

The remaining signals of the protection groups or functional groups are identified as clearly as possible by summarizing individual groups and marking them in *italics*: OAc means the CH_3 of -O-COOCH_3 (^{1}H), OAc-C_{sp2} represents –O-*C*OOCH$_3$ (^{13}C) and OAc-*C*H$_3$ is for -O-*C*OO*C*H$_3$ (^{13}C).

The compounds **5**, **9**, **10**, **11** and **12** were obtained as stereoisomers, their corresponding NMR spectra showed partially split signals. These were assigned to the respective positions and, if possible, multiplicities and coupling constants were specified. The following is an example from the ^{1}H-NMR spectrum of **5**: 7.82 + 7.73 (2x d, $^4J_{H\text{-}H}$ = 2.0 Hz, Σ = 1H, Ph-H3) – which means, that the signal for Ph-H3 is split into two doublets at 7.82 and 7.73 ppm with a respective coupling constant of 2.0 Hz and an overall integration of “1”. If no clear statement could be made, the signals were marked as multiplets.

Precursor Synthesis

Synthesis of (2*R*,3*S*,4*S*,5*R*,6*S*)-2-(acetoxymethyl)-6-(4-formyl-2-nitrophenoxy)tetrahydro-2*H*-pyran-3,4,5-triyl triacetate (2)

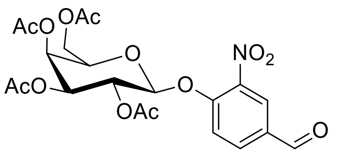

2
$C_{21}H_{23}NO_{13}$
497.41 g mol^{-1}

2,3,4,6-Tetra-*O*-acetyl-α-D-galactopyranosyl bromide **1** (10.0 g, 24.3 mmol, 1.00 eq) was dissolved in 500 mL dry acetonitrile and silver(I) oxide (22.54 g, 97.3 mmol, 4.00 eq) was added. The black suspension was stirred for 10 minutes at room temperature before 4-hydroxy-3-nitrobenzaldehyde (6.91 g, 41.3 mmol, 1.70 eq) was added and a greenish color occurred. After stirring for 20 h at room temperature the suspension was filtered through celite and washed with ethyl acetate until the filtrate was clear. The solvent was removed under reduced pressure and the residue was dissolved in 500 mL ethyl acetate. The organic layer was washed six times with 100 mL conc. $NaHCO_3$-solution and three times with 100 mL conc. NaCl-solution and dried over Na_2SO_4. The solvent was removed and pure **2** was obtained as a yellow solid.

Yield: 11.49 g (23.10 mmol, 95%).

TLC: R_f = 0.41 (cyclohexane/ethyl acetate 1:1).

^{1}H-NMR: (400 MHz, $CDCl_3$-d_1): δ (ppm) = 9.98 (s, 1H, CHO), 8.30 (d, $^4J_{H-H}$ = 2.0 Hz, 1H, Ph-H3), 8.06 (dd, $^3J_{H-H}$ = 8.6 Hz, $^4J_{H-H}$ = 2.0 Hz, 1H, Ph-H5), 7.48 (d, $^3J_{H-H}$ = 8.6 Hz, 1H, Ph-H6), 5.58 (dd, $^3J_{H-H}$ = 10.5 Hz, $^3J_{H-H}$ = 7.8 Hz, 1H, 2-H), 5.49 (dd, 1H, $^3J_{H-H}$ = 3.4 Hz, $^3J_{H-H}$ = 1.1 Hz, 4-H), 5.21 (d, $^3J_{H-H}$ = 7.9 Hz, 1H, 1-H), 5.13 (dd, $^3J_{H-H}$ = 10.4 Hz, $^3J_{H-H}$ = 3.4 Hz, 1H, 3-H), 4.29 – 4.09 (m, 3H, 5-H + 5-CH_2), 2.19 (s, 3H, OAc), 2.12 (s, 3H, OAc), 2.08 (s, 3H, OAc), 2.02 (s, 3H, OAc).

ESI-MS: *m/z* calculated for $C_{21}H_{23}NO_{13}$ $[M+Na]^+$ 520.09, found 520.00.

Synthesis of (2*R*,3*S*,4*S*,5*R*,6*S*)-2-(acetoxymethyl)-6-(4-(hydroxymethyl)-2-nitrophenoxy)tetrahydro-2*H*-pyran-3,4,5-triyltriacetate (3)

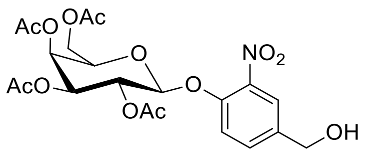

3
$C_{21}H_{25}NO_{13}$
499.42 g mol^{-1}

Aldehyde **2** (11.5 g, 23.1 mmol, 1.00 eq) was dissolved in 300 mL dry dichloromethane and 15.5 mL dry methanol (95:5). The solution was cooled to 0°C in an ice bath and sodium borohydride (1.75 g, 46.4 mmol, 2.00 eq) was added in two portions, starting with 1 eq. After stirring at 0°C for 30 minutes the other 1 eq was added and the solution was stirred for additional 20 minutes until monitoring via TLC (cyclohexane/ethyl acetate 1:1) showed a complete reaction. To stop the reaction 20 mL water was added and the solution was stirred for 10 minutes. The layers were separated and the aqueous phase was extracted four

times with 50 mL dichloromethane. The organic layer was washed with 100 mL conc. NaCl-solution and dried over Na_2SO_4. Removing the solvent gave clean alcohol **3** as a light-yellow solid.

Yield: 11.32 g (22.66 mmol, 98%).

TLC: R_f = 0.15 (cyclohexane/ethyl acetate 1:1).

^{1}H-NMR: (500 MHz, $CDCl_3$-*d*$_1$): δ (ppm) = 7.79 (d, $^4J_{H-H}$ = 1.4 Hz, 1H, Ph-H3), 7.51 (dd, $^3J_{H-H}$ = 8.5 Hz, $^4J_{H-H}$ = 1.5 Hz, 1H, Ph-H5), 7.34 (d, $^3J_{H-H}$ = 8.6 Hz, 1H, Ph-H6), 5.52 (dd, $^3J_{H-H}$ = 10.4 Hz, $^3J_{H-H}$ = 8.0 Hz, 1H, 2-H), 5.45 (d, $^3J_{H-H}$ = 3.2 Hz, 1H, 4-H), 5.09 (dd, $^3J_{H-H}$ = 10.5 Hz, $^3J_{H-H}$ = 3.4 Hz, 1H, 3-H), 5.04 (d, $^3J_{H-H}$ = 7.9 Hz, 1H, 1-H), 4.71 (s, 2H, Ph-CH_2), 4.24 (dd, $^3J_{H-H}$ = 11.3 Hz, $^3J_{H-H}$ = 7.0 Hz, 1H, 5-CH_2), 4.18 – 4.04 (m, 2H, 5-H + 5-CH_2), 2.18 (s, 3H, OAc), 2.12 (s, 3H, OAc), 2.06 (s, 3H, OAc), 2.00 (s, 3H, OAc).

^{13}C{^{1}H}-NMR: (126 MHz, $CDCl_3$-*d*$_1$): δ (ppm) = 170.5 (OAc-C_{sp2}), 170.3 (OAc-C_{sp2}), 170.3 (OAc-C_{sp2}), 169.6 (OAc-C_{sp2}), 148.5 (Ph-C1), 141.5 (Ph-C2), 137.3 (Ph-C4), 131.9 (Ph-C5), 123.3 (Ph-C3), 120.1 (Ph-C6), 101.0 (1-CH), 71.5 (5-CH), 70.7 (3-CH), 68.0 (2-CH), 66.9 (4-CH), 63.5 (Ph-CH_2), 61.5 (5-CH_2), 20.8 (OAc-*C*H_3) 20.8 (OAc-*C*H_3), 20.8 (OAc-*C*H_3), 20.7 (OAc-*C*H_3).

MALDI-HRMS: *m/z* calculated for $C_{21}H_{25}NO_{13}$ $[M+Na]^+$ 522.12181, found 522.12080 (Δ_m = 0.00101, Δ_m/m = 1.9 ppm).

Phosphate Frosting

Synthesis of (2*R*,3*S*,4*S*,5*R*,6*S*)-2-(acetoxymethyl)-6-(4-(((bis(diisopropylamino)phosphino)oxy)methyl)-2-nitrophenoxy)tetrahydro-*2H*-pyran-3,4,5-triyl triacetate

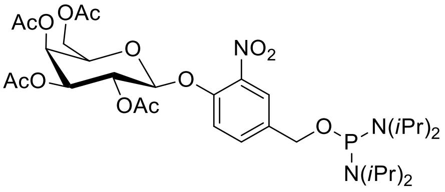

4
$C_{33}H_{52}N_3O_{13}P$
729.75 g mol^{-1}

Bis(diisopropylamino)chlorophosphine (588 mg, 2.20 mmol, 1.10 eq) was dissolved in 20 mL dry tetrahydrofuranDC and triethylamine (1.25 mL, 9.01 mmol, 4.50 eq) was added. The solution was cooled to -10°C and **3** (1.00 g, 2.00 mmol, 1.00 eq) diluted in 25 mL dry tetrahydrofuran was added dropwise for about 10 minutes. The solution was stirred for 20 h at -10°C to room temperature. The solvent was removed under reduced pressure and the crude mixture was purified by silica gel column chromatography (cyclohexane/ethyl acetate 1:1). Diamidite **4** was obtained as a colorless foam.

Yield: 329 mg (0.451 mmol, 23%).

TLC: R_f = 0.67 (cyclohexane/ethyl acetate 1:1).

^{1}H-NMR: (500 MHz, DMSO-d_6): δ (ppm) = 7.85 (d, $^4J_{H-H}$ = 2.1 Hz, 1H, Ph-H3), 7.67 (dd, $^3J_{H-H}$ = 8.7, $^4J_{H-H}$ = 2.1 Hz, 1H, Ph-H5), 7.40 (d, $^3J_{H-H}$ = 8.7 Hz, 1H, Ph-H6), 5.57 (d, $^3J_{H-H}$ = 7.5 Hz, 1H, 1-H), 5.36 (dd, $^3J_{H-H}$ = 3.2, $^3J_{H-H}$ = 1.0 Hz, 1H, 4-H), 5.29 – 5.20 (m, 2H, 2-H + 3-H), 4.61 (d, $^3J_{H-H}$ = 8.1 Hz, 2H, Ph-CH_2), 4.50 – 4.45 (m, 1H, 5-H), 4.18 – 4.09 (m, 2H, 5-CH_2), 3.59 – 3.50 (m, 4H, *i*Pr-CH), 2.15 (s, 3H, OAc), 2.03 (s, 3H, OAc), 2.02 (s, 3H, OAc), 1.94 (s, 3H, OAc), (dd, $^3J_{H-H}$ = 6.7 Hz, $^4J_{H-H}$ = 2.3 Hz, 24 H, 8x *i*Pr-CH_3).

$^{13}C\{^1H\}$-NMR: (126 MHz, DMSO-d_6): δ (ppm) = 169.9 (OAc-C_{sp2}), 169.8 (OAc-C_{sp2}), 169.5 (OAc-C_{sp2}), 168.9 (OAc-C_{sp2}), 147.4 (Ph-C1), 140.0 (Ph-C2), 135.8 (Ph-C4), 131.9 (Ph-C5), 122.5 (Ph-C3), 117.7 (Ph-C6), 98.6 (1-CH), 70.8 (5-CH), 69.9 (3-CH), 67.7 (2-CH), 67.1 (4-CH), 63.9 (Ph-CH_2), 61.3 (5-CH_2), 44.1 (*i*Pr-CH), 44.0 (*i*Pr-CH), 24.3 (*i*Pr-CH_3), 24.3 (*i*Pr-CH_3), 23.8 (*i*Pr-CH_3), 23.7 (*i*Pr-CH_3), 20.4 (OAc-CH_3), 20.4 (OAc-CH_3), 20.3 (OAc-CH_3), 20.3 (OAc-CH_3).

$^{31}P\{^1H\}$-NMR: (202 MHz, DMSO-d_6): δ (ppm) = 122.0 (s).

MALDI-HRMS: *m/z* calculated for $C_{33}H_{52}N_3O_{13}P$ $[M+OH]^+$ 746.3260, found 746.3305 (Δ_m = 0.0045, Δ_m/m = 6.0 ppm).

m/z calculated for $C_{27}H_{40}N_2O_{14}P$ $[(M-(NiPr)+H_2O]^+$ 647.2212, found 647.2258 (Δ_m = 0.0046, Δ_m/m = 7.1 ppm).

The exact mass of **4** could not be obtained. In the resulting mass spectra, similar signals were found that could be assigned to hydrolyzed compounds, even if with a deviation of more than 5 ppm.

Synthesis of (2*R*,3*S*,4*S*,5*R*,6*S*)-2-(acetoxymethyl)-6-(4-((((((2*R*,3*S*,5*R*)-2-((bis(4-methoxyphenyl)(phenyl)-methoxy)-methyl)-5-(5-methyl-2,4-dioxo-3,4-dihydropyrimidin-1(2*H*)-yl)tetrahydrofuran-3-yl)oxy)(diiso-propylamino)phosphino)-oxy)methyl)-2-nitrophenoxy)tetrahydro-2*H*-pyran-3,4,5-triyl triacetate (5)

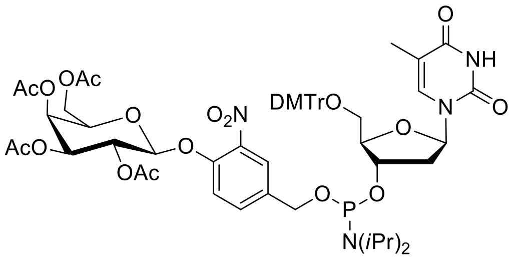

5
$C_{58}H_{69}N_4O_{20}P$
1173.16 g mol^{-1}

Phosphordiamidite **4** (285 mg, 394 µmol, 1.00 eq), 5′-*O*-(4,4′-dimethoxytrityl) thymidine (234 mg, 429 µmol, 1.09 eq) and 4,5-dicyanoimidazole (36.9 mg, 313 µmol, 0.80 eq) were diluted in 10 mL dry dichloromethane in a microwave vial. The reaction was warmed up to 40 °C for 2 hours under microwave irradiation. The solvent was directly removed under reduced pressure. Purification by silica gel column chromatography (cyclohexane/acetone 3:2 → 1:1) gave β-gal modified dT phosphoramidite **5** as a colorless foam.

Yield: 184 mg (0.157 mmol, 40%).

TLC: R_f = 0.48 (cyclohexane/acetone 1:1).

^{1}H-NMR: (500 MHz, DMSO-*d*$_6$): δ (ppm) = 11.35 (s, 1H, thymine NH), 7.82 + 7.73 (2x d, $^4J_{H\text{-}H}$ = 2.0 Hz, Σ = 1H, Ph-H3), 7.65 + 7.55 (2x dd, $^3J_{H\text{-}H}$ = 8.7 Hz, $^4J_{H\text{-}H}$ = 2.0 Hz, Σ = 1H, Ph-H5), 7.53 + 7.51 (2x d, $^4J_{H\text{-}H}$ = 0.9 Hz, Σ = 1H, thymine H6), 7.40 + 7.37 (1x d + n.d., $^3J_{H\text{-}H}$ = 8.7 Hz, 1H, Ph-H6), 7.39 – 7.35 (m, 2H, DMTr-H_{arom}), 7.30 – 7.20 (m, 7H, DMTr-H_{arom}), 6.89 – 6.83 (m, 4H, DMTr-H_{arom}), 6.20 (q, $^3J_{H\text{-}H}$ = 7.0 Hz, 1'-H), 5.55 (2x d, $^3J_{H\text{-}H}$ = 7.5 Hz, 1H, 1-H), 5.38 – 5.35 (m, 1H, 4-H), 5.30 – 5.20 (m, 2H, 2-H + 3-H), 4.74 – 4.64 (m, 1H, 3'-H), 4.57 (d, $^3J_{H\text{-}H}$ = 8.5 Hz, 2H, Ph-CH_2), 4.52 – 4.43 (m, 1H, 5-CH), 4.16 – 4.11 (m, 2H, 5-CH_2), 4.10 – 4.00 (m, 1H, 4'-H), 3.72 (s, 3H, DMTr-OCH_3), 3.70 (s, 3H, DMTr-OCH_3), 3.63 – 3.47 (m, 2H, 2x *i*Pr-CH), 3.29 – 3.18 (m, 2H, 4'-CH_2), 2.44 – 2.24 (m, 2H, 2x 2'-H), 2.15 (2x s, Σ = 3H, OAc), 2.02 (s, 3H, OAc), 2.00 + 1.99 (2x s, Σ = 3H, OAc), 1.95 (2x s, Σ = 3H, OAc), 1.50 + 1.47 (2x d, $^4J_{H\text{-}H}$ = 0.9 Hz, Σ = 3H, thymine CH_3), 1.14 – 1.07 (m, 9H, 3x *i*Pr-CH_3), 0.99 (d, $^3J_{H\text{-}H}$ = 6.7 Hz, 3H, *i*Pr-CH_3).

^{13}C{^{1}H}-NMR: (126 MHz, DMSO-*d*$_6$): δ (ppm) = 169.9 (OAc-C_{sp2}), 169.8 (d, OAc-C_{sp2}), 169.5 (OAc-C_{sp2}), 168.8 (OAc-C_{sp2}), 163.7 (thymine C4), 158.2 (d, DMTr-*C*OCH$_3$), 150.3 (thymine C2), 147.6 (d, Ph-C1), 144.6 (d, DMTr-C), 140.04 (d, Ph-C2), 135.8 (thymine C6), 135.3 (d, DMTr-C), 135.1 (d, DMTr-C), 134.6 (Ph-C4), 132.2 (d, Ph-C5), 129.7 (DMTr-C), 127.9 (DMTr-C), 127.6 (DMTr-C), 126.8 (DMTr-C), 122.7 (d, Ph-C3), 117.8 (d, Ph-C6), 113.20 (DMTr-C), 109.70 (d, thymine C5), 98.6 (d, 1-CH), 86.0 (DMTr-C) 85.9 (DMTr-C), 84.5 (d, 4'-CH), 84.0 (d, 1'-CH), 70.8 (5-CH), 69.9 (3-CH), 67.7 (2-CH), 67.1 (4-CH), 63.4 (d, 4'-CH_2), 63.2 (d, 3'-CH), 61.2 (5-CH_2), 55.0 (d, DMTr-O*C*H$_3$), 55.0 (d, DMTr-O*C*H$_3$), 42.7 (d, *i*Pr-CH), 42.6 (d, *i*Pr-CH), 38.4 (d, 2'-CH_2), 24.3 – 24.1 (m, 4x *i*Pr-CH_3), 20.4 (d, OAc-*C*H$_3$), 20.4 (OAc-*C*H$_3$), 20.3 (OAc-*C*H$_3$), 20.3 (OAc-*C*H$_3$), 11.7 (d, thymine CH_3).

^{31}P{^{1}H}-NMR: (162 MHz, DMSO-*d*$_6$): δ (ppm) = 148.02 (s), 148.00 (s).

MALDI-HRMS: *m/z* calculated for $C_{58}H_{69}N_4O_{20}P$ $[M+Na]^+$ 1195.41350, found 1195.41299 (Δ_m = 0.00051, Δ_m/m = 0.4 ppm); *m/z* calculated for $C_{58}H_{69}N_4O_{20}P$ $[M+K]^+$ 1211.38744, found 1211.38646 (Δ_m = 0.00098, Δ_m/m = 0.8 ppm).

Nucleobase Frosting

Synthesis of (2*R*,3*S*,4*S*,5*R*,6*S*)-2-(acetoxymethyl)-6-(4-(((1-((2*R*,4*S*,5*R*)-4-((*tert*-butyldimethylsilyl)oxy)-5-(((*tert*-butyldimethylsilyl)oxy)methyl)tetrahydrofuran-2-yl)-5-methyl-2-oxo-1,2-dihydropyrimidin-4-yl)oxy)methyl)-2-nitrophenoxy)tetrahydro-2*H*-pyran-3,4,5-triyl triacetate (6)

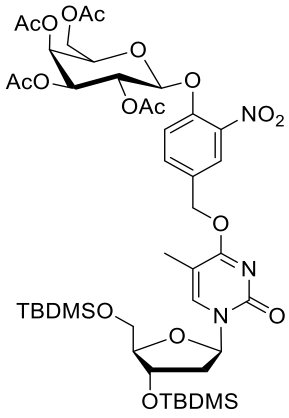

6
$C_{43}H_{65}N_3O_{17}Si_2$
952.16 g mol^{-1}

3 (3.00 g, 5.75 mmol, 1.10 eq) and triazole-activated dT (2.61 g, 5.23 mmol, 1.00 eq) was dissolved in 30 mL dry acetonitrile. 1,8-Diazabicyclo[5.4.0]undec-7-ene (DBU; 0.86 mL, 5.8 mmol, 1.1 eq) was added and the reaction mixture was stirred for 24 h at room temperature. The solvent was removed under reduced pressure. The crude mixture was purified by silica gel column chromatography using dichloromethane/methanol 95:5 to give **6** as a white-yellowish solid.

Yield: 3.64 g (3.82 mmol, 73%).

TLC: R_f = 0.46 (dichloromethane/methanol 95:5).

^{1}H-NMR: (400 MHz, CDCl$_3$-*d*$_1$): δ (ppm) = 7.85 (d, $^4J_{H-H}$ = 1.6 Hz, 1H, Ph-H3), 7.81 (s, 1H, thymine H6), 7.60 (dd, $^3J_{H-H}$ = 8.7 Hz, $^4J_{H-H}$ = 1.6 Hz, 1H, Ph-H5), 7.35 (d, $^3J_{H-H}$ = 8.6 Hz, 1H, Ph-H6), 6.32 (t, $^3J_{H-H}$ = 6.4 Hz, 1H, 1'-H), 5.54 (dd, $^3J_{H-H}$ = 10.4 Hz, $^3J_{H-H}$ = 8.0 Hz, 1H, 2-H), 5.47 (d, $^3J_{H-H}$ = 3.1 Hz, 1H, 4-H), 5.44 (s, 2H, Ph-CH$_2$), 5.10 (dd, $^3J_{H-H}$ = 10.7 Hz, $^3J_{H-H}$ = 3.6 Hz, 1H, 3-H), 5.07 (d, $^3J_{H-H}$ = 8.2 Hz, 1H, 1-H), 4.40 – 4.35 (m, 1H, 3'-H), 4.25 (dd, $^3J_{H-H}$ = 11.3 Hz, $^3J_{H-H}$ = 7.0 Hz, 1H, 5-CH$_2$), 4.20 – 4.04 (m, 2H, 5-H + 5-CH$_2$), 3.96 (d, $^3J_{H-H}$ = 2.6 Hz, 1H, 4'-H), 3.90 (dd, $^2J_{H-H}$ = 11.3 Hz, $^3J_{H-H}$ = 2.6 Hz, 1H, 4'-CH$_2$), 3.77 (dd, $^2J_{H-H}$ = 11.5 Hz, $^3J_{H-H}$ = 2.2 Hz, 1H, 4'-CH$_2$), 2.51 – 2.43 (m, 1H, 2'-H), 2.18 (s, 3H, OAc), 2.12 (s, 3H, OAc), 2.07 (s, 3H, OAc), 2.01 (s, 3H, OAc), 1.97 (s, 4H, thymine CH$_3$ + 2'-H), 0.91 (s, 9H, *t*Bu), 0.89 (s, 9H, *t*Bu), 0.11 (s, 3H, SiMe), 0.10 (s, 3H, SiMe), 0.07 (s, 3H, SiMe), 0.06 (s, 3H, SiMe).

^{13}C{^{1}H}-NMR: (126 MHz, CDCl$_3$-*d*$_1$): δ (ppm) = 170.4 (OAc-C$_{sp2}$), 170.3 (OAc-C$_{sp2}$), 170.3 (OAc-C$_{sp2}$), 169.7 (thymine C4) 169.5 (OAc-C$_{sp2}$), 155.6 (thymine C2), 149.2 (Ph-C1), 141.4 (Ph-C2),140.5 (thymine C6), 132.4 (Ph-C4), 133.6 (Ph-C5), 124.7 (Ph-C3), 119.9 (Ph-C6), 104.0 (thymine C5), 100.9 (1-CH), 88.2 (4'-CH), 86.6 (1'-CH), 71.8 (3'-CH), 71.6 (5-CH), 70.7 (3-CH), 67.9 (2-CH), 66.8 (4-CH), 66.7 (Ph-CH$_2$), 62.8 (4'-CH$_2$), 61.5 (5-CH$_2$), 42.5 (2'-CH$_2$) 26.08 (*t*Bu-CH$_3$), 25.90 (*t*Bu-CH$_3$), 20.8 (OAc-*C*H$_3$) 20.8 (OAc-*C*H$_3$), 20.8 (OAc-*C*H$_3$), 20.7 (OAc-*C*H$_3$), 18.5 (*t*Bu-*C*CH$_3$), 18.2 (*t*Bu-*C*CH$_3$), 12.4 (thymine CH$_3$), -4.42 (SiCH$_3$), -4.73(SiCH$_3$) , -5.24 (SiCH$_3$), -5.26 (SiCH$_3$).

MALDI-HRMS: *m/z* calculated for $C_{43}H_{65}N_3O_{17}$ [M+Na]$^+$ 974.37447, found 974.37344 (Δ_m = 0.00103, Δ_m/m = 1.1 ppm).

Synthesis of (2*R*,3*S*,4*S*,5*R*,6*S*)-2-(acetoxymethyl)-6-(4-(((1-((2*R*,4*S*,5*R*)-4-hydroxy-5-(hydroxymethyl)tetrahydrofuran-2-yl)-5-methyl-2-oxo-1,2-dihydropyrimidin-4-yl)oxy)methyl)-2-nitrophenoxy)tetrahydro-2*H*-pyran-3,4,5-triyl triacetate (7)

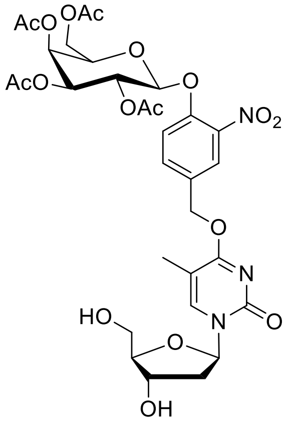

7
$C_{31}H_{37}N_3O_{17}$
723.64 g mol^{-1}

TBDMS-protected dT derivative **6** (3.64 g, 3.82 mmol, 1.00 eq) was dissolved in 30 mL dry tetrahydrofuran and glacial acetic acid (2.7 mL, 47 mmol, 12 eq) was added. The solution was cooled to 0 °C in an ice bath and tetrabutylammonium fluoride (TBAF; 1M in tetrahydrofuran; 11.5 mL, 11.5 mmol, 3.01 eq) was added. The solution was stirred for 17 h in the thawing ice bath and the solvent was then removed under reduced pressure. The residue was taken up in 100 mL ethyl acetate and was washed six times with each 25 mL of conc. NH_4Cl-solution. The aqueous phase was extracted two times with 20 mL ethyl acetate, the combined organic phase was washed two times with 25 mL water and two times with conc. NaCl-solution. After drying over Na_2SO_4 the solvent was removed under reduced pressure. The crude material was purified by silica gel column chromatography

Yield: 2.19 g (3.02 mmol, 79%).

TLC: R_f = 0.18 (dichloromethane/methanol 95:5).

^{1}H-NMR: (500 MHz, $CDCl_3$-*d*$_1$): δ (ppm) = 7.85 (d, $^4J_{H-H}$ = 2.1 Hz, 1H, Ph-H3), 7.79 (d, $^4J_{H-H}$ = 0.8 Hz, 1H, thymine H6), 7.61 (dd, $^3J_{H-H}$ = 8.6 Hz, $^4J_{H-H}$ = 2.1 Hz, 1H, Ph-H5), 7.36 (d, $^3J_{H-H}$ = 8.6 Hz, 1H, Ph-H6), 6.15 (t, $^3J_{H-H}$ = 6.4 Hz, 1H, 1'-H), 5.54 (dd, $^3J_{H-H}$ = 10.5 Hz, $^3J_{H-H}$ = 7.9 Hz, 1H, 2-H), 5.47 (dd, $^3J_{H-H}$ = 3.4 Hz, $^3J_{H-H}$ = 0.8 Hz, 1H, 4-H), 5.42 (s, 2H, Ph-CH_2), 5.11 (dd, $^3J_{H-H}$ = 10.1 Hz, $^3J_{H-H}$ = 3.8 Hz, 1H, 3-H), 5.10 (d, $^3J_{H-H}$ = 7.9 Hz, 1H, 1-H), 4.61 – 4.52 (m, 1H, 3'-H), 4.25 (dd, $^3J_{H-H}$ = 11.3 Hz, $^3J_{H-H}$ = 6.9 Hz, 1H, 5-CH_2), 4.18 – 4.09 (m, 2H, 5-H + 5-CH_2), 4.06 (dd, $^3J_{H-H}$ = 6.8 Hz, $^3J_{H-H}$ = 3.1 Hz, 1H, 4'-H), 3.94 (dd, $^2J_{H-H}$ = 12.0 Hz, $^3J_{H-H}$ = 2.0 Hz, 1H, 4'-CH_2), 3.85 (dd, $^2J_{H-H}$ = 12.0 Hz, $^3J_{H-H}$ = 3.0 Hz, 1H, 4'-CH_2), 2.49 – 2.36 (m, 2H, 2'-H), 2.18 (s, 3H, OAc), 2.12 (s, 3H, OAc), 2.06 (s, 3H, OAc), 2.01 (s, 3H, OAc), 1.97 (d, $^4J_{H-H}$ = 0.8 Hz, 4H, thymine CH_3).

^{13}C{^{1}H}-NMR: (126 MHz, $CDCl_3$-*d*$_1$): δ (ppm) = 170.5 (OAc-C_{sp2}), 170.3 (OAc-C_{sp2}), 170.3 (OAc-C_{sp2}), 170.0 (thymine C4) 169.6 (OAc-C_{sp2}), 155.9 (thymine C2), 149.2 (Ph-C1), 142.0 (Ph-C2), 141.4 (thymine C6), 133.7 (Ph-C4), 132.1 (Ph-C5), 124.9 (Ph-C3), 119.8 (Ph-C6), 104.9 (thymine C5), 100.8 (1-CH), 88.7 (4'-CH), 87.7 (1'-CH), 71.6 (5-CH), 71.2 (3'-CH), 70.7 (3-CH), 68.0 (2-CH), 66.9 (Ph-CH_2), 66.9 (4-CH), 62.3 (4'-CH_2), 61.4 (5-CH_2), 40.9 (2'-CH_2), 20.8 (OAc-*C*H_3) 20.8 (OAc-*C*H_3), 20.8 (OAc-*C*H_3), 20.7 (OAc-*C*H_3), 12.4 (thymine CH_3).

MALDI-HRMS: *m/z* calculated for $C_{31}H_{37}N_3O_{17}$ $[M+Na]^+$ 746.20152, found 746.20106 (Δ_m = 0.00046, Δ_m/m = 0.6 ppm); *m/z* calculated for $C_{31}H_{37}N_3O_{17}$ $[M+K]^+$ 762.17545, found 762.17504 (Δ_m = 0.00041, Δ_m/m = 0.5 ppm).

Synthesis of (2*R*,3*S*,4*S*,5*R*,6*S*)-2-(acetoxymethyl)-6-(4-(((1-((2*R*,4*S*,5*R*)-5-((bis(4-methoxyphenyl)(phenyl)methoxy) methyl)-4-hydroxytetrahydrofuran-2-yl)-5-methyl-2-oxo-1,2-dihydropyrimidin-4-yl)oxy)methyl)-2-nitrophenoxy)tetra-hydro-2*H*-pyran-3,4,5-triyl triacetate (8)

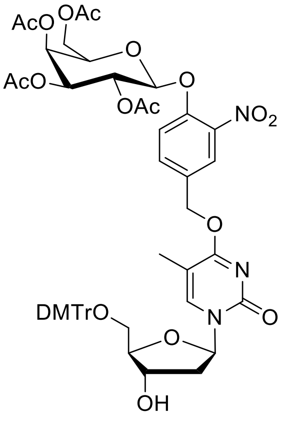

8
$C_{52}H_{55}N_3O_{19}$
1026.00 g mol^{-1}

Unprotected 2'-deoxythymidine derivative **7** (194 mg, 268 µmol, 1.00 eq) was coevaporated six times with 6 mL dry pyridine. 4,4′-Dimethoxytrityl chloride (146 mg, 431 µmol, 1.60 eq) was added and both were dissolved in 5 mL dry pyridine. After stirring for 18 h at room temperature (monitored by TLC) the reaction was stopped by adding 5 mL dry ethanol. The solvent was removed under reduced pressure and purification by silica gel column chromatography (dichloromethane/methanol 98:2 → 97:3) gave 5'-DMTr-protected **8** as a colorless foam.

Yield: 208 mg (203 µmol, 76%).

TLC: R_f = 0.24 (dichloromethane/methanol 19:1).

^{1}H-NMR: (500 MHz, $CDCl_3$-*d*$_1$): δ (ppm) = 7.94 (d, $^4J_{H-H}$ = 0.9 Hz, 1H, thymine H6), 7.83 (d, $^4J_{H-H}$ = 2.1 Hz, 1H, Ph-H3), 7.60 (dd, $^3J_{H-H}$ = 8.6 Hz, $^4J_{H-H}$ = 2.1 Hz, 1H, Ph-H5), 7.40 – 7.38 (m, 2H, DMTr-H_{arom}), 7.36 (d, $^3J_{H-H}$ = 8.6 Hz, 1H, Ph-H6), 7.30 – 7.27 (m, 6H, DMTr-H_{arom}), 7.24 – 7.20 (m, 1H, DMTr-H_{arom}), 6.82 (d, $^3J_{H-H}$ = 8.8 Hz, 4H, DMTr-H_{arom}), 6.39 (t, $^3J_{H-H}$ = 6.4 Hz, 1H, 1'-H), 5.54 (dd, $^3J_{H-H}$ = 10.5 Hz, $^3J_{H-H}$ = 7.9 Hz, 1H, 2-H), 5.47 (dd, $^3J_{H-H}$ = 3.4 Hz, $^3J_{H-H}$ = 0.7 Hz, 1H, 4-H), 5.41 (s, 2H, Ph-CH_2), 5.11 (dd, $^3J_{H-H}$ = 10.1 Hz, $^3J_{H-H}$ = 3.0 Hz, 1H, 3-H), 5.09 (d, $^3J_{H-H}$ = 7.9 Hz, 1H, 1-H), 4.58 – 4.54 (m, 1H, 3'-H), 4.24 (dd, $^2J_{H-H}$ = 11.3 Hz, $^3J_{H-H}$ = 6.9 Hz, 1H, 5-CH_2), 4.17 (dd, $^2J_{H-H}$ = 11.3 Hz, $^3J_{H-H}$ = 6.2 Hz, 1H, 5-CH_2), 4.12 (q, $^3J_{H-H}$ = 3.2 Hz, 1H, 4'-H), 4.10 – 4.06 (m, 1H, 5-H), 3.78 (s, 6H, DMTr-OCH_3), 3.48 (dd, $^2J_{H-H}$ = 10.6 Hz, $^3J_{H-H}$ = 3.1 Hz, 1H, 4'-CH_2), 3.38 (dd, $^2J_{H-H}$ = 10.6 Hz, $^3J_{H-H}$ = 3.2 Hz, 1H, 4'-CH_2), 2.65 (ddd, $^2J_{H-H}$ = 13.6 Hz, $^3J_{H-H}$ = 6.0 Hz, $^3J_{H-H}$ = 3.8 Hz, 1H, 2'-H), 2.31 – 2.23 (m, 1H, 2'-H), 2.18 (s, 3H, OAc), 2.12 (s, 3H, OAc), 2.06 (s, 3H, OAc), 2.01 (s, 3H, OAc), 1.56 (d, $^4J_{H-H}$ = 0.5 Hz, 3H, thymine CH_3).

$^{13}C\{^1H\}$-NMR: (126 MHz, $CDCl_3$-*d*$_1$): δ (ppm) = 170.4 (OAc-C_{sp2}), 170.3 (OAc-C_{sp2}), 170.3 (OAc-C_{sp2}), 169.9 (thymine C4) 169.5 (OAc-C_{sp2}), 158.8 (DMTr-*C*OCH_3), 155.8 (thymine C2), 149.2 (Ph-C1), 144.5 (DMTr-*C*), 141.4 (Ph-C2), 140.7 (thymine C6), 135.6 (DMTr-*C*), 135.6 (DMTr-*C*), 133.6 (Ph-C4), 132.3 (Ph-C5), 130.2 (DMTr-*C*), 130.2 (DMTr-*C*), 128.2 (DMTr-C), 128.1 (DMTr-C), 127.2 (DMTr-*C*), 124.8 (Ph-C3), 119.8 (Ph-C6), 113.4 (DMTr-*C*), 104.7 (thymine C5), 100.8 (1-CH), 87.0 (DMTr-*C*), 86.6 (1'-CH), 86.5 (4'-CH), 72.1 (3'-CH), 71.6 (5-CH), 70.7 (3-CH), 68.0 (2-CH), 66.9 (Ph-CH_2), 66.8 (4-CH), 63.4 (4'-CH_2), 61.5 (5-CH_2), 55.4 (DMTr-CH_3), 42.2 (2'-CH_2), 20.8 (OAc-*C*H_3) 20.8 (OAc-*C*H_3), 20.8 (OAc-*C*H_3), 20.7 (OAc-*C*H_3), 11.8 (thymine CH_3).

MALDI-HRMS: *m/z* calculated for $C_{52}H_{55}N_3O_{19}$ $[M+Na]^+$ 1048.33220, found 1048.33132 (Δm = 0.00088, Δm/m = 0.8 ppm); *m/z* calculated for $C_{52}H_{55}N_3O_{19}$ $[M+K]^+$ 1064.30613, found 1064.30549 (Δm = 0.00064, Δm/m = 0.6 ppm).

Synthesis of (2*R*,3*S*,4*S*,5*R*,6*S*)-2-(acetoxymethyl)-6-(4-(((1-((2*R*,4*S*,5*R*)-5-((bis(4-methoxyphenyl)(phenyl)methoxy)methyl)-4-(((2-cyanoethoxy)(diisopropylamino)phosphino)oxy)tetrahydrofuran-2-yl)-5-methyl-2-oxo-1,2-dihydro-pyrimidin-4-yl)oxy)methyl)-2-nitrophenoxy)tetrahydro-2*H*-pyran-3,4,5-triyl triacetate (9)

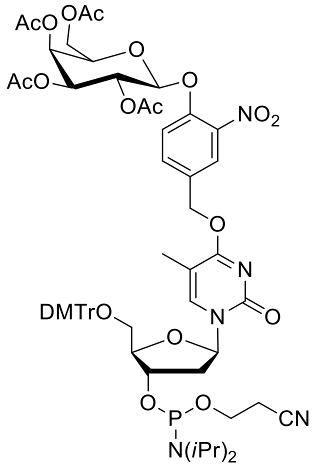

9
$C_{61}H_{72}N_5O_{20}P$
1226.22 g mol^{-1}

5'-DMTr-protected **8** (300 mg, 0.29 mmol, 1.00 eq) was dissolved in 10 mL dry dichloromethane and *N,N*-diisopropylethylamine (0.25 mL, 1.47 mmol, 5.03 eq) was added. After stirring at room temperature for 10 minutes 2-cyanoethyl-*N,N'*-diisopropylchlorophosphoramidite (78.0 µL, 0.35 mmol, 1.20 eq) was added to the reaction and the solution was stirred at room temperature for further 3 h. After controlling the reaction process via TLC another 2-cyanoethyl-*N,N'*-diisopropylchlorophosphoramidite (32.0 µL, 0.14 mmol, 0.49 eq) was added and the reaction was stirred for additional 16 h. Fresh *N,N*-diisopropylethylamine (0.05 mL, 0.29 mmol, 1.00 eq) and 2-cyanoethyl-*N,N'*-diisopropylchlorophosphoramidite (32.0 µL, 0.14 mmol, 0.49 eq) was added again and the reaction stirred for further 5 h. The reaction was stopped after a total time of 24 h by adding 5 mL 5% aqueous $NaHCO_3$-solution. The organic layer was extended to 30 mL with dichloromethane and the layers were separated. The organic layer was washed with 5 mL conc. NaCl-solution and the aqueous layer was extracted with 10 mL dichloromethane. The combined organic layers were dried over Na_2SO_4 and the solvent was removed. Phosphoramidite **9** was obtained as a colorless foam after purification by silica gel column chromatography (dichloromethane/methanol 98:2).

Yield: 219 mg (179 µmol, 61%).

TLC: R_f = 0.60 (dichloromethane/methanol 19:1).

^{1}H-NMR: (500 MHz, $CDCl_3$-d_1): δ (ppm) = 7.95 (2x d, $^4J_{H-H}$ = 0.8 Hz, Σ = 1H, thymine H6), 7.84 – 7.81 (m, Ph-H3), 7.60 (dd, $^3J_{H-H}$ = 8.7 Hz, $^4J_{H-H}$ = 2.0 Hz, 1H, Ph-H5), 7.42 – 7.37 (m, 2H, DMTr-H_{arom}), 7.35 (d, $^3J_{H-H}$ = 8.7 Hz, 1H, Ph-H6), 7.31 – 7.26 (m, 6H, DMTr-H_{arom}), 7.25 – 7.21 (m, 1H, DMTr-H_{arom}), 6.85 – 6.79 (m, 4H, DMTr-H_{arom}), 6.38 (2x t, $^3J_{H-H}$ = 6.3 Hz, Σ = 1H, 1'-H), 5.54 (dd, $^3J_{H-H}$ = 10.5 Hz, $^3J_{H-H}$ = 7.9 Hz, 1H, 2-H), 5.47 (dd, $^3J_{H-H}$ = 3.3 Hz, $^3J_{H-H}$ = 0.6 Hz 1H, 4-H), 5.43 (s, 2H, Ph-CH_2), 5.10 (dd, $^3J_{H-H}$ = 10.5 Hz, $^3J_{H-H}$ = 3.4 Hz, 1H, 3-H), 5.07 (d, $^3J_{H-H}$ = 7.9 Hz, 1H, 1-H), 4.69 – 4.59 (m, 1H, 3'-H), 4.25 (dd, $^2J_{H-H}$ = 11.4 Hz, $^3J_{H-H}$ = 6.9 Hz, 1H, 5-CH_2), 4.19 – 4.17 (m, 1H, 5-CH_2), 4.17 – 4.14 (m, 1H, 4'-H), 4.07 (t, $^3J_{H-H}$ = 6.7 Hz, 5-H), 3.85 – 3.81 + 3.76 – 3.71 (m, Σ = 1H, OCH_2CH_2CN), 3.79 (s, 3H, DMTr-OCH_3), 3.78 (s, 3H, DMTr-OCH_3), 3.65 – 3.47 (m, 4H, OCH_2CH_2CN + 2x *i*Pr-CH + 4'-CH_2), 3.36 – 3.29 (m, 1H, 4'-CH_2), 2.74 – 2.65 (m, 1H, 2'-H), 2.61 (t, $^3J_{H-H}$ = 6.3 Hz, 1H,

OCH_2CH_2CN), 2.40 (t, $^3J_{H-H}$ = 6.4 Hz, 1H, OCH_2CH_2CN), 2.33 – 2.25 (m, 1H, 2'-CH_2), 2.18 (s, 3H, OAc), 2.12 (s, 3H, OAc), 2.06 (s, 3H, OAc), 2.01 (s, 3H, OAc), 1.52 – 1.48 (m, 3H, thymine CH_3), 1.18 – 1.13 (m, 9H, 3x *i*Pr-CH_3), 1.04 (d, $^3J_{H-H}$ = 6.8 Hz, 3H, *i*Pr-CH_3).

<u>$^{13}C\{^1H\}$-NMR:</u> (126 MHz, $CDCl_3$-d_1): δ (ppm) = 170.4 (OAc-C_{sp2}), 170.3 (OAc-C_{sp2}), 170.2 (OAc-C_{sp2}), 169.8 (d, thymine C4), 169.5 (OAc-C_{sp2}), 158.9 (d, DMTr-*C*OCH_3), 155.7 (thymine C2), 149.2 (Ph-C1), 144.5 (DMTr-*C*), 141.4 (Ph-C2), 140.7 (d, thymine C6), 135.5 (DMTr-*C*), 135.5 (DMTr-*C*), 133.6 (Ph-C5), 132.4 (Ph-C4), 130.4 (DMTr-*C*), 130.3 (DMTr-*C*), 130.3 (DMTr-*C*), 128.4 (DMTr-*C*), 128.3 (DMTr-*C*), 128.1 (DMTr-*C*), 127.3 (d, DMTr-*C*), 124.7 (Ph-C3), 119.9 (Ph-C6), 117.7 (d, CH_2CN), 113.4 (DMTr-*C*), 104.6 (d, thymine C5), 100.9 (1-CH), 87.0 (DMTr-C), 87.0 (DMTr-C), 86.5 (d, 4'-CH), 85.7 (2x d, 4'-CH), 73.0 (2x d, 3'-CH), 71.6 (5-CH), 70.7 (3-CH), 68.0 (2-CH), 66.9 (4-CH), 66.7 (Ph-CH_2), 62.8 (d, 4'-CH_2), 61.5 (5-CH_2), 58.4 (2x d, OCH_2CH_2CN), 55.4 (DMTr-CH_3), 55.4 (DMTr-CH_3), 43.4 (d, *i*Pr-CH), 43.3 (d, *i*Pr-CH), 41.1 (2x d, 2'-CH_2), 24.8 (*i*Pr-CH_3), 24.7 (*i*Pr-CH_3), 24.7 (*i*Pr-CH_3), 24.6 (*i*Pr-CH_3), 20.8 (OAc-CH_3), 20.8 (OAc-CH_3), 20.8 (OAc-CH_3), 20.7 (OAc-CH_3), 20.6 (d, OCH_2CH_2CN), 20.3 (d, OCH_2CH_2CN),11.7 (d, thymine CH_3).

<u>$^{31}P\{^1H\}$-NMR:</u> (202 MHz, $CDCl_3$-d_1): δ (ppm) = 149.1 (s), 148.5 (s).

<u>MALDI-HRMS:</u> *m/z* calculated for $C_{61}H_{72}N_5O_{20}P$ $[M+Na]^+$ 1248.44005, found 1248.43893 (Δm = 0.00112, Δm/m = 0.9 ppm).

2. Alkyne-Modified 5'-Frosting

Synthesis of 4-(1-hydroxybut-3-yn-1-yl)-2-nitrophenol (10)

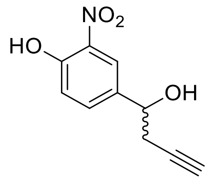

10
$C_{10}H_9NO_4$
207.18 g mol^{-1}

Zinc dust (2.93 g, 44.9 mmol, 2.50 eq) was heated in a vacuum, after cooling-down 50 mL of dry *N*,*N*-dimethylformamide was added under an argon atmosphere and the suspension was cooled to 0°C in an ice bath. Propargyl bromide (80% in toluene; 3.0 mL, 32 mmol, 1.8 eq) was added dropwise at 0°C and the reaction mixture was stirred for 1 h under cooling. 4-Hydroxy-3-nitrobenzaldehyde (3.0 g, 18. mmol, 1.0 eq) was dissolved in 20 mL dry *N*,*N*-dimethylformamide, added slowly to the cooled reaction mixture and stirred at 0°C for 1 h. After removing the ice bath the solution was stirred for additional 17 h at room temperature. The reaction was finished by adding water and the remaining solid parts were removed by filtration. The aqueous phase was extracted eleven times with diethyl ether and three times with dichloromethane. The combined organic layer was dried over Na_2SO_4 and the solvent was removed. The crude product was purified by silica gel column chromatography (cyclohexane/ethyl acetate 1:1). The alkyne-modified self-immolative linker precursor **10** was obtained as a yellow oil.

Yield: 1.59 g (7.67 mmol, 43%).

TLC: R_f = 0.59 (cyclohexane/ethyl acetate 1:1).

^{1}H-NMR: (500 MHz, $CDCl_3$-d_1): δ (ppm) = 10.56 (s, 1H, OH), 8.15 (d, $^4J_{H-H}$ = 2.0 Hz, 1H, Ph-H3), 7.64 (dd, $^3J_{H-H}$ = 8.7 Hz, $^4J_{H-H}$ = 2.2 Hz, 1H, Ph-H5), 7.16 (d, $^3J_{H-H}$ = 8.7 Hz, 1H, Ph-H6), 4.88 (t, $^3J_{H-H}$ = 6.2 Hz, 1H, C*H*OH), 2.71 – 2.59 (m, 2H, CH_2CCH), 2.10 (t, $^3J_{H-H}$ = 2.6 Hz, 1H, CH_2CC*H*).

^{13}C{^{1}H}-NMR: (126 MHz, $CDCl_3$-d_1): δ (ppm) = 154.8 (Ph-C1), 135.3 (Ph-C4), 135.0 (Ph-C5), 133.4 (Ph-C2), 122.4 (Ph-C3), 120.3 (Ph-C6), 79.7 (CH_2CCH), 72.0 (CH_2C*C*H), 70.9 (*C*HOH), 29.5 (*C*H_2CCH).

ESI-MS: *m/z* calculated for $C_{10}H_9NO_4$ [M+H]$^+$ 208.06, found 206.18.

Synthesis of (2*R*,3*S*,4*S*,5*R*,6*S*)-2-(acetoxymethyl)-6-(4-(1-hydroxybut-3-yn-1-yl)-2-nitrophenoxy)tetrahydro-2*H*-pyran-3,4,5-triyl triacetate (11)

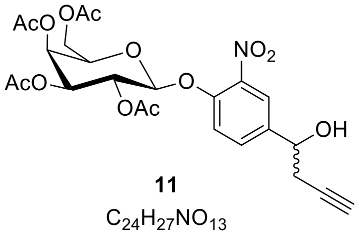

11
$C_{24}H_{27}NO_{13}$
537.47 g mol^{-1}

2,3,4,6-Tetra-*O*-acetyl-α-D-galactopyranosyl bromide **1** (8.83 g, 21.5 mmol, 1.48 eq) was dissolved in 200 mL dry acetonitrile and Ag_2CO_3 (14.8 g, 53.7 mmol, 3.70 eq) was added. After stirring the suspension for 30 min at room temperature 1,1,4,7,10,10-hexamethyltriethylenetetramine (HMTETA; 2.5 mL, 9.2 mmol, 0.63 eq) was added and stirred for another 30 min. Alkyne-modified **10** (3.00 g, 14.5 mmol, 1.00 eq) was dissolved in 30 mL dry acetonitrile and added slowly to the reaction mixture, which was stirred at room temperature for 16 h. The suspension was filtered through celite and washed with ethyl acetate until the filtrate was clear. The solvent was removed und the orange residue was dissolved in ethyl acetate. The organic layer was washed two times with water, two times with conc. Na_2CO_3-solution and two times with conc. NaCl-solution. The aqueous layer was extracted two times with ethyl acetate and the combined organic layer was dried over Na_2SO_4. The solvent was removed and the crude mixture was purified by silica gel column chromatography (cyclohexane/ethyl acetate 1:1). β-Galactoside **11** was obtained as a dark-yellow foam.

Yield: 6.85 g (12.7 mmol, 88%).

TLC: R_f = 0.30 (cyclohexane/ethyl acetate 1:1).

^{1}H-NMR: (400 MHz, $CDCl_3$-d_1): δ (ppm) = 7.85 (2x d, $^4J_{H-H}$ = 2.1 Hz, 1H, Ph-H3), 7.56 (2x dd, $^3J_{H-H}$ = 8.6 Hz, $^4J_{H-H}$ = 2.1 Hz, Ph-H5), 7.34 (2x d, $^3J_{H-H}$ = 8.6 Hz, 1H, Ph-H6), 5.53 (dd, $^3J_{H-H}$ = 10.5 Hz, $^3J_{H-H}$ = 7.9 Hz, 1H, 2-H), 5.47 – 5.45 (m, 1H, 4-H), 5.09 (dd, $^3J_{H-H}$ = 10.5 Hz, $^3J_{H-H}$ = 3.4 Hz, 1H, 3-H), 5.06 (2x d, $^3J_{H-H}$ = 8.0 Hz, 1H, 1-H), 4.93 – 4.87 (m, 1H, C*H*OH), 4.28 – 4.22 (m, 1H, 5-CH_2), 4.19 – 4.10 (m, 1H, 5-CH_2), 4.11 – 4.03 (m, 1H, 5-H), 2.66 – 2.61 (m, 2H, C*H*$_2$CCH), 2.56 – 2.49 (m, 1H, CH_2CC*H*), 2.18 (s, 3H, OAc), 2.12 (s, 3H, OAc), 2.11– 2.09 (m, 1H, C*H*$_2$CCH), 2.06 (s, 3H, OAc), 2.01 (s, 3H, OAc).

^{13}C{^{1}H}-NMR: (101 MHz, $CDCl_3$-d_1): δ (ppm) = 170.5 (OAc-C_{sp2}), 170.3 (d, OAc-C_{sp2}), 170.3 (OAc-C_{sp2}), 169.6 (OAc-C_{sp2}), 148.9 (d, Ph-C1), 141.2 (d, Ph-C2), 138.8 (d, Ph-C4), 131.1 (d, Ph-C5), 122.8 (d, Ph-C3), 119.9 (m, Ph-C6), 100.9 (d, 1-CH), 79.6 (m, CH_2*C*CH), 72.1 (d, CH_2C*C*H), 71.5 (5-CH), 70.8 (d, C*H*OH), 70.7 (d, 3-CH), 68.0 (2-CH), 66.8 (d, 4-CH), 61.5 (m, 5-CH_2), 29.6 (*C*H_2CCH), 20.9 (m, OAc-*C*H_3), 20.8 (m, OAc-*C*H_3), 20.8 (m, OAc-*C*H_3), 20.7 (m, OAc-*C*H_3).

ESI-MS: *m/z* calculated for $C_{24}H_{27}NO_{13}$ $[M+Na]^+$ 560.14, found 560.13.

Synthesis of (2*R*,3*S*,4*S*,5*R*,6*S*)-2-(acetoxymethyl)-6-(4-(1-(((2-cyanoethoxy)(diisopropylamino)phosphino)oxy)but-3-yn-1-yl)-2-nitrophenoxy)tetrahydro-*2H*-pyran-3,4,5-triyl triacetate (12)

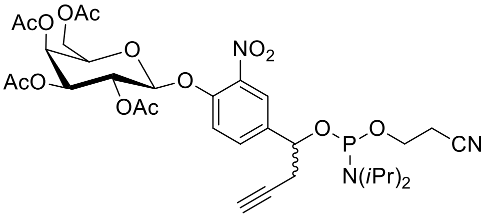

12
$C_{33}H_{44}N_3O_{14}P$
737.69 g mol^{-1}

Alcohol **11** (450 mg, 837 µmol, 1.00 eq) was dissolved in 10 mL dry dichloromethane and *N*,*N*-diisopropylethylamine (0.70 mL, 4.2 mmol, 4.9 eq) was added. After stirring at room temperature for 10 minutes 2-cyanoethyl-*N*,*N'*-diisopropylchlorophosphoramidite (0.20 mL, 0.90 mmol, 1.1 eq) was added and the solution was stirred at room temperature for 18 h. The solvent was removed and the crude material was purified by silica gel column chromatography (cyclohexane/ethyl acetate 2:1 → 1:2). Phosphoramidite **12** was obtained as a colorless foam.

Yield: 229 mg (315 µmol, 38%).

TLC: R_f = 0.52 (cyclohexane/ethyl acetate 1:1).

^{1}H-NMR: (500 MHz, $CDCl_3$-d_1): δ (ppm) = 7.88 – 7.79 (m, 1H, Ph-H3), 7.60 – 7.51 (m, Ph-H5), 7.33 (2x d, $^3J_{H\text{-}H}$ = 8.6 Hz, 1H, Ph-H6), 5.54 (2x dd, $^3J_{H\text{-}H}$ = 10.3 Hz, $^3J_{H\text{-}H}$ = 8.0 Hz, 1H, 2-H), 5.47 (d, $^3J_{H\text{-}H}$ = 3.2 Hz, 1H, 4-H), 5.12 – 5.04 (m, 2H, 3-H + 1-H), 4.99 – 4.82 (m, 1H, C*H*OP), 4.30 – 4.22 (m, 1H, 5-CH_2), 4.20 – 4.12 (m, 1H, 5-CH_2), 4.11 – 4.03 (m, 1H, 5-H), 3.98 . 3.83 (m, 1H, O$CH_2$$CH_2$CN), 3.81 – 3.61 (m, 2H, OC$H_2$$CH_2$CN + *i*Pr-CH), 3.60 – 3.52 (m, 1H, *i*Pr-CH), 2.79 – 2-57 (m, 3H, OCH_2CH_2CN + CH_2CCH), 2.56 – 2.49 (m, 1H, CH_2CC*H*), 2.19 (2x s, 3H, OAc), 2.13 (2x s, 3H, OAc), 2.07 (2x s, 3H, OAc), 2.03 – 1.98 (m, 4H, OAc + CH_2CCH), 1.24 (d, $^3J_{H\text{-}H}$ = 6.8 Hz, *i*Pr-CH_3), 1.17 (2x d, $^3J_{H\text{-}H}$ = 6.8 Hz, 2x *i*Pr-CH_3), 1.00 (2x d, $^3J_{H\text{-}H}$ = 6.8 Hz, *i*Pr-CH_3).

^{13}C{^{1}H}-NMR: (126 MHz, $CDCl_3$-d_1): δ (ppm) = 170.4 (OAc-C_{sp2}), 170.3 (d, OAc-C_{sp2}), 170.3 (OAc-C_{sp2}), 169.6 (OAc-C_{sp2}), 148.9 (d, Ph-C1), 141.2 (m, Ph-C2), 138.1 (m, Ph-C4), 131.8 (d, Ph-C5), 123.4 (m, Ph-C3), 119.6 (m, Ph-C6), 117.7 (d, CH_2CN), 100.9 (d, 1-CH), 79.5 (m, CH_2*C*CH), 72.9 (m, CH_2C*C*H), 71.6 (5-CH), 71.5 (d, C*H*OP), 70.7 (d, 3-CH), 68.0 (2-CH), 66.9 (d, 4-CH), 61.4 (m, 5-CH_2), 58.5 (2x d, O*C*$H_2$$CH_2$CN), 43.5 (m, 2x *i*Pr-CH), 29.3 (m, *C*H_2CCH), 24.8 (*i*Pr-CH_3), 24.7 (*i*Pr-CH_3), 24.5 (*i*Pr-CH_3), 24.4 (*i*Pr-CH_3), 20.8 (OAc-*C*H_3), 20.8 (OAc-*C*H_3), 20.8 (OAc-*C*H_3), 20.7 (OAc-*C*H_3), 20.5 (m, , OCH_2*C*H_2CN).

^{31}P{^{1}H}-NMR: (162 MHz, $CDCl_3$-d_1): δ (ppm) = 149.2 (s), 149.2 (s), 148.6 (s), 148.6 (s).

MALDI-HRMS: *m/z* calculated for $C_{33}H_{44}N_3O_{14}P$ $[M+H]^+$ 738.2634, found 738.2638 (Δm = 0.0004, Δm/m = 0.05 ppm).

3. Oligonucleotide Synthesis

Materials and Methods

All oligonucleotide handling was performed using RNAse free water. Therefore, 0.1% diethyl pyrocarbonate (DEPC) was added to Milli-Q water, stirred overnight and autoclaved for 15 minutes at 121°C.

An ABI 392 DNA/RNA synthesizer was used for the synthesis of all oligonucleotides. The solid-phase synthesis scale for **ON2**, **ON3** and **ON4** was each 1.0 µmol. As activator reagent 0.3 M 5-(benzylthio)-1*H*-tetrazole in acetonitrile (emp Biotech) was used. Also UltraMild capping reagents (tetrahydrofuran/pyridine/phenoxyacetic anhydride, emp Biotech) were used. Natural DNA phosphoramidites were coupled within 30 s. The coupling time for glycosylated phosphoramidites was extended to 6 min. As detritylation reagent 3% trichloroacetic acid (TCA) in dichloromethane (emp Biotech) was used. Oxidation was made of *Oxidizing (ABI)* purchased from J.T. Baker. **ON2** and **ON3** were synthesized with a DMTr-ON strategy, **ON4** with DMTr-Off.

All unmodified DNA phosphoramidites contained a 5'-DMTr protecting group and the respective nucleobases were protected with UltraMild® protecting groups (**ON2** and **ON3**), which enable a cleavage under moderate conditions. **ON4** was synthesized using benzoyl-protected adenosine. The individual components and their suppliers are listed in Table S1.

Table S1. Overview of the commercially available phosphoramidites and the solid-phase material used for the synthesis of **ON2**, **ON3** and **ON4**.

synthesis of	description	supplier
**ON2**, **ON3**, **ON4**	DMT dT phosphoramidite	BioSearch Technologies
**ON2**, **ON3**, **ON4**	iPr-Pac-dG-CE phosphoramidite	LGC LINK
**ON2**, **ON3**, **ON4**	Ac-dC-CE phosphoramidite	Glen Research
**ON2**, **ON3**	deoxy Adenosine (n-Pac) CED phosphoramidite	ChemGenes
**ON4**	deoxy Adenosine (n-bz) CED phosphoramidite	ChemGenes
**ON2**, **ON3**	iPr-Pac-dG-SynBase™ CPG 1000/110 (1 µm)	LGC LINK
**ON4**	Alkyne CPG 1000A	Lumiprobe

The chemical structure of Alkyne CPG 1000A is showed in Scheme S1.

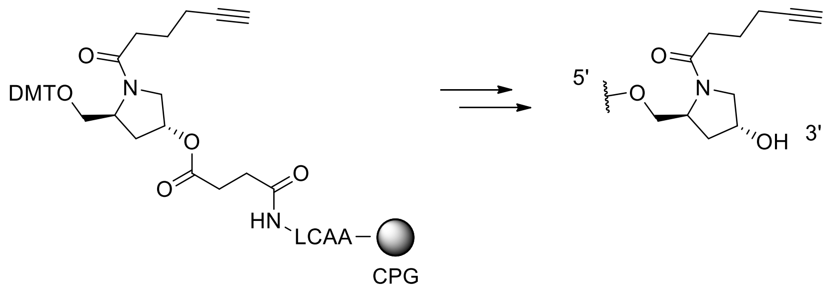

Alkyne CPG 1000A

Scheme S1. Alkyne-modified CPG-material (left, *Lumiprobe*) and released chemical structure in **ON4** (right).

The commercially available phosphoramidites were used at 0.1 M concentrations in dry acetonitrile (*Acros Organics*). β-gal modified phosphoramidites **5** and **9** were also used at 0.1 M concentration in dry acetonitrile (**5**) or acetonitrile/dichloromethane 9:1 (**9**, insufficient solubility in pure acetonitrile). Phosphoramidite **12** was used at higher concentration of 0.12 M in dry acetonitrile.

The unmodified sense and antisense strands **ON1** and **ON5** were purchased HPLC-purified from *Biomers.* The sequences are given in Table S2.

Table S2. Overview of used oligonucleotides **ON1**-**ON5**. **ON1** and **ON5** were purchased from Biomers. **ON2**, **ON3** and **ON4** were produced internally using the described procedures.

	sequence (5' → 3')	calculated exact mass [Da]	calculated average mass [Da]	found average mass [Da]
**ON1**	GCA TAA ATA AAG GTG	4646.8420	4649.0663	4648.7031
**ON2**	GCA TAA AT^{p}A AAG GTG	4959.9218	4963.3264	4961.7773
**ON3**	GCA TAA AT^{nb}A AAG GTG	4959.9218	4962.3264	4961.8086
**ON4**	5'$_{f}^{alkyne}$ GCA TAA ATA AAG GTG 3'^{alkyne}	5350.9804	5353.5765	5352.9722
**ON5**	CAC CTT TAT TTA TGC	4490.7773	4492.9406	4492.6479

ON2 and **ON3** were synthesized using DMTr-On strategy. The synthesis of **ON4** ended with a component where no DMTr-group was possible. The individual further steps for cleavage and purification are described later after introducing the instrument and methods.

Purification

RP-HPLC

HPLC was performed using an Agilent 1200 system (*Agilent Technologies*), Agilent 1260 Infinity system (*Agilent Technologies*) and ÄKTApurifier (*Cytivia*). All oligonucleotides were eluted by RP-HPLC using 400 mM hexafluoroisopropanol (HFIP, *fluorochem*), 16.3 mM triethylamine (TEA, BioUltra ≥ 99.5%, *Sigma-Aldrich*) buffer (pH 7.8) against methanol (LiChrosolv® from *Merck* or HiPerSolv CHROMANORM® from *VWR*). The buffer was prepared in-house based on RNAse free water. Elution was carried out via XBridge columns from *Waters*, the list is shown in Table S3.

Table S3. XBridge Columns (Waters) used for RP-HPLC purification and analytical runs.

column	name
1	XBridge BEH C18 OBD Prep Column, 130 Å, 5 µm, 10 x 50 mm
2	XBridge BEH Peptide C18 OBD Prep Column, 300 Å, 5 µm, 10 x 250 mm
3	XBridge peptide BEH C18 Column, 300 Å, 3.5 µm, 4.6 x 250 mm

Different gradients were run depending on the application, the properties of the column and the HPLC device. The detailed gradients are shown in Table S4.

Table S4. RP-HPLC gradients for oligonucleotide elution.

condition number	device	column	methanol [%]	flow rate [mL min^{-1}]	temperature [°C]
1	ÄKTApurifier	1	0-3.33 min: 5%, 3.33-21.33 min: 5-60%, 21.33-25.33 min: 60-100%, 25.33-29 min: 100%	4.0	20
2	Agilent	2	0-2 min: 5%, 2-5 min: 5-15%, 5-30 min: 15-25%, 30-35 min: 25-100%, 35-40 min: 100%	3.0	60
3	Agilent	2	0-2 min: 5%, 2-5 min: 5-15%, 5-35 min: 15-30%, 35-40 min: 30-100%, 40-45 min: 100%	3.0	60
4	Agilent	3	0-2 min: 5%, 2-4 min: 5-15%, 4-24 min: 15-25%, 24-30 min: 25-100%, 30-36 min: 100%	0.7	60
5	Agilent	3	0-2 min: 5%, 2-4 min: 5-15%, 4-34 min: 15-30%, 34-40 min: 30-100%, 40-46 min: 100%	0.7	60

Mass Spectrometry

Oligonucleotide identity and purity was verified by LC-MS via Orbitrap Exploris 120 Mass Spectrometer from *ThermoFisher Scientific.* Preliminary chromatography was performed via UHPLC (Vanquish^{TM} Flex, *ThermoFisher Scientific*) equipped with column ACQUITY^{TM} Premier Peptide BEH C18, 300 Å, 1.7 µm, 2.1 x 150 mm from *Waters*.

Individual purification of ON2, ON3 and ON4

ON2

Phosphate-modified **ON2** was synthesized with DMTr-On. Remaining solvent from synthesis was removed under reduced pressure. Cleavage and deprotection was carried out in 500 µL 0.05 M K_2CO_3 in methanol for four hours at 30°C (500 rpm). The solid phase was filtrated off via centrifugal filters (*VWR*) and washed with HFIP/TEA buffer. The resulting solution has been extended to an overall ratio of 95:5 HFIP/TEA buffer against methanol (starting condition for RP-HPLC). DMTr-On purification was performed using condition 1. Obtained product fractions were combined and the solvent was evaporated in a vacuum concentrator. DMTr was cleaved by incubation with 1 mL of 80% acetic acid for 25 minutes at 25°C (500 rpm), following immediate solvent removal in a vacuum concentrator. DMtr-Off purification was performed using condition 2 and 4.

ON3

Nucleobase-modified **ON3** was synthesized with DMTr-On. Remaining solvent from synthesis was removed under reduced pressure. Cleavage and deprotection was carried out in 500 µL 0.05 M K_2CO_3 in methanol for five hours at 30°C (500 rpm). The solid phase was filtrated off via centrifugal filters (*VWR*) and washed with HFIP/TEA buffer. The resulting solution has been extended to an overall ratio of 95:5 HFIP/TEA buffer against methanol (starting condition for RP-HPLC). DMTr-On purification was performed using condition 1. Obtained product fractions were combined and the solvent was evaporated in a vacuum concentrator. DMTr was cleaved by incubation with 1 mL of 80% acetic acid for 25 minutes at 25°C (500 rpm), following immediate solvent removal in a vacuum concentrator. DMtr-Off purification was performed using condition 2.

ON4

Alkyne-modified **ON4** was synthesized without DMTr. Remaining solvent from synthesis was removed under reduced pressure. Cleavage and deprotection was carried out in 28-30% ammonia solution (EMSURE®, *Sigma-Aldrich*) for 24 hours at 20°C. The solid phase was filtrated off via centrifugal filters (*VWR*) and washed with RNase-free water. The solvent was evaporated in a vacuum concentrator. Purification via RP-HPLC was performed using condition 3.

4. CuAAC Oligonucleotide Cyclization

CuAAC reaction

Cyclization of **ON4** was maintained by Copper(I)-catalyzed azide-alkyne cycloaddition (CuAAC) with bis(azidomethyl)benzene (AMB-linker). The reactions were implemented at 2.5 nmol scale. Cu(I) solutions (100 mM in DMSO) were freshly prepared from CuI. AMB-linker and tris[(1-benzyl-*1H*-1,2,3-triazol-4-yl)methyl]amine (TBTA) were stored in stock solutions at −20 °C and warmed up at room temperature before usage. Stock concentrations and also final concentrations for each reaction compound are given in Table S5 below.

2.5 nmol of **ON4** was dried before (vacuum concentrator) and diluted with RNase-free water resulting in a 2.00 mM solution. Afterwards a solution of AMB-linker in H_2O/DMSO/*t*BuOH 4:3:1 (10 mM) was added by pipetting straight into the solution. DMSO was added to a freshly dosed amount of CuI and the suspension was vortexed for 2 seconds. The remaining solid was centrifuged and the supernatant was used to prepare a mixture of CuI and TBTA in a 1:2 ratio. The CuI-TBTA solution was pipetted into the reaction solution, which was filled up finally with DMSO. The final oligonucleotide concentration was 769 µM. The Eppendorf reaction tube was fluted with argon and shaken at 500 U min^{-1} for two hours. The reaction temperature was one of two conditions, which was varied. The other one was the amount of AMB-linker.

Table S5. Stock concentrations and respective solvents of all reagent solutions, their final concentrations and utilized volumes for a 2.5 nmol scale reaction with 2 eq AMB-linker.

	c (stock)	solvent	c (final)	2.5 nmol scale
**ON4**	2.00 mM	H_2O	0.77 mM	1.25 µL
AMB-linker	10.0 mM	H_2O/DMSO/*t*BuOH 4:3:1	1.54 mM	0.50 µL
TBTA	100 mM	DMSO/*t*BuOH 3:1	15.4 mM	0.75 µL (1:2 pre-mixed Cu-TBTA solution)
CuI	100 mM	DMSO	7.69 mM	
			added DMSO	0.75 µL
			total volume	3.25 µL

A few conditions were tested in order to achieve the highest possible conversion rate of **ON4** into **cON4**. The resulting RP-HPLC chromatograms (254 nm) are shown in Figure S2.

RP-HPLC purification

The CuAAC-reaction was stopped by adding 45 µL HFIP/TEA buffer. Purification via RP-HPLC was performed using condition 5 (chapter 0). The fractions of the single signals were collected, the solvent was removed in a vacuum concentrator and the oligonucleotides were analyzed via LC-MS.

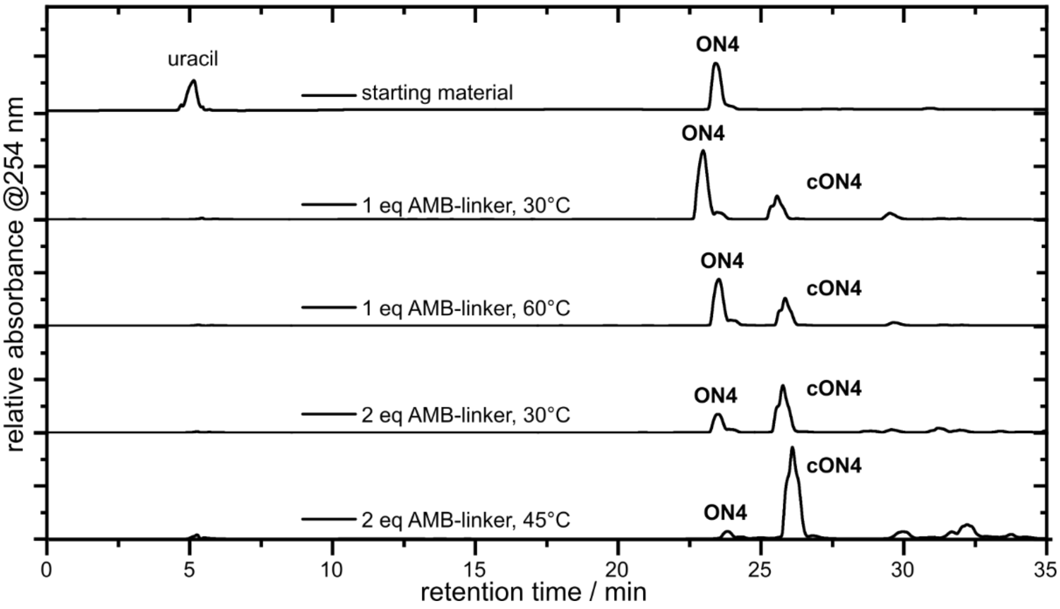

Figure S2. RP-HPLC chromatograms of starting material **ON4** and four different CuAAC reactions with different conditions.

The highest conversion was achieved at 45°C with two equivalents of AMB-linker. This condition was carried out several times in order to generate a sufficient quantity of **cON4**. Starting with 2.5 nmol **ON4** the average yield of this CuAAC reaction was 44%.

Proof of cyclization via LC-MS

Cyclization via CuAAC is only successful if both alkynes of **ON4** each react with an azide of one AMB linker. If this is not the case, ineffective by-products may be formed, which are shown in Figure S3. Possible adducts with one AMB-linker are **ON4a** and **ON4b**, which have the same mass as **cON4**, making differentiation by mass spectrometry impossible. The corresponding mass values are listed in Table S6. The cyclization can be confirmed, however, if the β-gal hydrolysis products are examined by mass spectrometry. After release of the β-Gal from by-products **ON4a** and **ON4b**, the linker would eliminate itself, leaving behind **ON4c** and **ON4d** with a free 5'-phosphate, which results in smaller masses. These masses were never found here.

cON4 theoretically has the possibility of still being cyclized after β-gal incubation if the self-immolative properties are lost through the CuAAC reaction. The structure of **cON4**–β-Gal would measure 5378 Da, which was also never found. Only in the case of a relinearized oligonucleotide is an water molecule added, so that the self-elimination process is quenched at the linker and also a free 5'-phosphate is built.

Table S6. Calculated oligonucleotide masses of possible (side-) products from CuAAC reaction of **ON4** with AMB-linker and masses of respective structures after incubation with β-gal.

	**oligonucleotide**	**calculated exact mass [Da]**
	**ON4**	5350.9804
**CuAAC products (+ AMB-linker)**	**ON4a**, **ON4b, cON4**	5539.0614
**β-gal hydrolization prodcuts**	**ON4c**	4999.8850
	**ON4d**	5187.9660
	**cON4** –β-Gal	5378.0164
	**linON4**	5395.0192

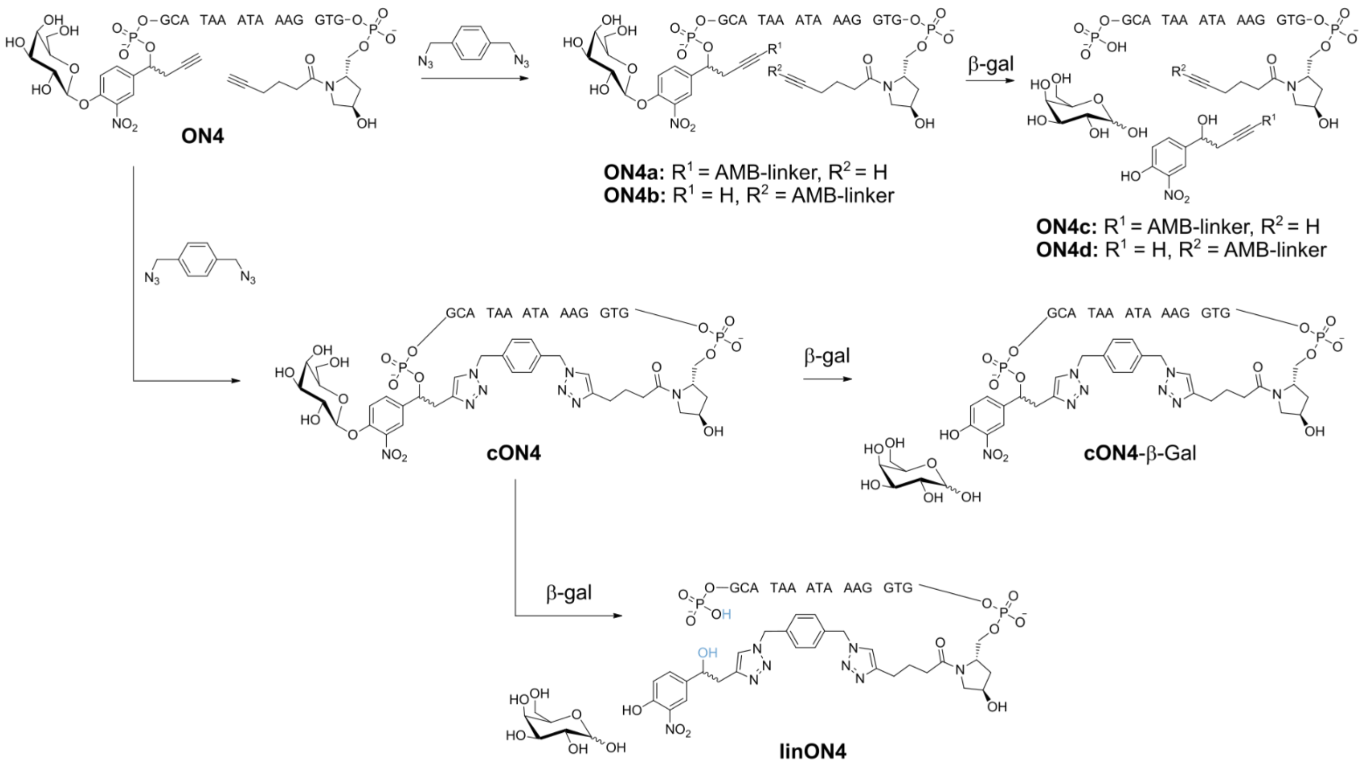

Figure S3. Cyclization of **ON4** via CuAAC with AMB-linker. Theoretical side-product formation of **ON4a** and **ON4b** (adducts) and their respective enzyme-hydrolyzed structures **ON4c** and **ON4d**. Formation of **cON4** arises from a productive cyclization. If the circle is still closed after enzymatic hydrolization, **cON4**-β-Gal is formed. A successful linearization ends in **linON4**, where specifically an additional H_2O (blue) is added.

5. Duplex Melting Temperature Measurement

Duplex melting temperatures were determined using an UV-vis spectrometer "Evolution 300" (ThermoFisher Scientific) equipped with a peltier element (*ThermoFisher Scientific*). Each 1 nmol of sense DNA strand **ON1**, **ON2**, **ON3**, **ON4** and **cON4** and complementary antisense DNA strand **ON5** were added together in 1 mL of an overall 1x PBS-buffered (pH 7.4) solution (each oligonucleotide 1 µM). The absorption spectra were recorded in a 10.00 mm path length quartz glass cuvette (*Hellma Analytics*). One measurement is made up of the increase or decrease in the temperature from approximately 16-20 °C to 80 °C or reversed with a gradient of 1 °C min^{-1}. Several measurements (n = 7) were carried out for each construct, recording four times absorbance at 260 nm while decreasing and three times while increasing temperatures.

The measured absorption spectra at 260 nm are visualized in Figure S4 plotted by *OriginLab*. The resulting inflection point of each graph was determined. The average value of the inflection points gives the final melting point and the standard deviations give the related error.

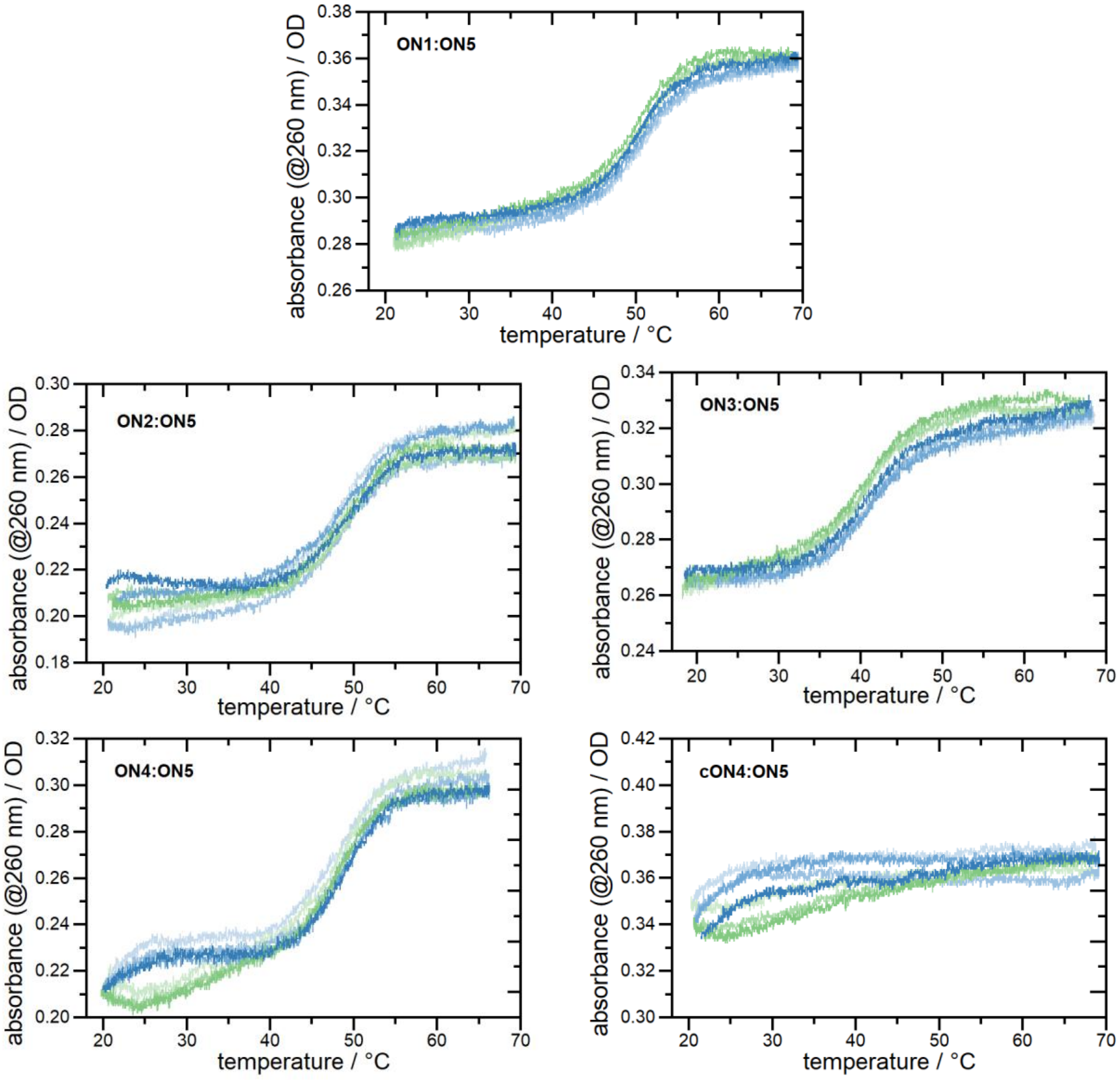

Figure S4. Melting temperature curves (primary data) of DNA-duplexes (each 1 nmol in 1 mL 1x PBS-buffer, n = 7).

6. Polyacrylamide Gel Electrophoresis (PAGE, native)

The duplex formation between **ON1**/**ON3**/**cON4** and **ON5** was also analyzed by polyacrylamide gel electrophoresis under native conditions. The gel was prepared from a 10 mL solution containing 0.57 mL glycerol (87% in water), 1 mL 10x Tris/Borate/Ethylendiaminetetraacetic acid (EDTA) (10x TBE-buffer), 4.68 mL water and 3.75 mL polyacrylamide solution (37.5:1, 40%) resulting in a 15% polyacrylamide solution. 0.5x TBE-buffer was used as elution buffer. Oligonucleotides (14 pmol) were incubated at 37 °C for one hour in 1x PBS-buffer (300 U min^{-1}) alone or together to form a duplex. Electrophoresis was performed at 120 V and 15 °C for two hours and 15 minutes.

Staining was done using SYBR Gold (*Thermo Fisher*) in six minutes. Gel imaging and documentation was performed via Gel Doc XR+ Gel Documentation System from *Bio-Rad*.

The resulting gel is shown in Figure S5. The results represents the melting point studies. One nucleobase-modification (**ON3**) doesn't prevent duplex formation, but the 3'- and 5'-cyclization completely prevents it.

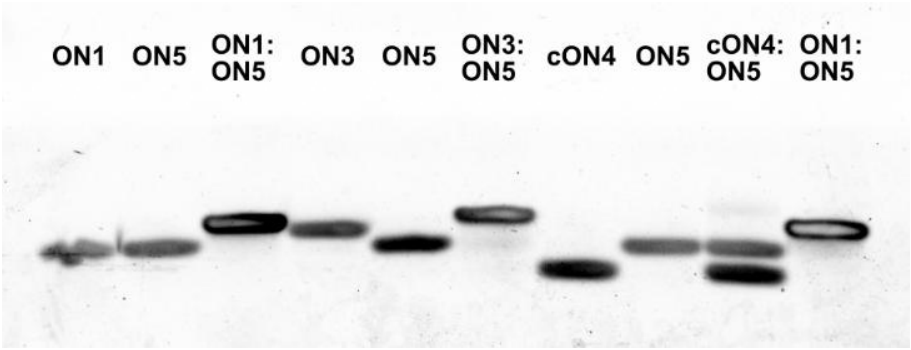

Figure S5. 15% native gel of oligonucleotides after incubation at 37 °C in 1x PBS-buffer for one hour. **ON1:ON5** and **ON3:ON5** clearly build a duplex, while **cON4** and **ON5** were separated.

7. Oligonucleotide Stability Tests

ON2, **ON3**, **ON4** and **cON4** were incubated in 1x PBS buffer for weeks at room temperature to investigate the stability of oligonucleotide modification β-Gal in solution. A 2.5 mM solution of 275 pmol oligonucleotide in 110 µL 1x PBS (pH 7.4) was left on the bench at room temperature. After one to four week one aliquot of 20 µL was removed and stored at -20°C. After four week, all aliquots were thawed and HFIP/TEA buffer containing uracil as standard, was added. The analysis was carried out using RP-HPLC (**ON2**/**ON3**: condition 4, **ON4**/**cON4**: condition 5). The chromatograms are shown in Figure S6. The modification of the nucleobase (**ON3**) as well as the 5'-frosting (**ON4**) and the corresponding cyclized product (**cON4**) are stable in 1x PBS buffer over 4 weeks. Unfortunately phosphate-frosted **ON2** showed an almost complete decomposition over four weeks (83% decomposition). The resulting signal shows the same retention time as **ON1**, which was also confirmed by mass spectrometry.

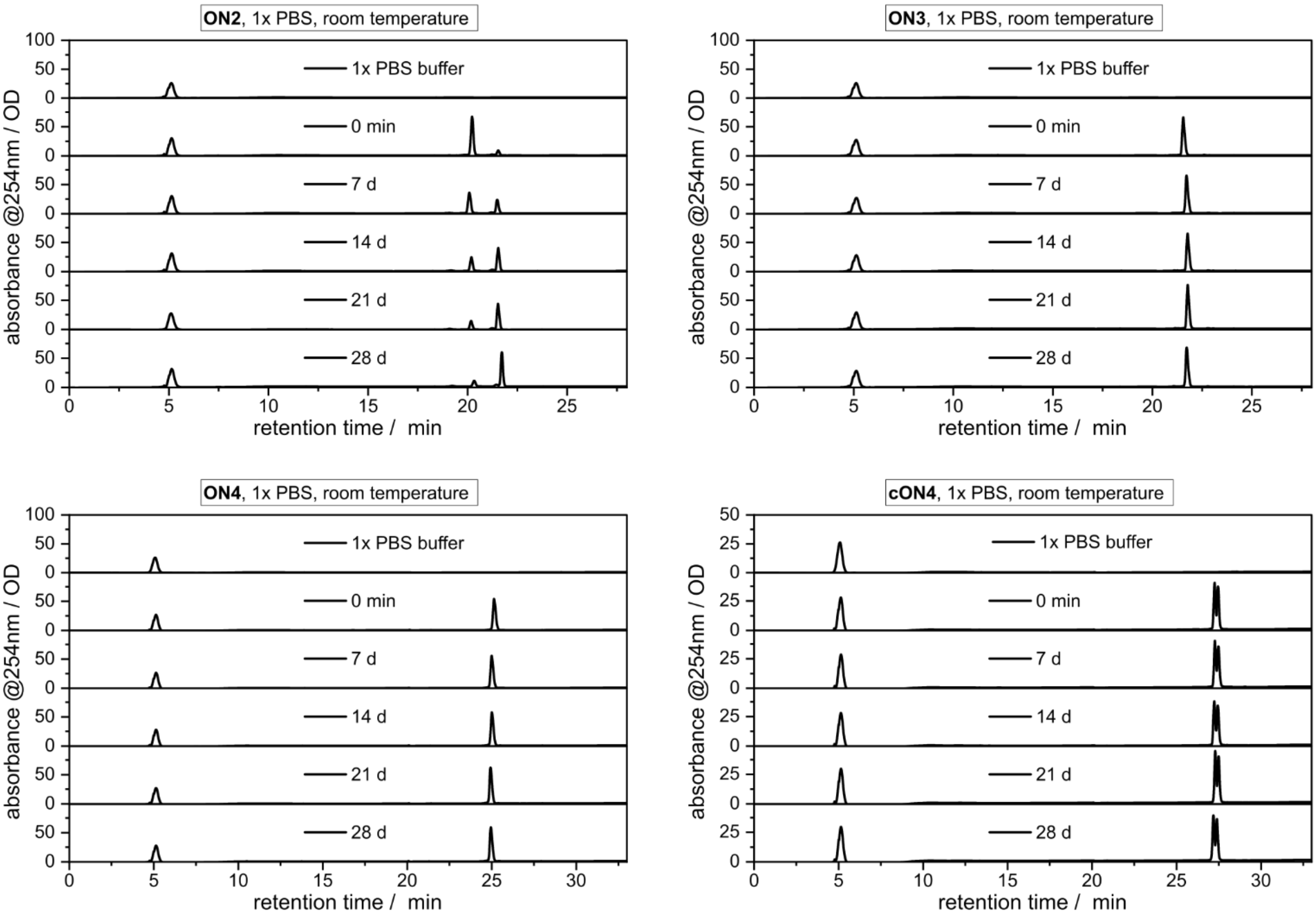

Figure S6. RP-HPLC chromatograms (254 nm) of stability experiments in 1x PBS buffer at room temperature. **ON2** and **ON3** were eluted using condition 4, **ON4** and **cON4** were eluted using condition 5. The signal having a retention time of 5 minutes is uracil as reference.

8. β-Galactosidase

β-gal (Aspergillus Oryzae)

β-Galactosidase (*Aspergillus oryzae*) was purchased from *Sigma Aldrich* (G5160, 25 ku, ≥8.0 units/mg solid, CAS: 9031-11-2). The specific activity was 24.7 u mg^{-1}. The assay was performed according to enzyme assay G5160 from Sigma Aldrich. Dry β-gal was delivered and stored at -20°C. Solutions were prepared immediately before use in cold (4°C) RNAse free water. A citrate-phosphate buffer (pH 4.5) was made from 100 mM citric acid and 200 mM sodium phosphate.

rhGLB1

Recombinant Human β-Galactosidase-1/GLB1 (rhGLB1) was purchased from *R&D Systems, a biotechne brand* (6464-GH-020). The specific activity was >1800 pmol min^{-1} μg^{-1}. rhGLB1 was supplied as a 0.44 mg mL^{-1} solution in 25 mM Tris and 150 mM NaCl (pH 7.5) and stored at -80°C. Aliquots of 100 ng in 10 µL Tris/NaCl buffer were freshly thawed before usage. Citrate-phosphate buffer (pH 3.5) was made from 100 mM citric acid and 200 mM sodium phosphate.

β-gal (Aspergillus Oryzae) assays

The assays were performed in pcr reaction tubes (*Eppendorf*). Each oligonucleotide was dissolved in citrate-phosphate buffer (pH 4.5) and heated to 37°C. β-gal solutions (1 µL) were added and the solution was resuspended. The reaction tubes were incubated for 30 minutes at 37°C in a thermomixer without moving. To stop the reaction, 25 µL of HFIP/triethylamine buffer containing uracil as internal standard was added. The reaction tube was strongly vortexed, centrifuged and incubated at 90°C for ten minutes while shaking (800 rpm). Analysis was performed using RP-HPLC (condition 4 or 5). Details are given in Table S7.

Table S7. β-gal (*Aspergillus Oryzae*) assay conditions.

	ON2	ON3	ON4	cON4
**oligonucleotide / pmol**	100	100	50	50
**buffer / µL**	5	5	5	5
**β-gal solution / µL**	1	1	1	1
**RP-HPLC condition**	4	4	5	5

The resulting chromatograms are shown in Figure 3a (**ON3**), Figure 4a (**cON4**), Figure S7 (**ON3**) and Figure S8 (**ON4**). To compare the retention times, starting material and released oligonucleotide were eluted without adding β-gal. **ON4**-β-Gal was isolated from solid phase synthesis (by product). The released oligonucleotides **ON3**-β-Gal, **ON4**-β-Gal and **linON4** were all confirmed by mass spectrometry (chapter 12). Isolated **ON3**-β-Gal is exact the same as **ON1**.

The results for phosphate-frosted **ON2** are shown in Figure S7. As already observed in the stability tests (chapter 7), **ON2** seems not to be completely stable in aqueous buffer solutions, that is why degradation was observed without adding β-gal. Nevertheless, the addition of the enzyme shows a complete removal, which means that the phosphate backbone modification has no negative steric hindrance on recognition by the enzyme.

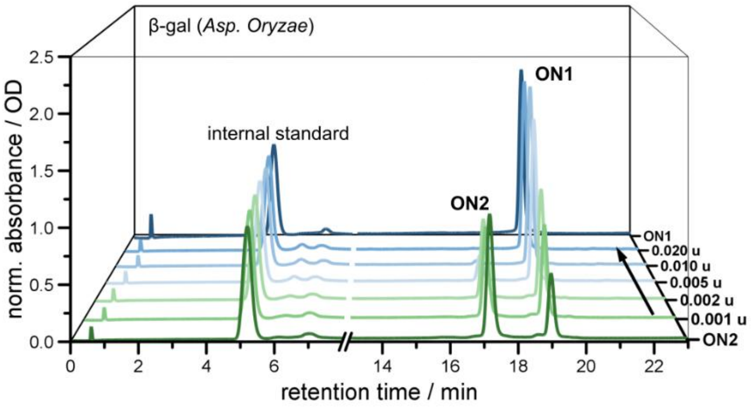

Figure S7. RP-HPLC chromatograms (254 nm) of phosphate-frosted **ON2** “defrosting” assays with β-gal (*Aspergillus oryzae*), citrate-phosphate-buffer (pH 4.5), 37 °C, 30 min.

Figure S8 shows the results for **ON4**. The alkyne modification doesn’t interfere with the enzyme recognition, and the linker continues his traceless self-immolation.

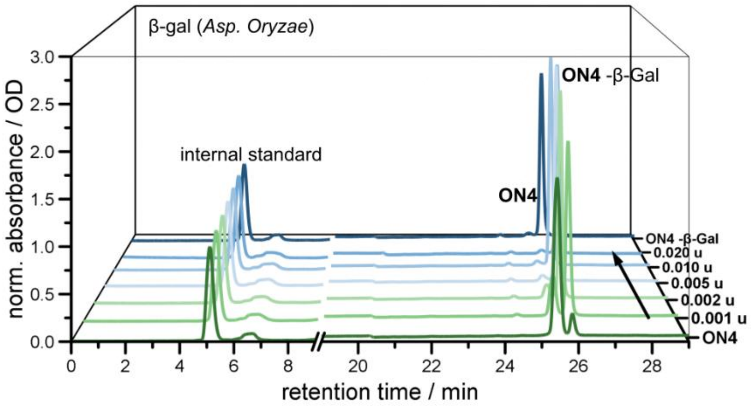

Figure S8. RP-HPLC chromatograms (254 nm) of 5’-frosted linear **ON4** “defrosting” assays with β-gal (*Aspergillus oryzae*), citrate-phosphate-buffer (pH 4.5), 37 °C, 30 min.

rhGLB1 assays

The assays were performed in pcr reaction tubes (Eppendorf). Each oligonucleotide was dissolved in citrate-phosphate buffer (pH 3.5) and heated to 37°C. β-gal solutions (1 µL) were added and the solution was pipetted up and down. The reaction tubes were incubated for 30 minutes at 37 °C in a thermomixer without moving. To stop the reaction, 25 µL of HFIP/triethylamine buffer containing uracil as internal standard was added. The reaction tube was strongly vortexed, centrifuged and incubated at 90 °C for ten minutes while shaking (800 rpm). Analysis was performed using RP-HPLC (condition 4 or 5). Details are given in Table S8.

Table S8. rhGLB1 assay conditions.

	ON3	ON4	cON4
**oligonucleotide / pmol**	100	50	50
**buffer / µL**	5	5	5
**β-gal solution / µL**	1	1	1
**RP-HPLC condition**	4	5	5

The resulting chromatograms are shown in Figure 3b (**ON3**), Figure 4b (**cON4**) and Figure S9 (**ON4**). To compare the retention times, starting material and released oligonucleotide were eluted without adding β-gal. **ON4**-β-Gal was isolated from solid phase synthesis (by product). The released oligonucleotides **ON3**-β-Gal, **ON4**-β-Gal and **linON4** were all confirmed by mass spectrometry.

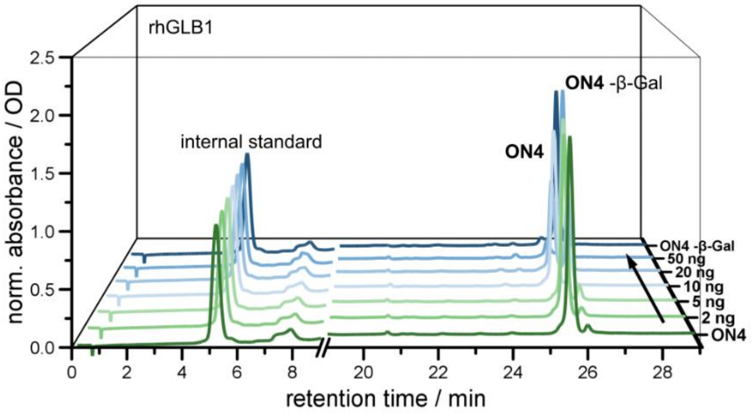

Figure S9. RP-HPLC chromatograms (254 nm) of 5'-frosted linear **ON4** "defrosting" assays with rhGLB1, citrate-phosphate-buffer (pH 3.5), 37 °C, 30 min.

9. β-Gal Overexpressing HEK293T Cell Lysate

Cell Culture

Lenti-X™ HEK293T cells (*Takara Bio*, 632180) were cultured in DMEM (*Thermo Fisher*, 21969-035) supplemented with 10% FCS (*Merck*, F7524), 2 mM L-Glutamine (*Thermo Fisher*, 25030081), 100 U/mL Penicillin and 100 U/mL Streptomycin (both *Thermo Fisher*, 15070063) at 37 °C, 5% CO2 and a relative humidity of 95%. Cells were passaged three times per week through detachment with Trypsin-EDTA (*Thermo Fisher*, 25200056).

Establishment of a β-gal Overexpressing Cellular System

Lentivirus was produced with the vector pLenti-hGLB1-cMyc-DDK-P2A-Puro (*Origene*, RC216106L3) as described previously.[1] Lenti-X™ HEK293T cells were transduced using thawed virus supernatant with a multiplicity of infection (MOI) of 1. 72h post transduction, media was exchanged and 2 ug/mL Puromycin (*Thermo Fisher*, A1113803) added to select for transduced cells. After 48h, media was exchanged and cells stocked. We refer to the transduced cells as HEK293T-GLB1 and the corresponding wildtype as HEK293T-WT. Both cell lines were seeded and after 48 h during exponential growth phase, cells were harvested, counted and $3.5x10^6$ cells lysed using CelLytic™ M (*Merck*, C2978) according to the manufacturer's protocol, and Protease and Phosphatase Inhibitors (*Thermo Fisher*, A32959) added.

To determine transgene expression, capillary Western blot (Simple Western) of denatured HEK293T-GLB1 and -WT lysates was performed using antibodies against α-Tubulin (*Cell Signaling*, 2125S) and β-Gal (Cell Signaling, 27198S). In concordance with the manufacturer's specifications, β-gal was detected at 100 kDa and 60 kDa, and α-Tubulin at 50 kDa (Figure S10A-D). Thereby, the expression and increased abundance of β-gal in HEK293T-GLB1 compared to HEK293T-WT was proven.

Enzymatic Assay

The enzymatic β-gal activities of HEK293T-GLB1 and -WT lysates were quantified using a fluorescence-based β-gal activity assay over time according to the manufacturer´s instructions (*Abcam*, ab189815). Each lysate was 1:60 diluted with lysis buffer. Subsequently, 75 µL diluted lysate was added in duplicates to 25 µL of a 100 nM solution of the fluorogenic substrate 3-carboxyumbelliferyl-β-D-galactopyranoside in a 96-well black polystyrene microplate. The same procedure was applied to 1:60 diluted 20 uM reference standard. Additionally, two wells with 75 µL of native lysis buffer and 25 µL of the substrate solution served as blanks. The plate was analyzed in a fluorescence plate reader (Tecan Infinite M200 Pro) measuring emission at 460 nm upon excitation with 390 nm in kinetic mode every 10 min for four hours. The plate was incubated at 25°C and 2 s orbitally shaken before excitation. The measured blank RFUs of each time point were subtracted from the corresponding sample and standard RFUs.

This assay showed 3.2 to 5.5 times higher β-gal activity of HEK293T-GLB1 compared to -WT constantly for four hours (Figure S10E). This functionally demonstrated that increased abundance of the enzyme is accompanied by increased activity, characterizing the lysates as an appropriate cellular test system for frosted oligos.

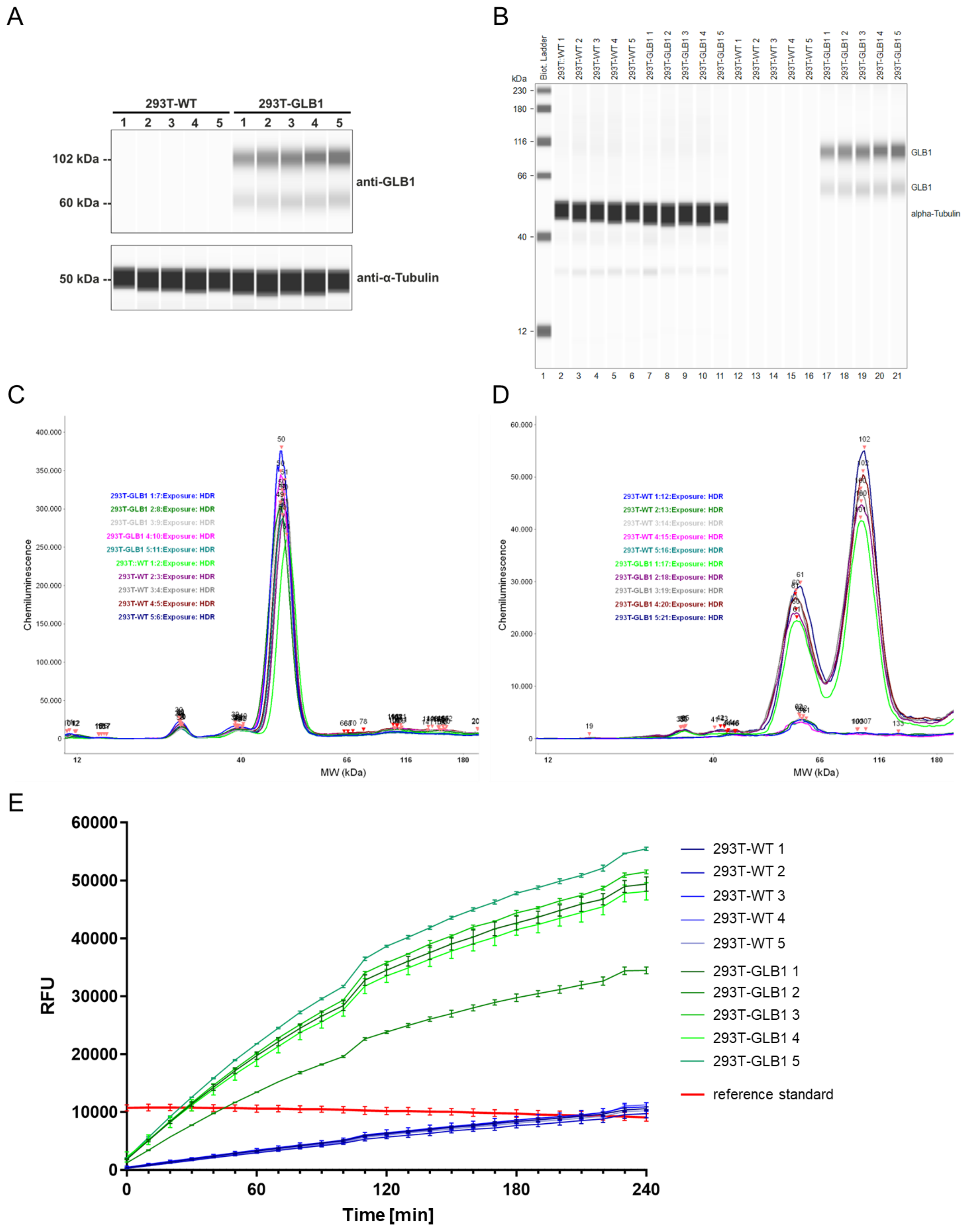

Figure S10. Establishment of a β-gal overexpressing cellular system. **A** Capillary Western Blot of HEK293T-GLB1 and HEK293T-WT with antibody detection of α-Tubulin and β-gal. **B** Complete representation of Capillary Western Blot bands. **C** α-Tubulin and **D** β-gal chemiluminescence signal overlays of Capillary Western Blot. **E** Fluorescence measurement of enzymatic β-gal activity over time. HDR High Dynamic Range. RFU Relative Fluorescence Units.

Oligonucleotide Assay

Cell lysate was stored at -20 °C and was freshly thawed when used. The lysate was inverted and carefully centrifuged. **ON3** and **cON4** were incubated with the cell lysate. Therefore, the cell lysate from HEK293T-wt and HEK293T-GLB1 was diluted 1:3 in citrate-phosphate buffer (pH 4.5). A solution of 550 pmol **ON3** or **cON4** in 22 µL 1x PBS buffer was prepared. An aliquot of 2 µL as starting reference was removed before cell lysate was added, remaining 500 pmol in 20 µL buffer. 60 µL of cell lysate in citrate-phosphate buffer was added to **ON3** or **cON4** in a pcr reaction tube and the solution was pipetted up and down slowly. The enzymatic reaction takes place at 37 °C in a thermomixer without moving. Aliquots of 8 µL were removed after 1.0 min, 2.5 min, 5.0 min, 7.5 min, 10 min, 15 min, 20 min, 25 min, 30 min and 60 min.

To stop the reaction, 25 µL of HFIP/triethylamine buffer containing uracil as internal standard was added directly after removing an aliquot. The reaction tube was strongly vortexed, centrifuged and incubated at 90 °C for ten minutes while shaking (950 rpm). Analysis was performed using RP-HPLC with condition 4 for **ON3** and condition 5 for **cON4**.

The chromatograms at 254 nm were analyzed and the signals of the internal standard uracil, starting material and product were integrated. Normalized to uracil, the values for enzymatic degradation were determined as shown in Figure 5. The error bars were calculated from the standard deviation of several independently performed assays (**ON3**: n = 3, **cON4**: n = 3).

10. NMR Spectra

[1]H Spectra of (2*R*,3*S*,4*S*,5*R*,6*S*)-2-(acetoxymethyl)-6-(4-formyl-2-nitrophenoxy)tetrahydro-2*H*-pyran-3,4,5-triyl triacetate (2)

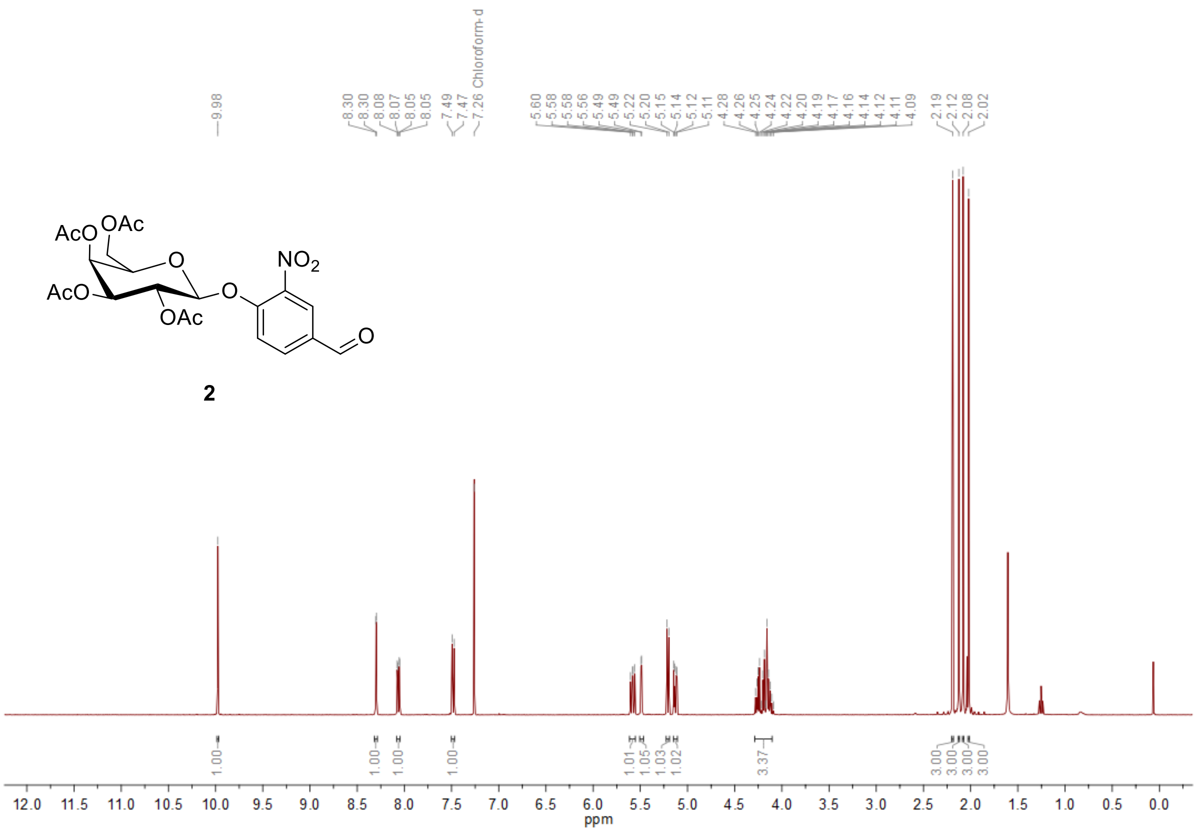

Figure S11. ^{1}H-NMR spectrum ($CDCl_3$) of **2**.

[1]H, [13]C Spectra of (2*R*,3*S*,4*S*,5*R*,6*S*)-2-(acetoxymethyl)-6-(4-(hydroxymethyl)-2-nitrophenoxy)tetrahydro-2*H*-pyran-3,4,5-triyl triacetate (3)

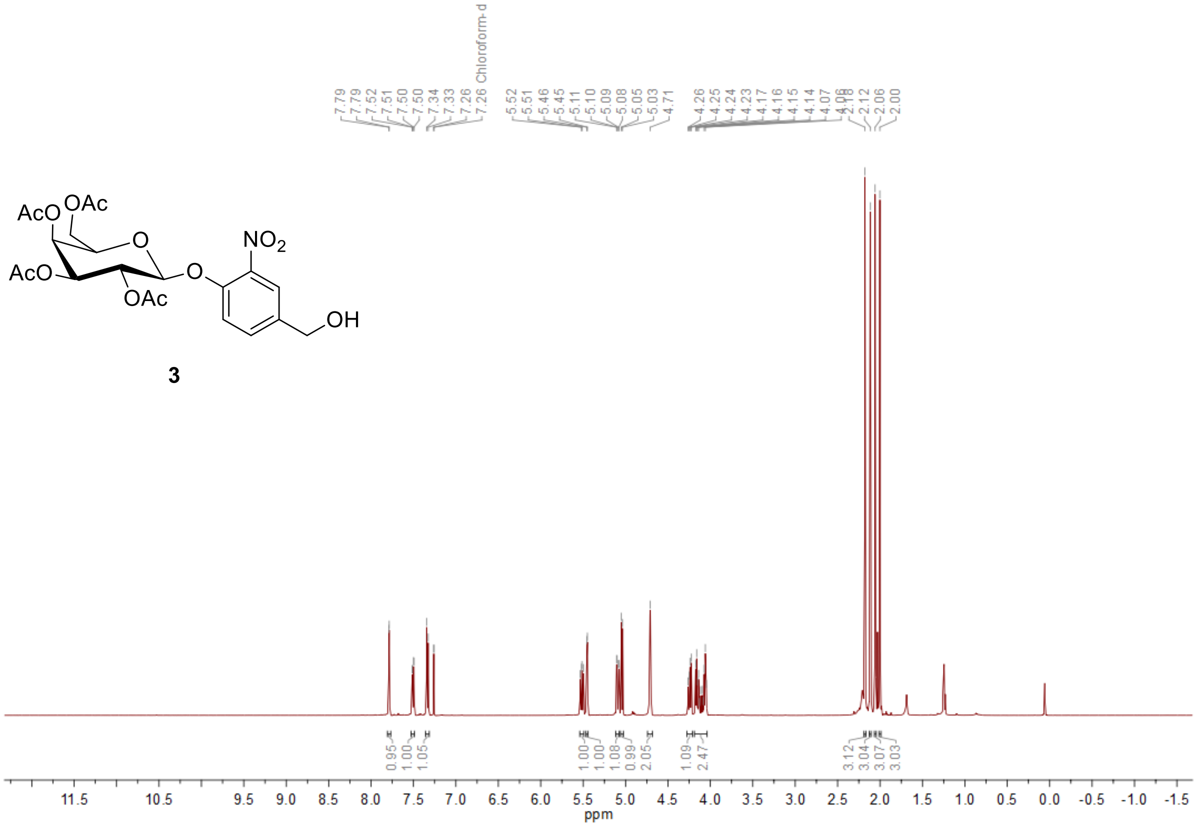

Figure S12. ^{1}H-NMR spectrum ($CDCl_3$) of **3**.

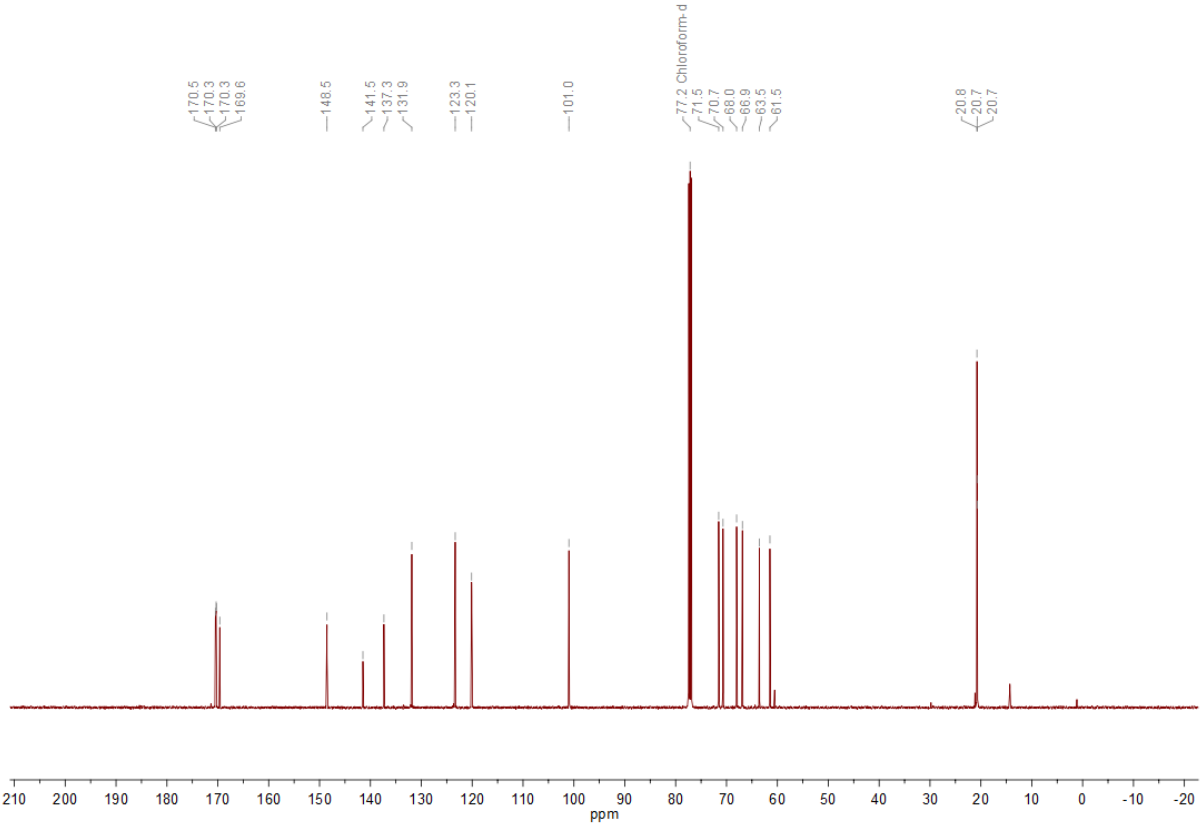

Figure S13. $^{13}C\{^1H\}$-NMR spectrum ($CDCl_3$) of **3**.

^{1}H, ^{13}C, ^{31}P Spectra of (2*R*,3*S*,4*S*,5*R*,6*S*)-2-(acetoxymethyl)-6-(4-(((bis(diisopropylamino)phosphino)oxy)methyl)-2-nitrophenoxy)tetrahydro-*2H*-pyran-3,4,5-triyl triacetate (4)

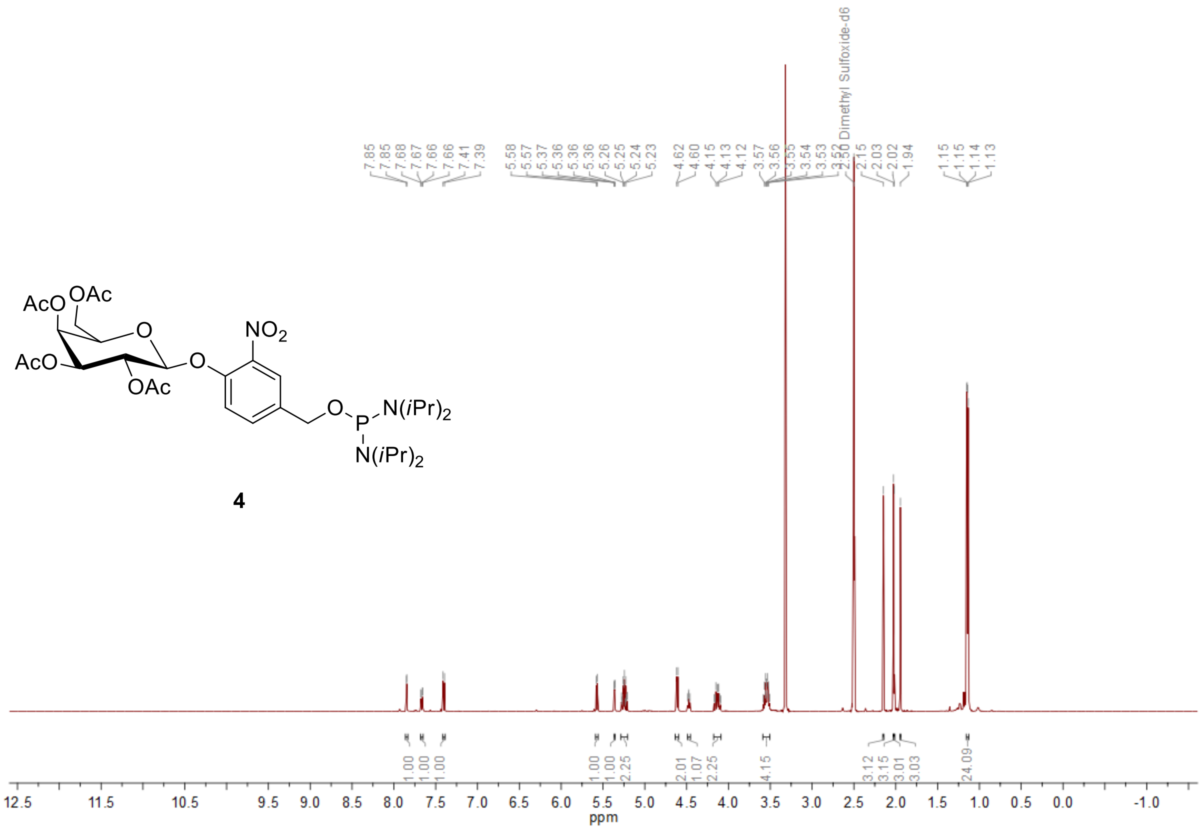

Figure S14. ^{1}H-NMR spectrum ($CDCl_3$) of **4**.

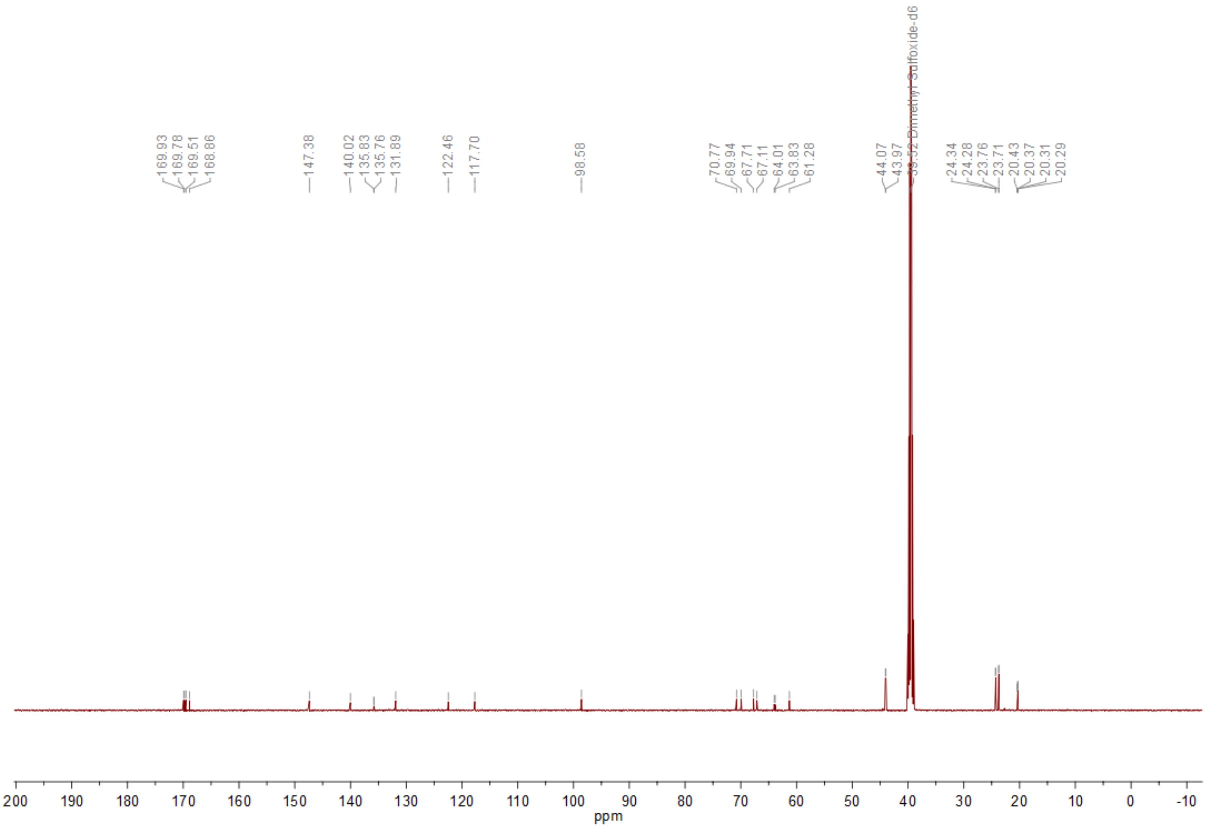

Figure S15. ^{13}C{^{1}H}-NMR spectrum ($CDCl_3$) of **4**.

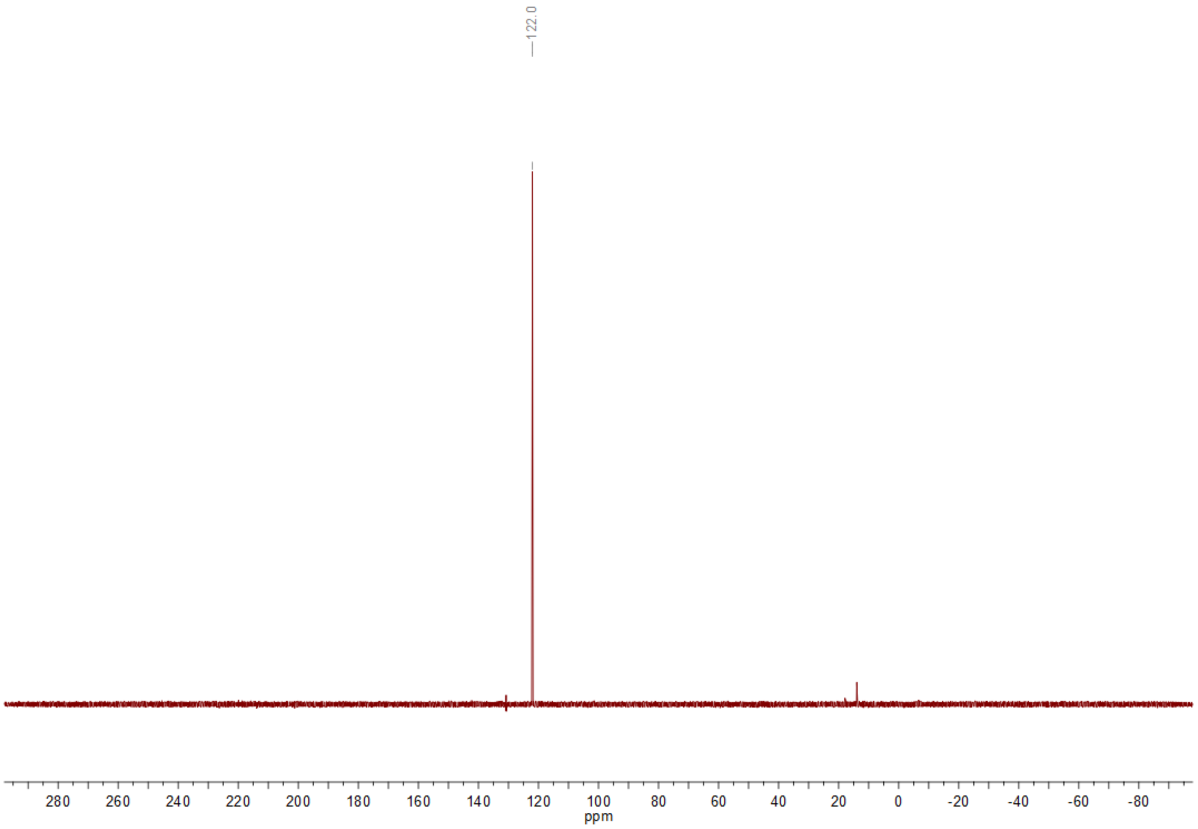

Figure S16. $^{31}P\{^{1}H\}$-NMR spectrum ($CDCl_3$) of **4**.

[1]H, ^{13}C, [31]P Spectra of (2*R*,3*S*,4*S*,5*R*,6*S*)-2-(acetoxymethyl)-6-(4-((((((2*R*,3*S*,5*R*)-2-((bis(4-methoxyphenyl) (phenyl)-methoxy)methyl)-5-(5-methyl-2,4-dioxo-3,4-dihydropyrimidin-1(2*H*)-yl)tetrahydrofuran-3-yl)oxy) (diiso-propylamino)-phosphino)oxy)methyl)-2-nitrophenoxy)tetrahydro-2*H*-pyran-3,4,5-triyl triacetate (5)

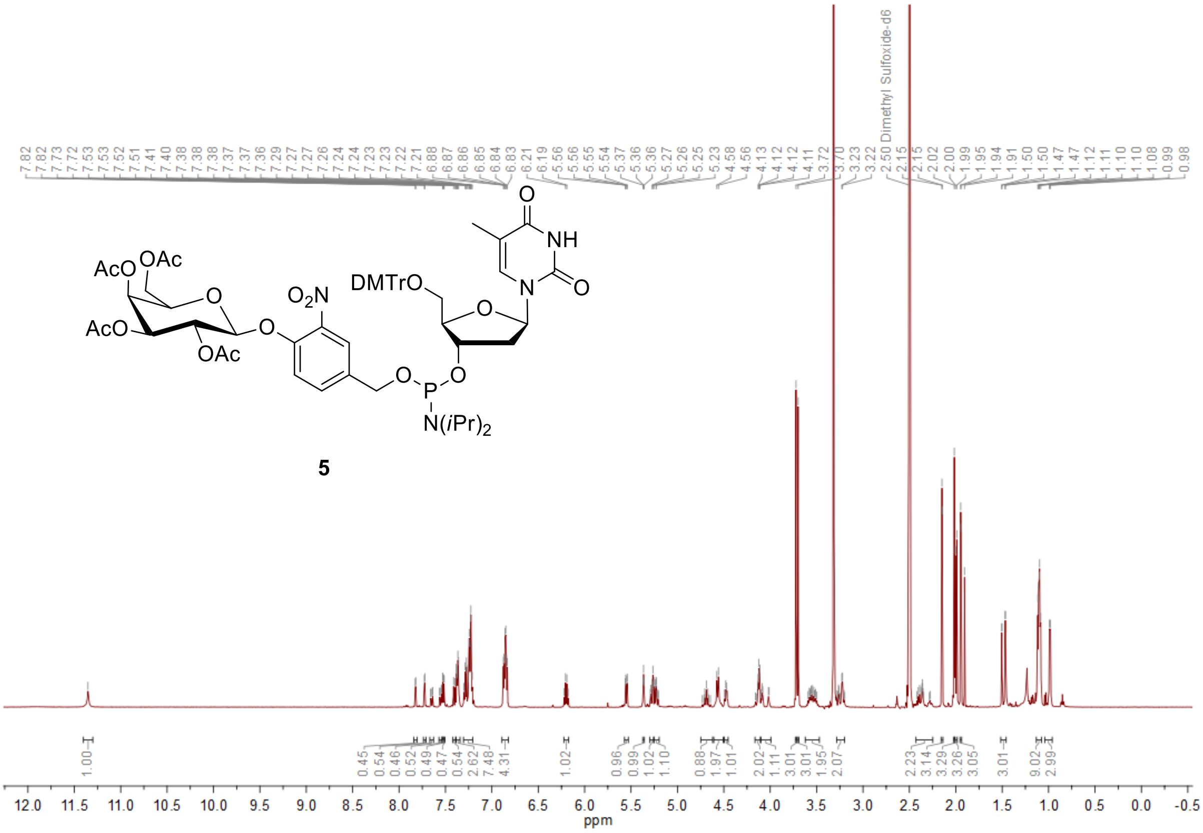

Figure S17. ^{1}H-NMR spectrum (DMSO-d_6) of **5**.

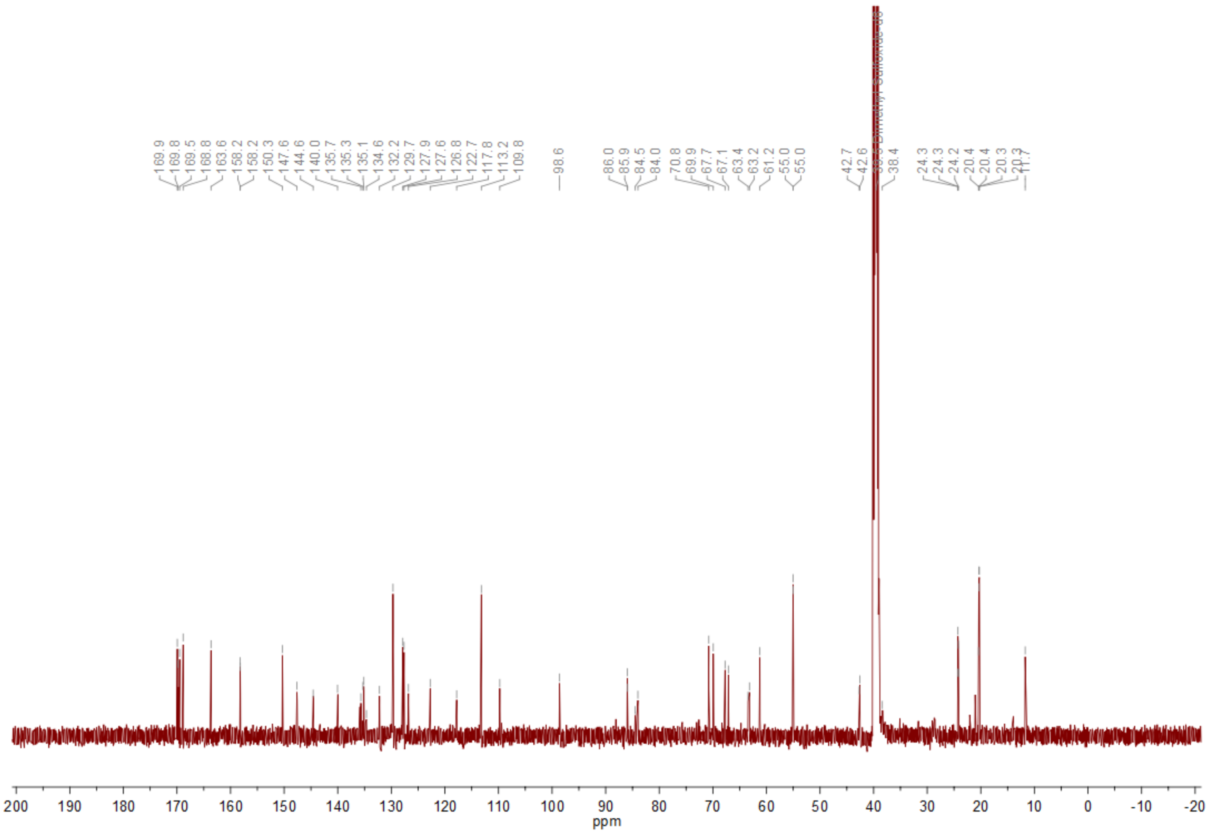

Figure S18. $^{13}C\{1H\}$-NMR spectrum (DMSO-d_6) of **5**.

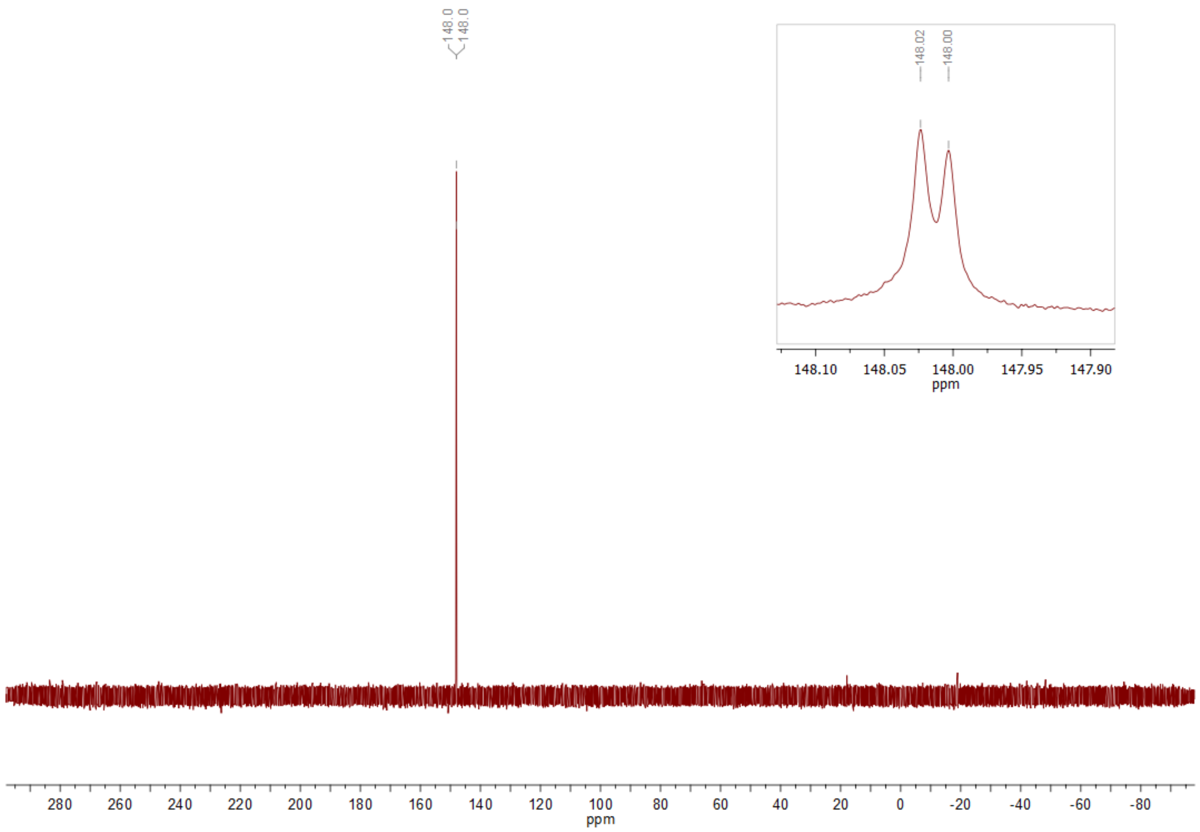

Figure S19. $^{31}P\{^{1}H\}$-NMR spectrum (DMSO-d_6) of **5**.

[1]H, [13]C Spectra of of (2*R*,3*S*,4*S*,5*R*,6*S*)-2-(acetoxymethyl)-6-(4-(((1-((2*R*,4*S*,5*R*)-4-((*tert*-butyldimethylsilyl)oxy)-5-(((*tert*-butyldimethylsilyl)oxy)methyl)tetrahydrofuran-2-yl)-5-methyl-2-oxo-1,2-dihydropyrimidin-4-yl)oxy)methyl)-2-nitrophenoxy)tetrahydro-2*H*-pyran-3,4,5-triyl triacetate (6)

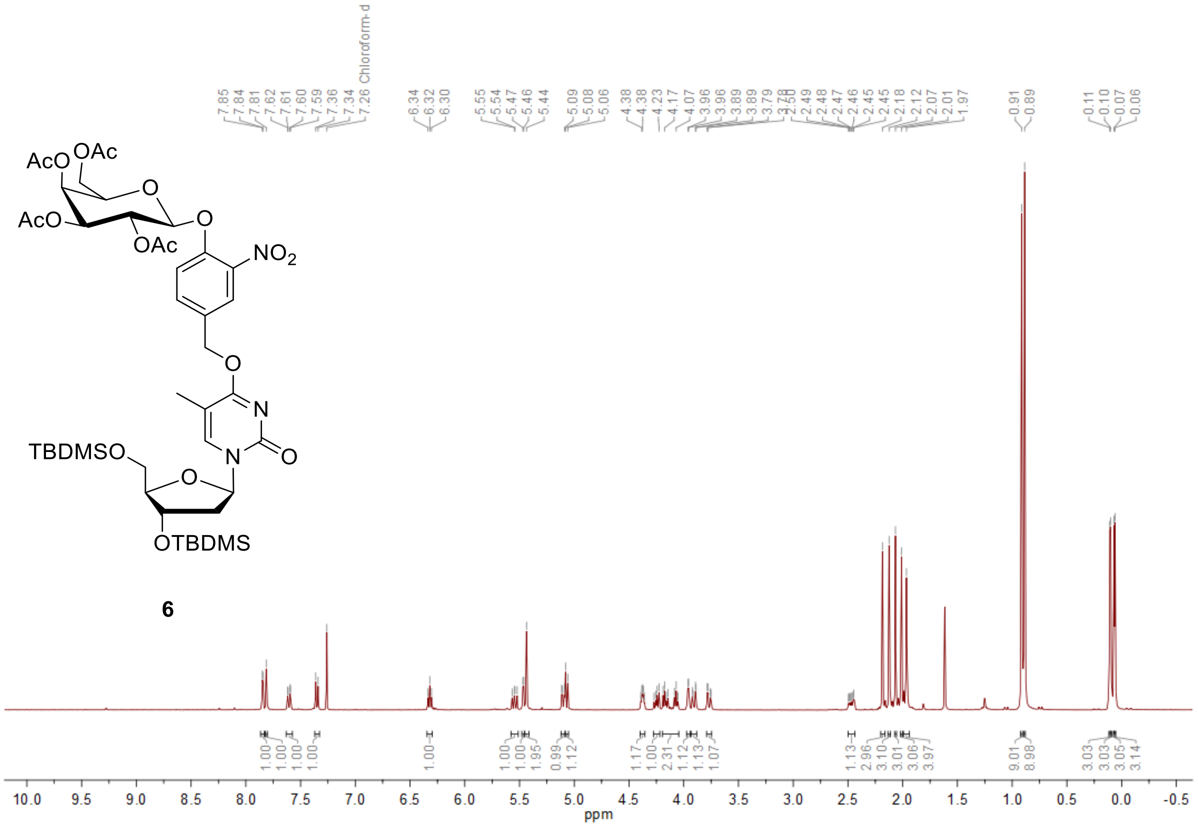

Figure S20. ^{1}H-NMR spectrum ($CDCl_3$) of **6**.

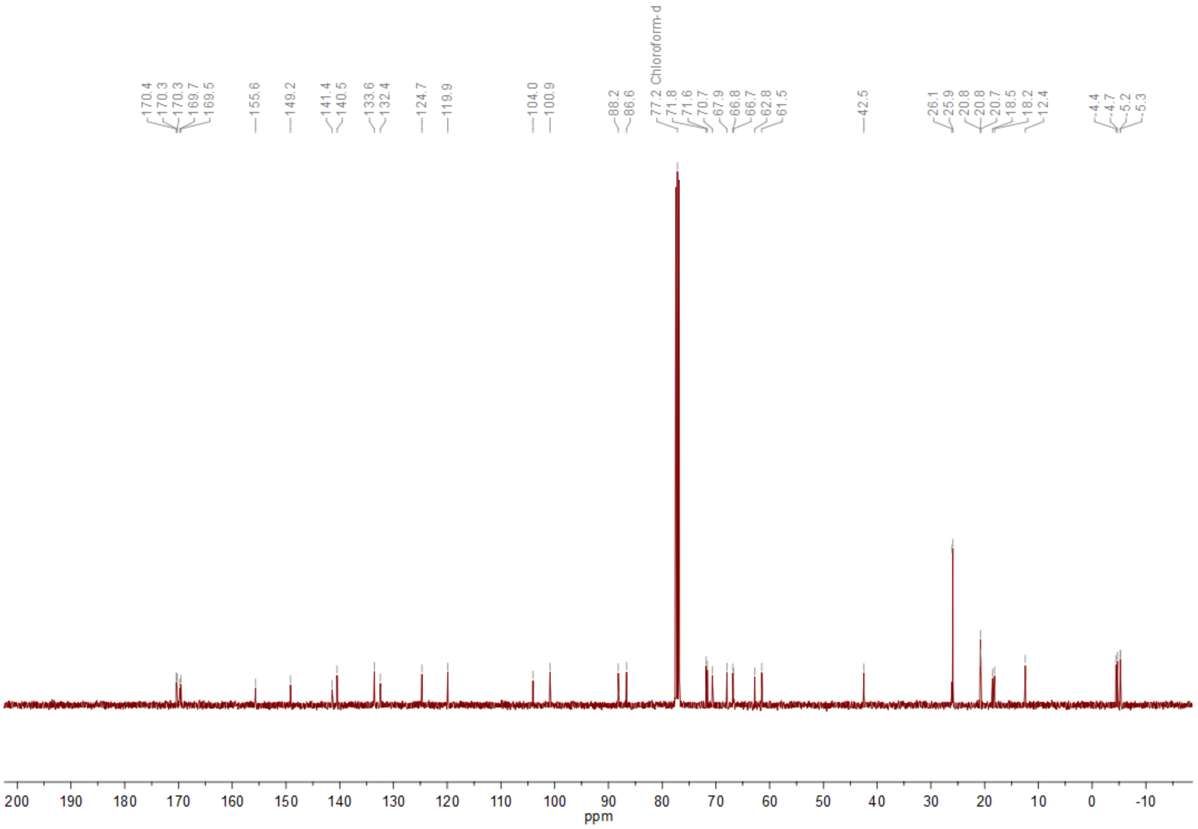

Figure S21. ^{13}C{^{1}H}-NMR spectrum ($CDCl_3$) of **6**.

^{1}H, ^{13}C Spectra of (2*R*,3*S*,4*S*,5*R*,6*S*)-2-(acetoxymethyl)-6-(4-(((1-((2*R*,4*S*,5*R*)-4-hydroxy-5-(hydroxymethyl)tetrahydro-furan-2-yl)-5-methyl-2-oxo-1,2-dihydropyrimidin-4-yl)oxy)methyl)-2-nitrophenoxy)tetrahydro-2*H*-pyran-3,4,5-triyl triacetate (7)

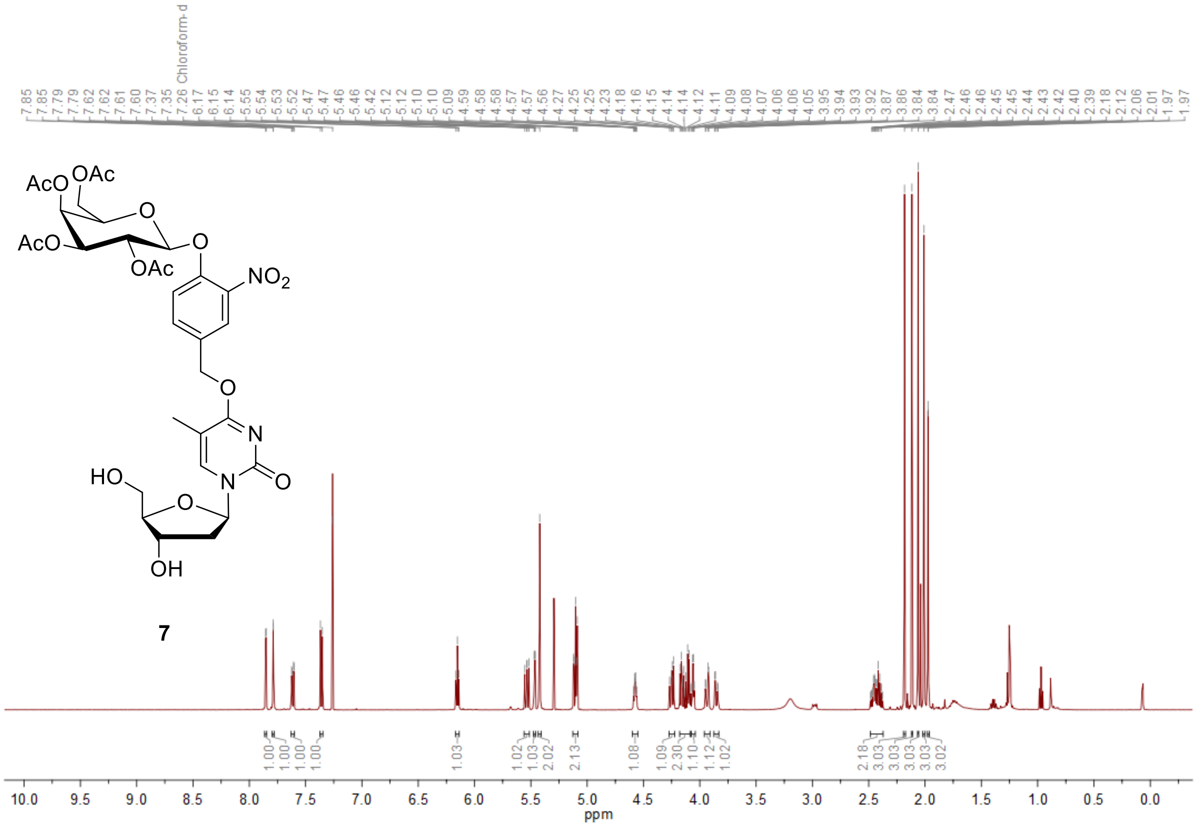

Figure S22. ^{1}H-NMR spectrum ($CDCl_3$) of **7**.

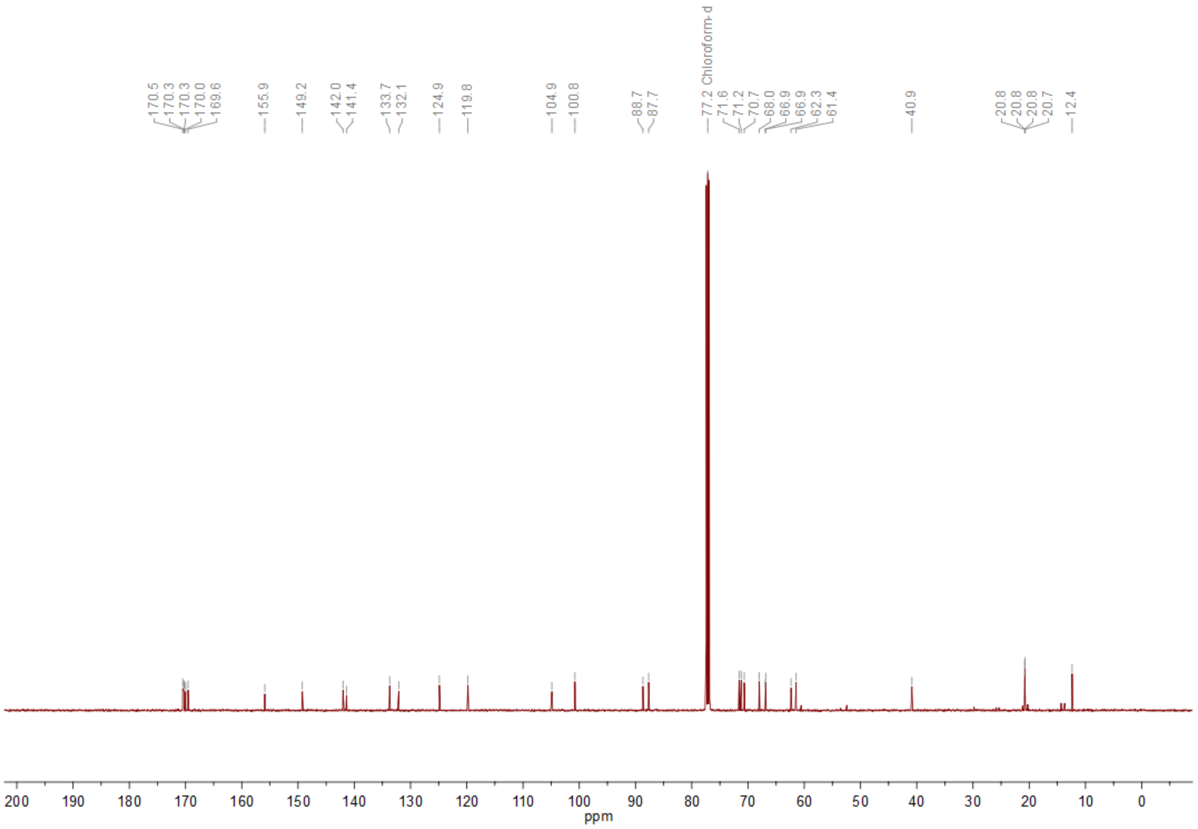

Figure S23. ^{13}C{^{1}H}-NMR spectrum ($CDCl_3$) of **7**.

^{1}H, ^{13}C Spectra of (2*R*,3*S*,4*S*,5*R*,6*S*)-2-(acetoxymethyl)-6-(4-(((1-((2*R*,4*S*,5*R*)-5-((bis(4-methoxyphenyl) (phenyl)methoxy) methyl)-4-hydroxytetrahydrofuran-2-yl)-5-methyl-2-oxo-1,2-dihydropyrimidin-4-yl)oxy) methyl)-2-nitrophenoxy)tetra-hydro-2*H*-pyran-3,4,5-triyl triacetate (8)

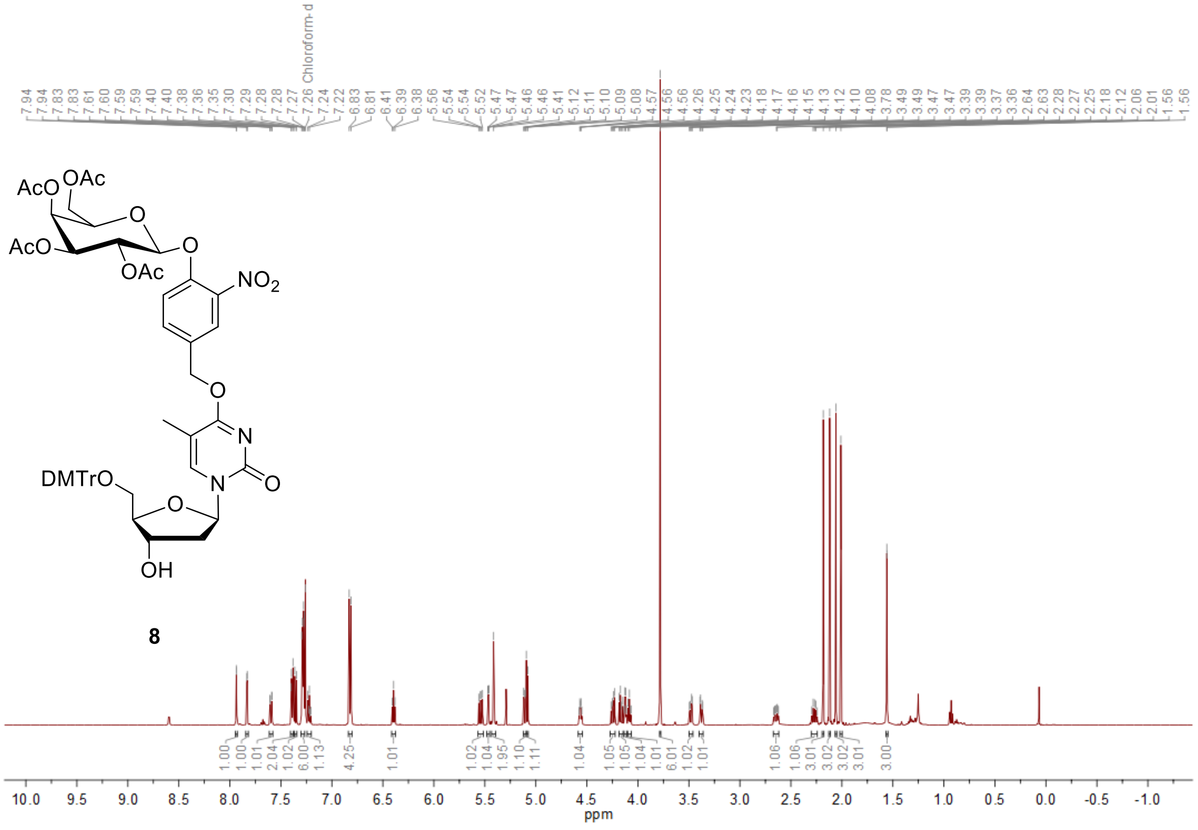

Figure S24. ^{1}H-NMR spectrum ($CDCl_3$) of **8**.

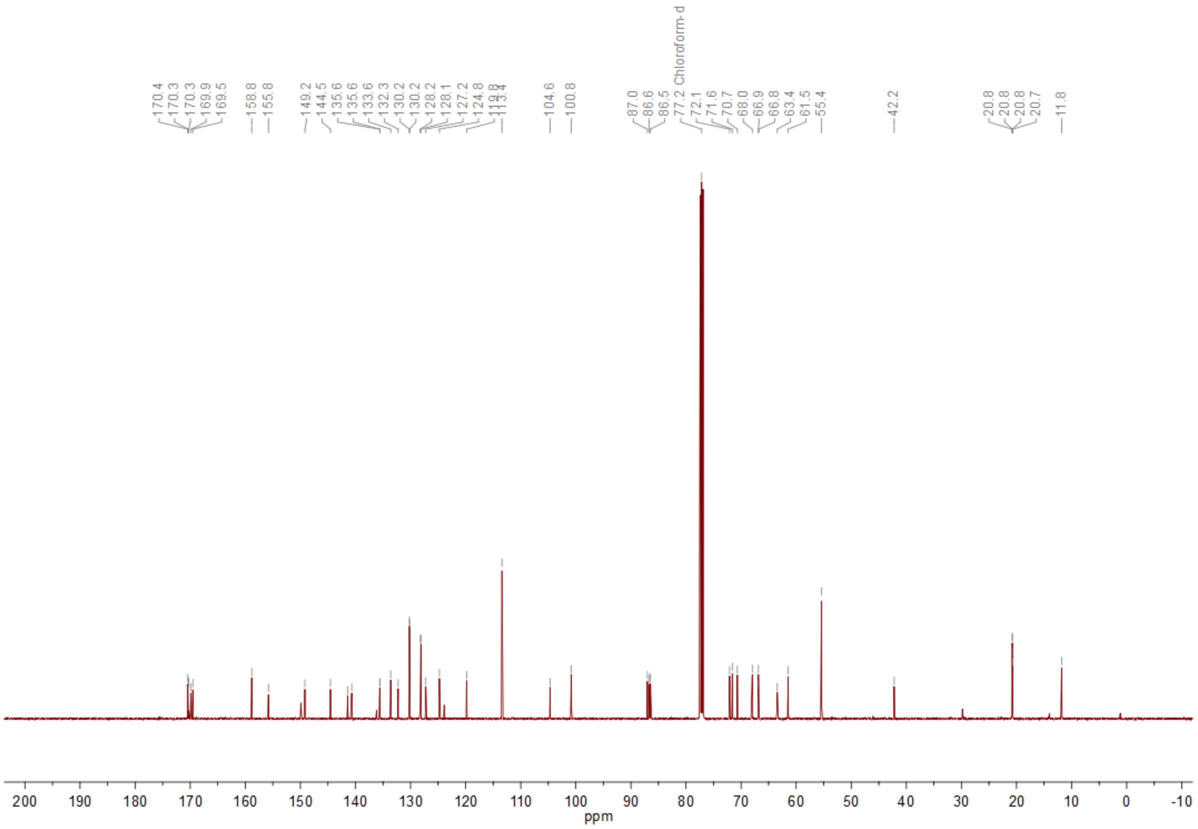

Figure S25. $^{13}C\{^{1}H\}$-NMR spectrum ($CDCl_3$) of **8**.

^{1}H, ^{13}C, ^{31}P Spectra of (2*R*,3*S*,4*S*,5*R*,6*S*)-2-(acetoxymethyl)-6-(4-(((1-((2*R*,4*S*,5*R*)-5-((bis(4-methoxyphenyl)(phenyl)methoxy)methyl)-4-(((2-cyanoethoxy)(diisopropylamino)phosphino)oxy) tetrahydrofuran-2-yl)-5-methyl-2-oxo-1,2-dihydropyrimidin-4-yl)oxy)methyl)-2-nitrophenoxy)tetrahydro-2*H*-pyran-3,4,5-triyl triacetate (9)

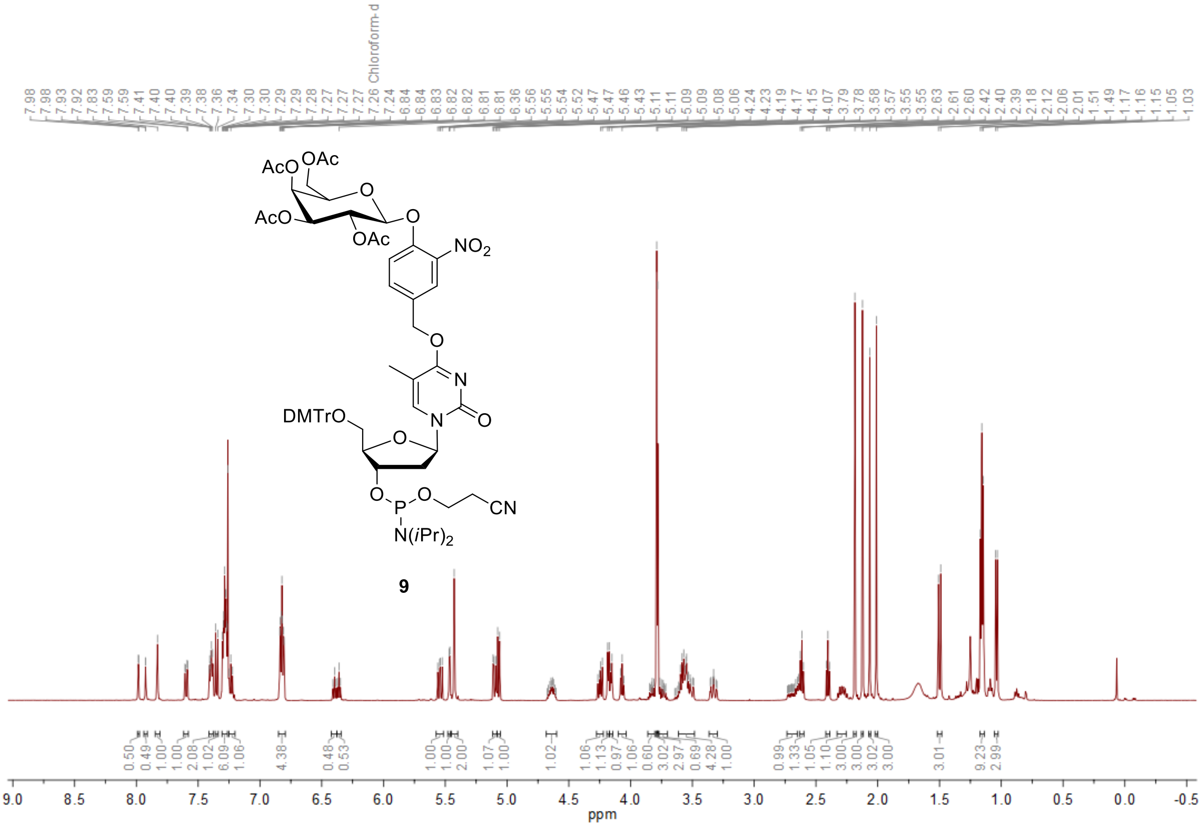

Figure S26. ^{1}H-NMR spectrum ($CDCl_3$) of **9**.

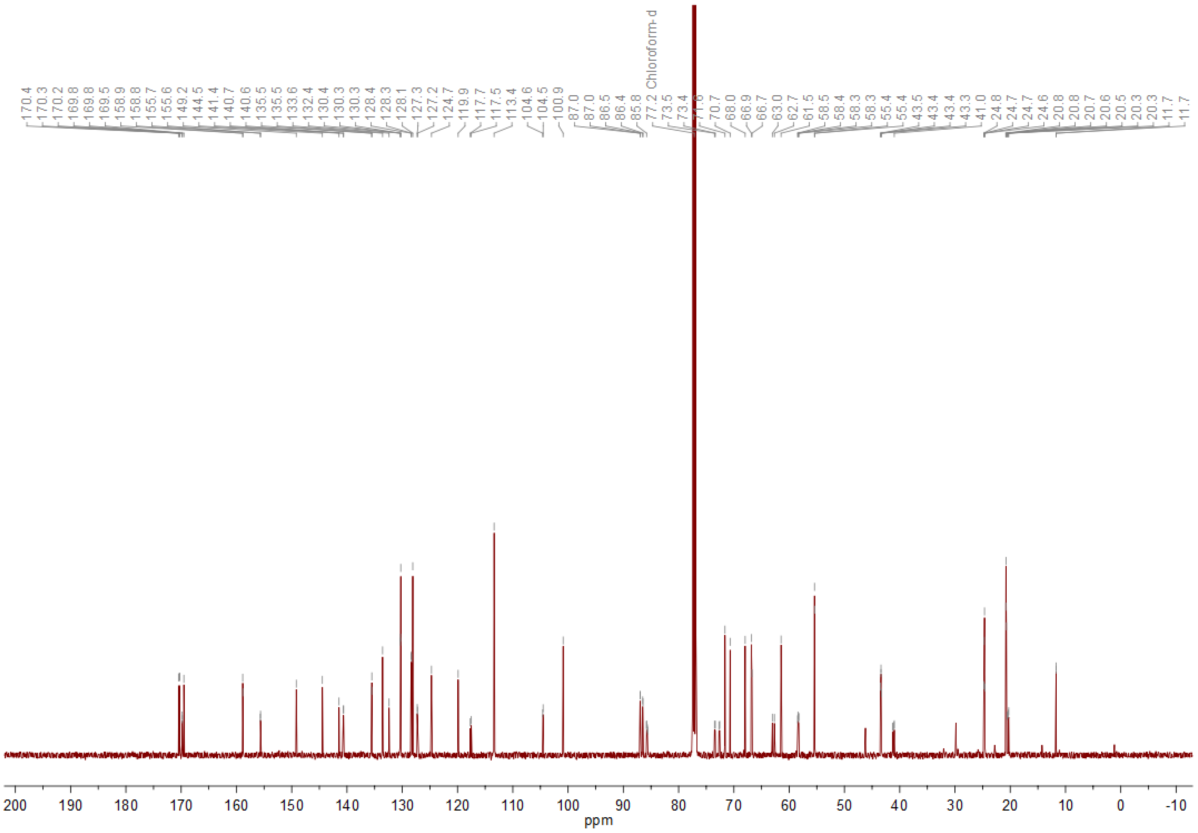

Figure S27. $^{13}C\{^1H\}$-NMR spectrum ($CDCl_3$) of **9**.

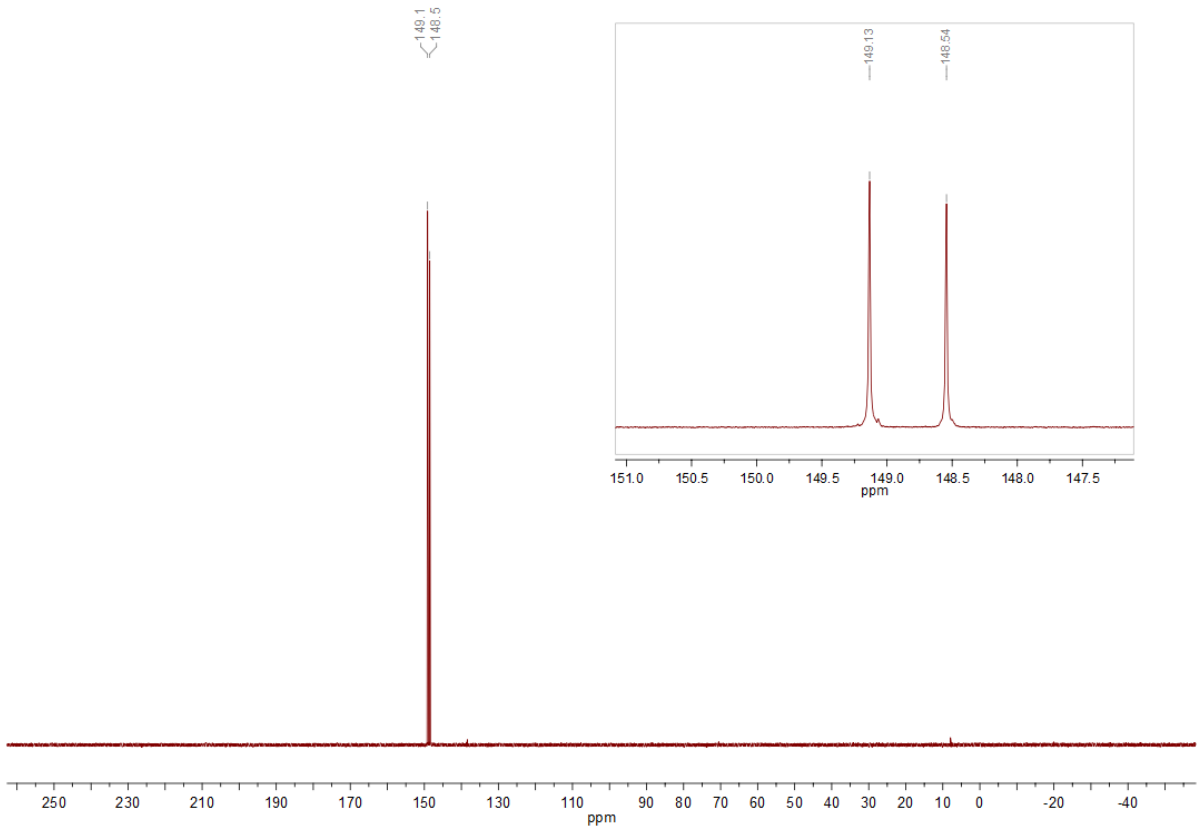

Figure S28. $^{31}P\{^1H\}$-NMR spectrum ($CDCl_3$) of **9**.

[1]H, [13]C Spectra of 4-(1-hydroxybut-3-yn-1-yl)-2-nitrophenol (10)

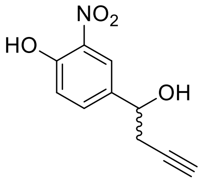

10

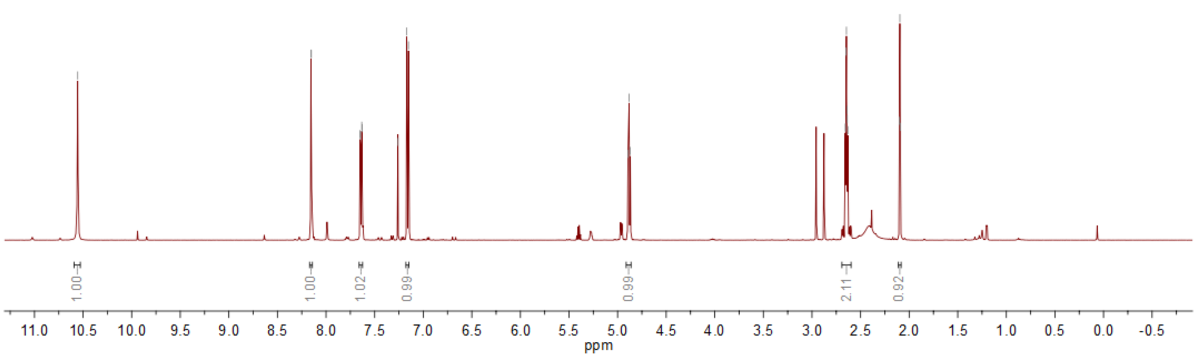

Figure S29. ^{1}H-NMR spectrum ($CDCl_3$) of **10**.

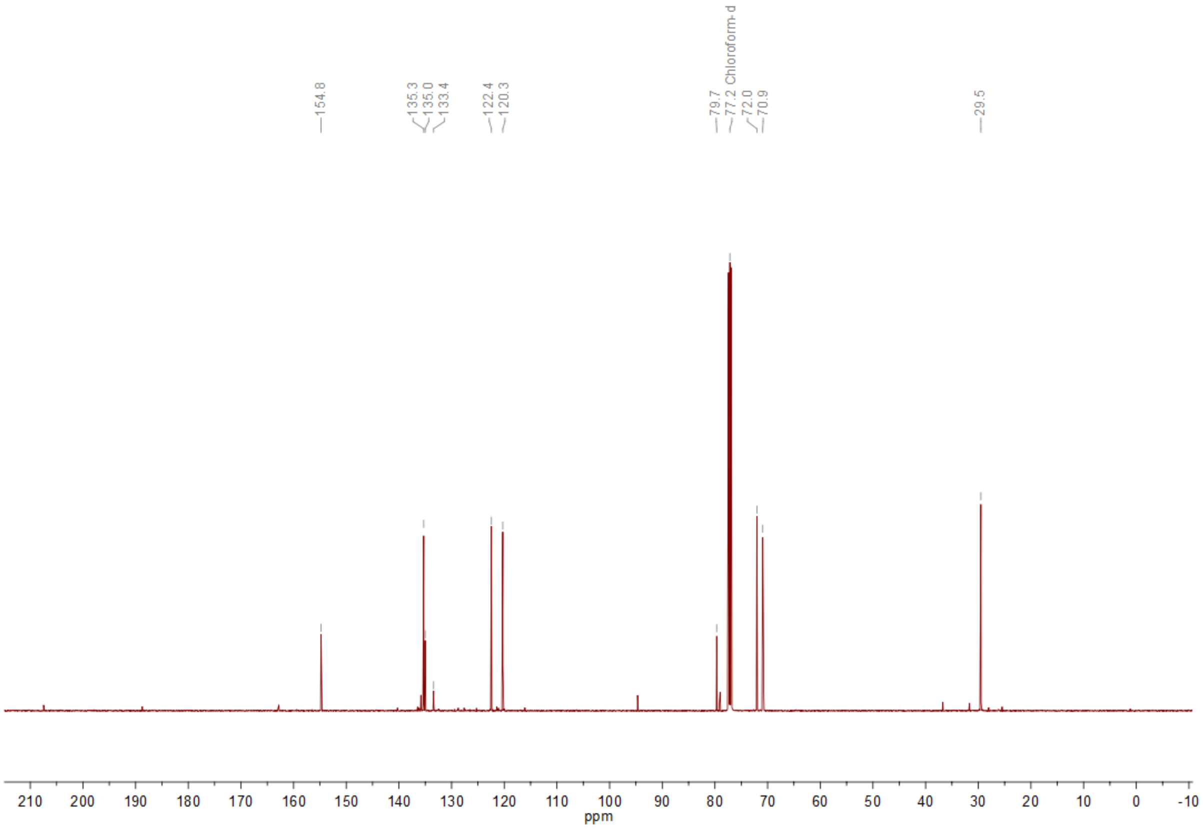

Figure S30. $^{13}C\{^{1}H\}$-NMR spectrum ($CDCl_3$) of **10**.

^{1}H, ^{13}C Spectra of (2*R*,3*S*,4*S*,5*R*,6*S*)-2-(acetoxymethyl)-6-(4-(1-hydroxybut-3-yn-1-yl)-2-nitrophenoxy)tetrahydro-2*H*-pyran-3,4,5-triyl triacetate (11)

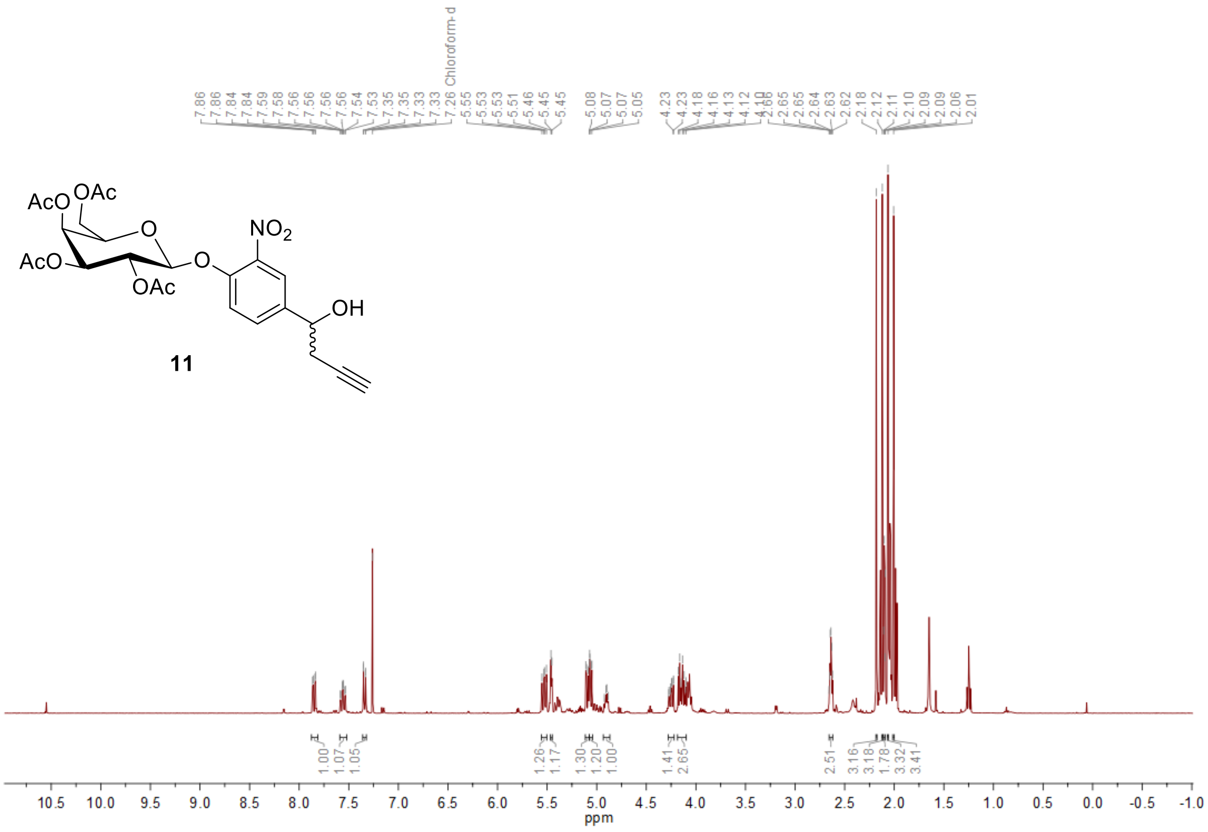

Figure S31. ^{1}H-NMR spectrum ($CDCl_3$) of **11**.

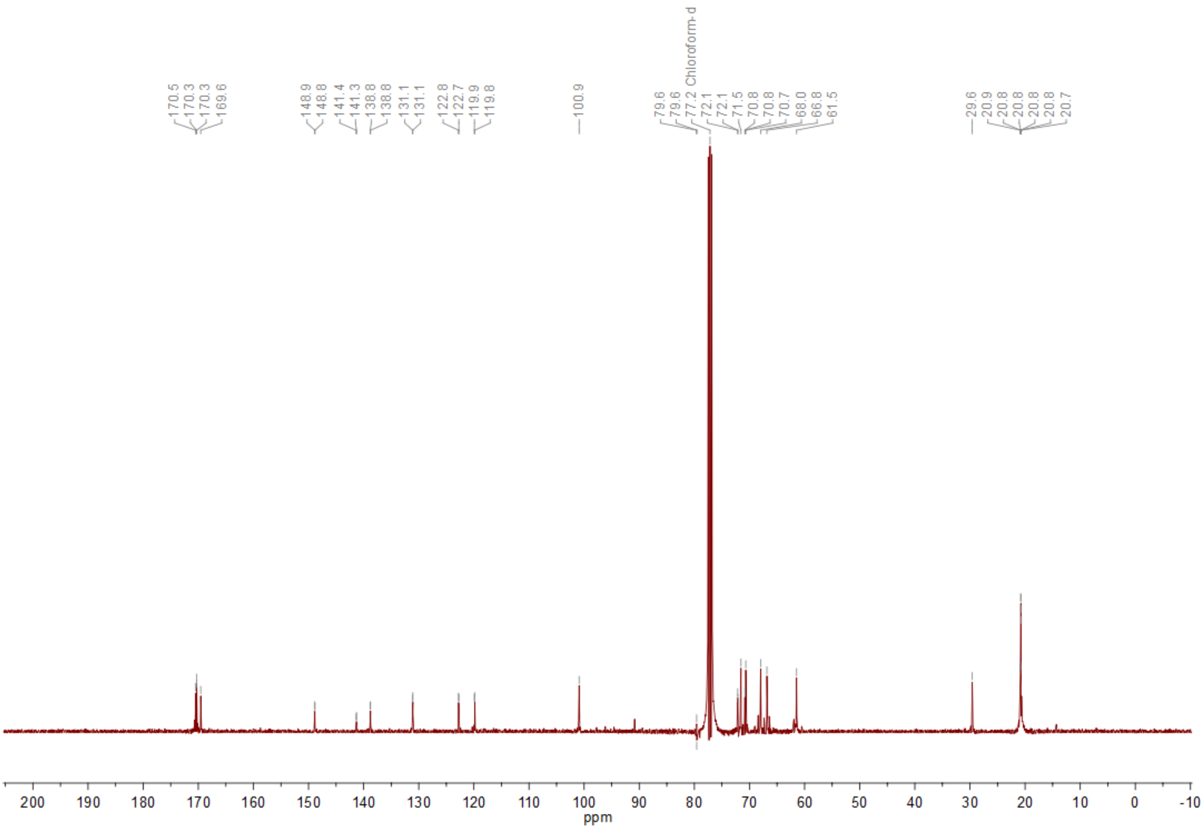

Figure S32. $^{13}C\{^{1}H\}$-NMR spectrum ($CDCl_3$) of **11**.

^{1}H, ^{13}C, ^{31}P Spectra of (2*R*,3*S*,4*S*,5*R*,6*S*)-2-(acetoxymethyl)-6-(4-(1-(((2-cyanoethoxy)(diisopropylamino)phosphino)-oxy)but-3-yn-1-yl)-2-nitrophenoxy)tetrahydro-*2H*-pyran-3,4,5-triyl triacetate (12)

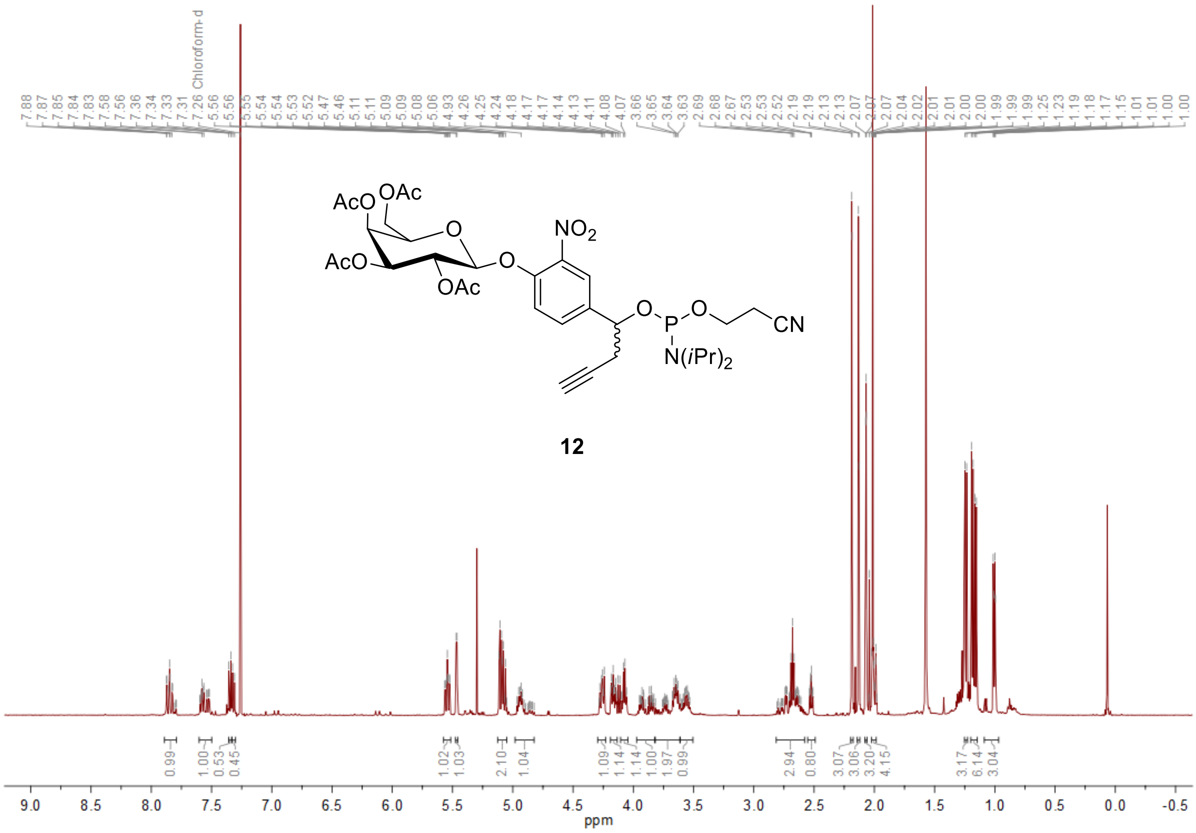

Figure S33. ^{1}H-NMR spectrum ($CDCl_3$) of **12**.

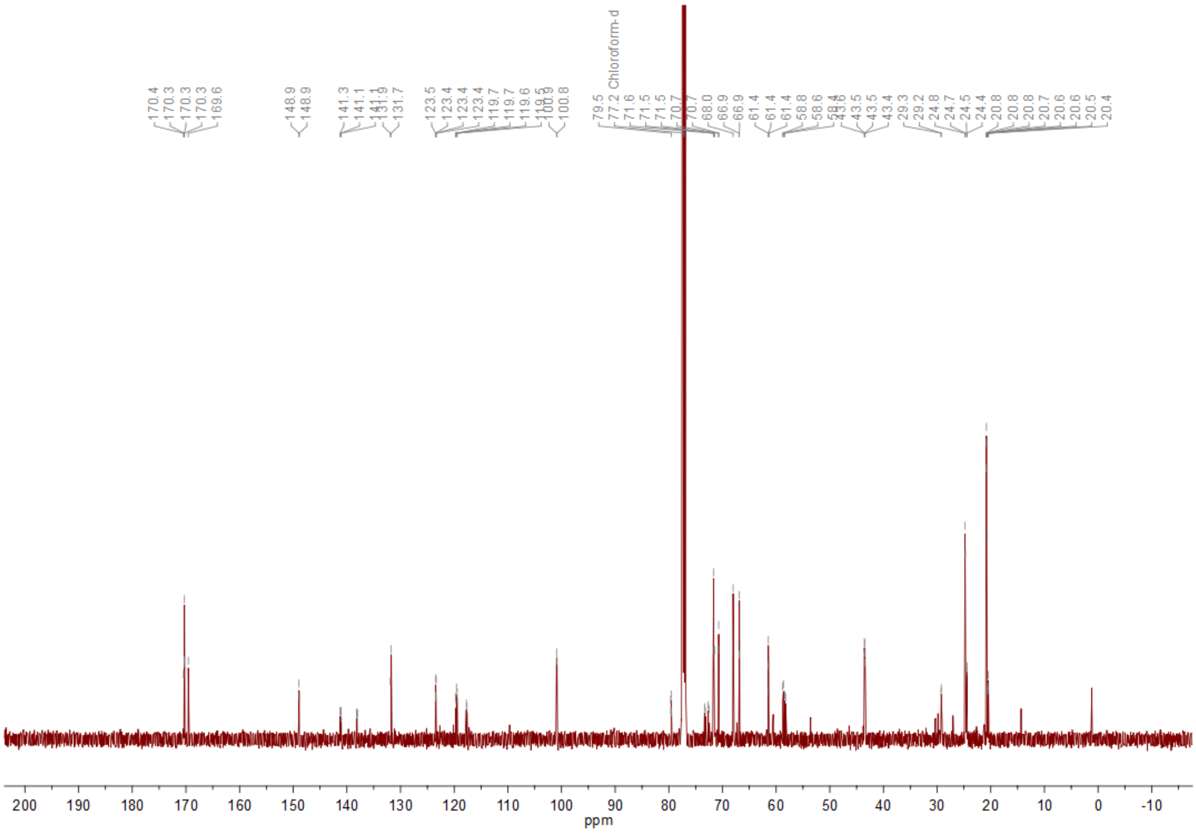

Figure S34. ^{13}C{^{1}H}-NMR spectrum ($CDCl_3$) of **12**.

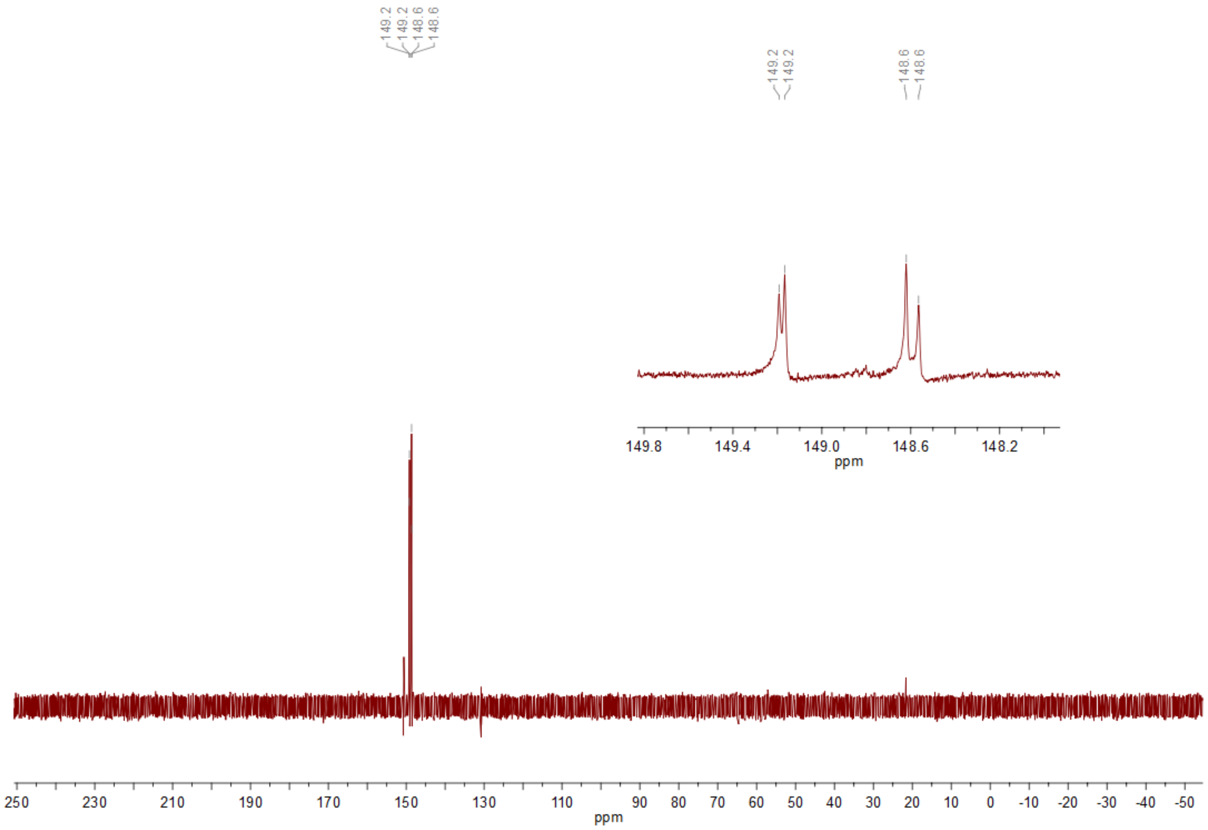

Figure S35. $^{31}P\{^{1}H\}$-NMR spectrum ($CDCl_3$) of **12**.

11. Mass Spectra (Small Molecules)

ESI-MS of (2*R*,3*S*,4*S*,5*R*,6*S*)-2-(acetoxymethyl)-6-(4-formyl-2-nitrophenoxy)tetrahydro-2*H*-pyran-3,4,5-triyl triacetate (2)

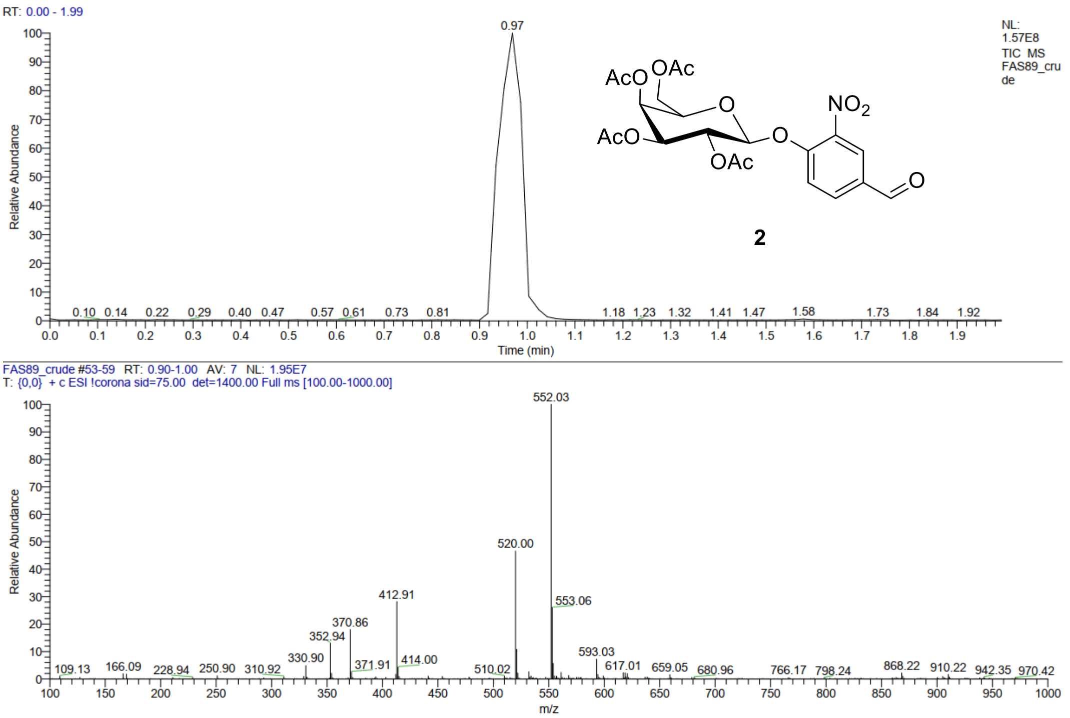

Figure S36. ESI-MS spectrum of **2**.

MALDI-MS of (2*R*,3*S*,4*S*,5*R*,6*S*)-2-(acetoxymethyl)-6-(4-(hydroxymethyl)-2-nitrophenoxy)tetrahydro-2*H*-pyran-3,4,5-triyl triacetate (3)

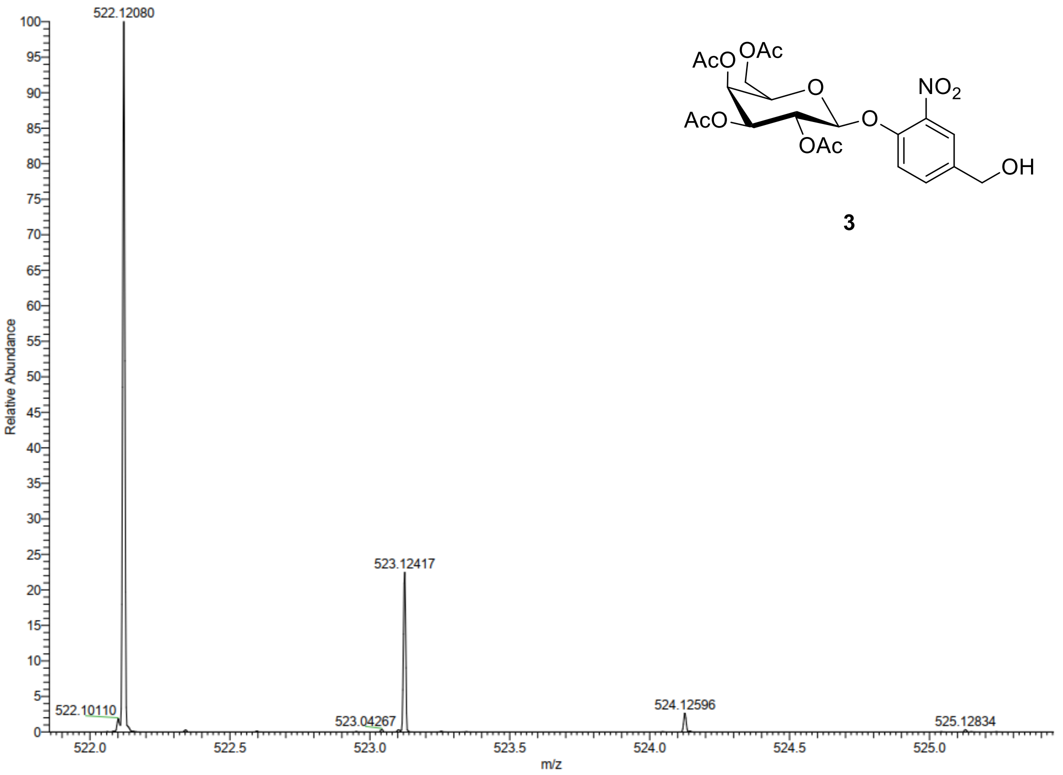

Figure S37. MALDI-HRMS spectrum of **3**.

MALDI-MS of (2*R*,3*S*,4*S*,5*R*,6*S*)-2-(acetoxymethyl)-6-(4-(((bis(diisopropylamino)phosphino)oxy)methyl)-2-nitrophenoxy)tetrahydro-*2H*-pyran-3,4,5-triyl triacetate (4)

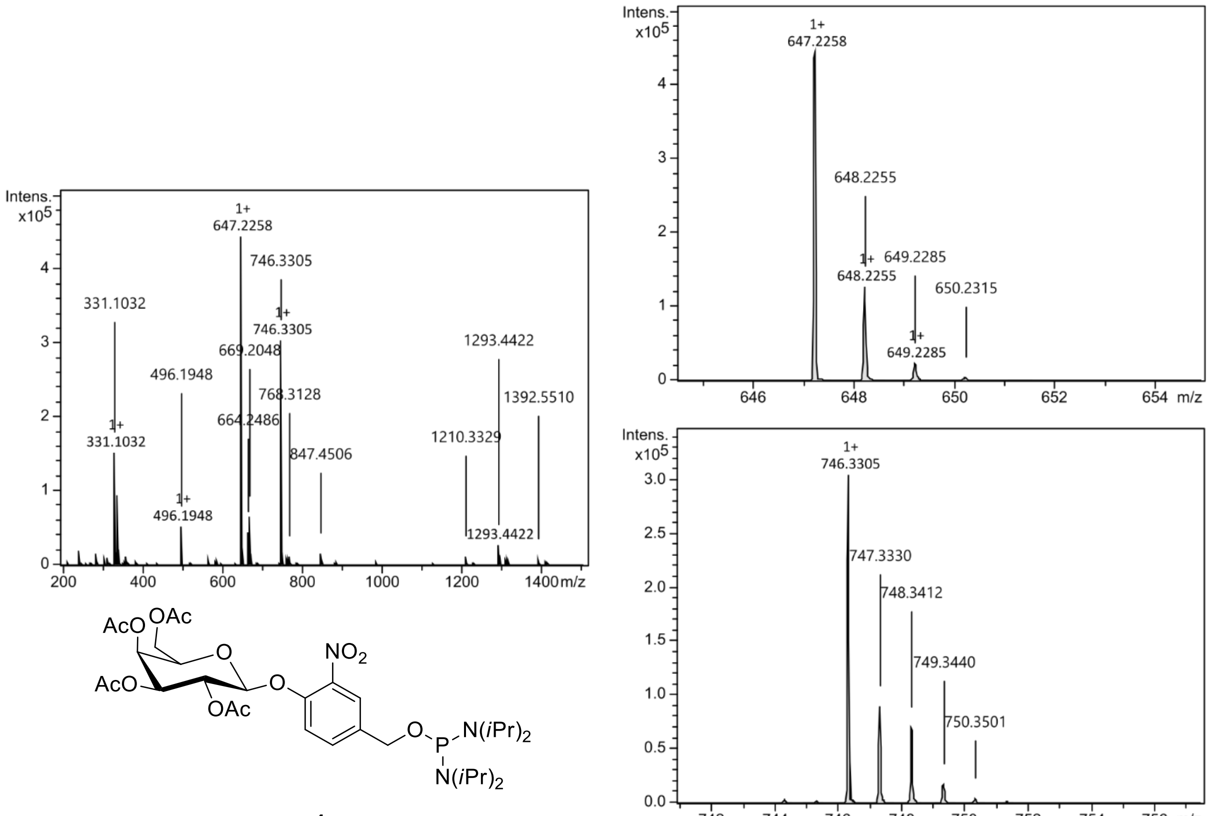

4

Figure S38. ESI-HRMS spectra of **4**.

MALDI-MS of (2*R*,3*S*,4*S*,5*R*,6*S*)-2-(acetoxymethyl)-6-(4-((((((2*R*,3*S*,5*R*)-2-((bis(4-methoxyphenyl) (phenyl)-methoxy)-methyl)-5-(5-methyl-2,4-dioxo-3,4-dihydropyrimidin-1(2*H*)-yl)tetrahydrofuran-3-yl)oxy) (diiso-propylamino)phosphino)-oxy)methyl)-2-nitrophenoxy)tetrahydro-2*H*-pyran-3,4,5-triyl triacetate (5)

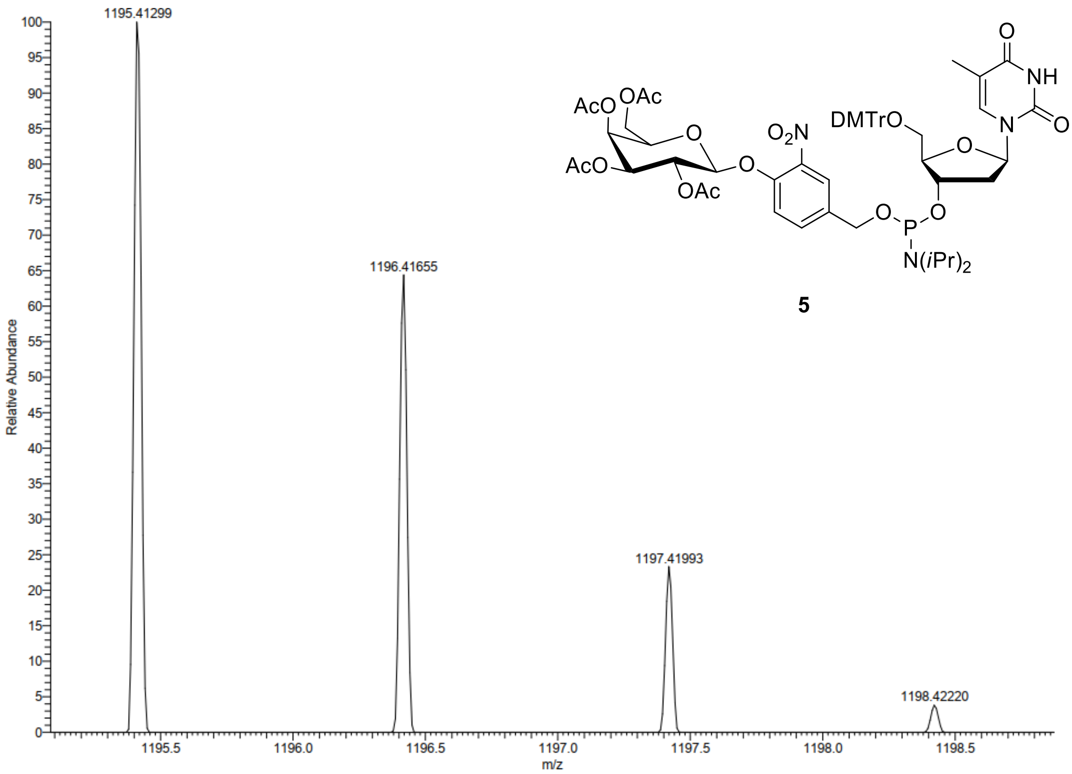

Figure S39. MALDI-HRMS spectrum of **5**.

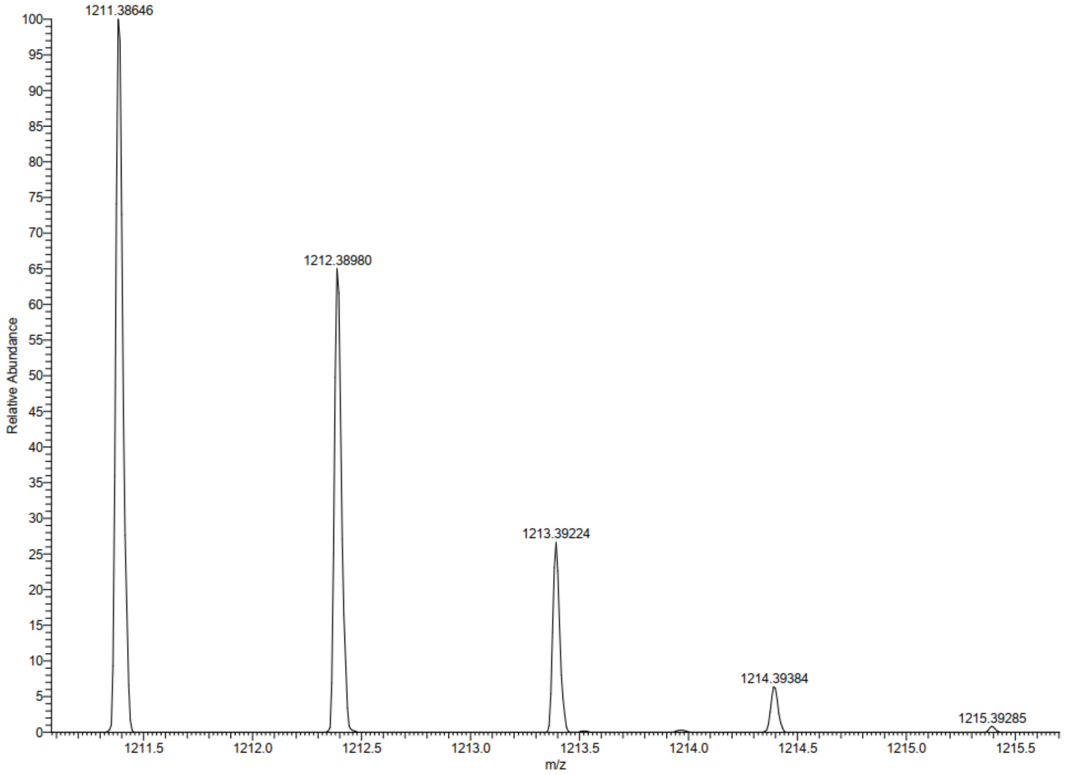

Figure S40. MALDI-HRMS spectrum of **5**.

MALDI-MS of (2*R*,3*S*,4*S*,5*R*,6*S*)-2-(acetoxymethyl)-6-(4-(((1-((2*R*,4*S*,5*R*)-4-((*tert*-butyldimethylsilyl)oxy)-5-(((*tert*-butyldimethylsilyl)oxy)methyl)tetrahydrofuran-2-yl)-5-methyl-2-oxo-1,2-dihydropyrimidin-4-yl)oxy)methyl)-2-nitrophenoxy)-tetrahydro-2*H*-pyran-3,4,5-triyl triacetate (6)

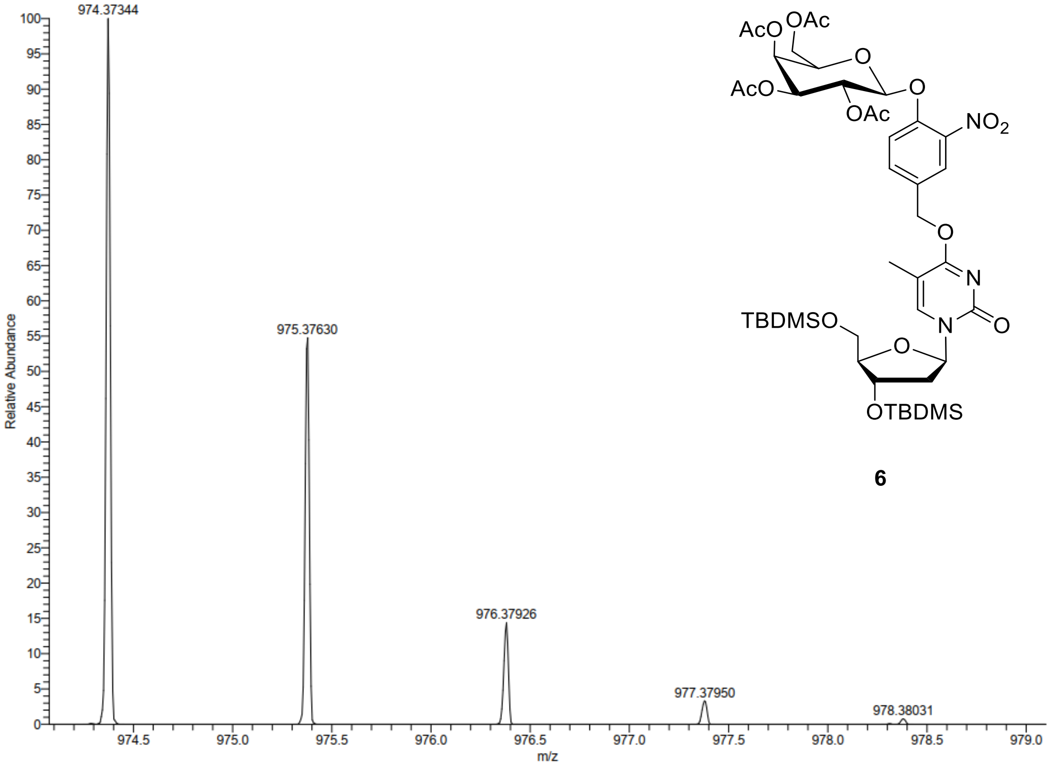

6

Figure S41. MALDI-HRMS spectrum of **6**.

MALDI-MS of (2*R*,3*S*,4*S*,5*R*,6*S*)-2-(acetoxymethyl)-6-(4-(((1-((2*R*,4*S*,5*R*)-4-hydroxy-5-(hydroxymethyl) tetrahydrofuran-2-yl)-5-methyl-2-oxo-1,2-dihydropyrimidin-4-yl)oxy)methyl)-2-nitrophenoxy)tetrahydro-2*H*-pyran-3,4,5-triyl triacetate (7)

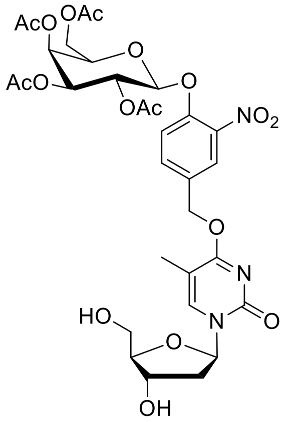

7

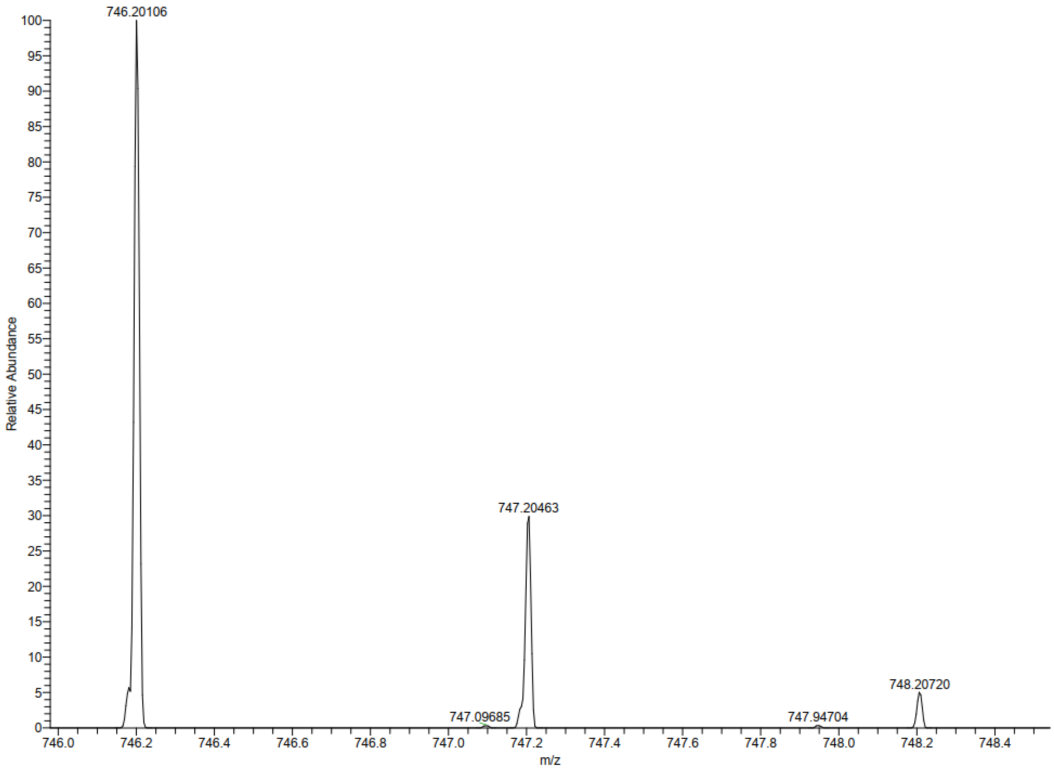

Figure S42. MALDI-HRMS spectrum of **7**.

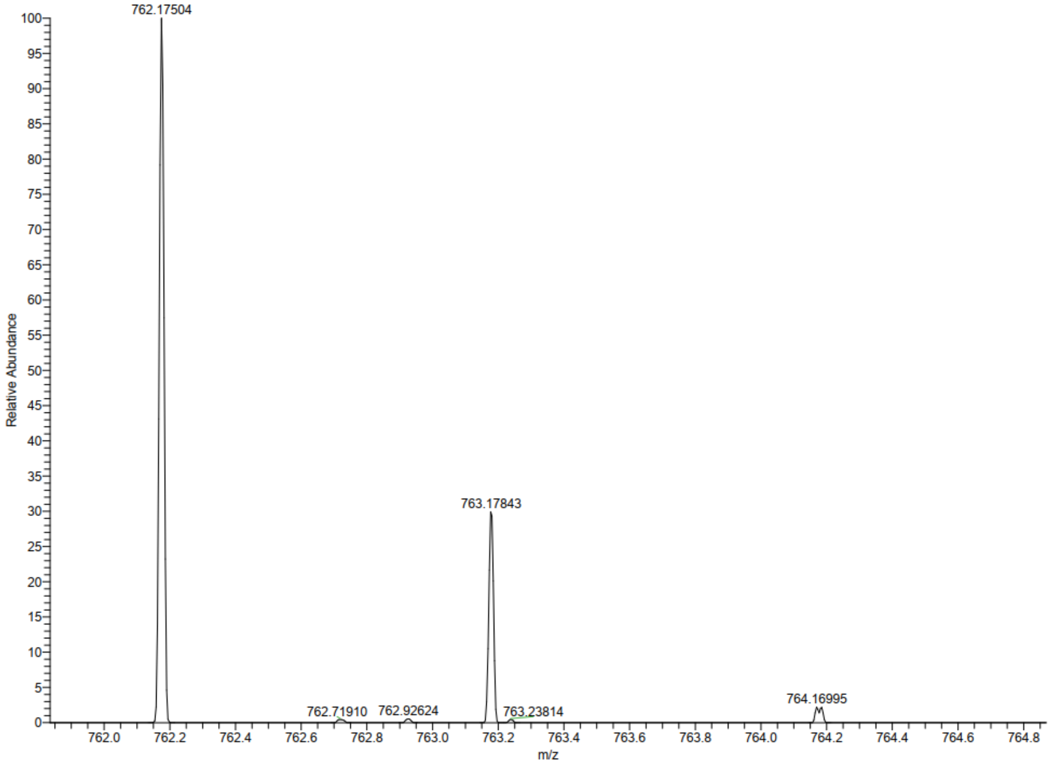

Figure S43. MALDI-HRMS spectrum of **7**.

MALDI-MS of (2*R*,3*S*,4*S*,5*R*,6*S*)-2-(acetoxymethyl)-6-(4-(((1-((2*R*,4*S*,5*R*)-5-((bis(4-methoxyphenyl)(phenyl) methoxy-methyl)-4-hydroxytetrahydrofuran-2-yl)-5-methyl-2-oxo-1,2-dihydropyrimidin-4-yl)oxy)methyl)-2-nitrophenoxy)tetra-hydro-2*H*-pyran-3,4,5-triyl triacetate (8)

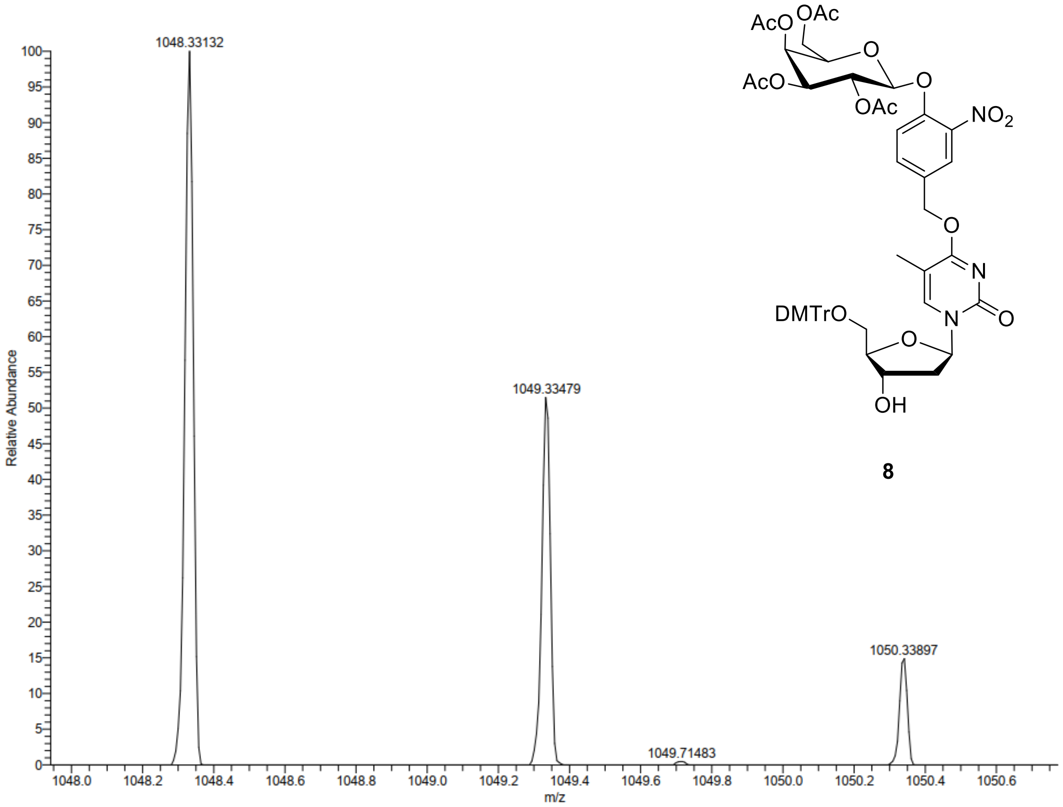

Figure S44. MALDI-HRMS spectrum of **8**.

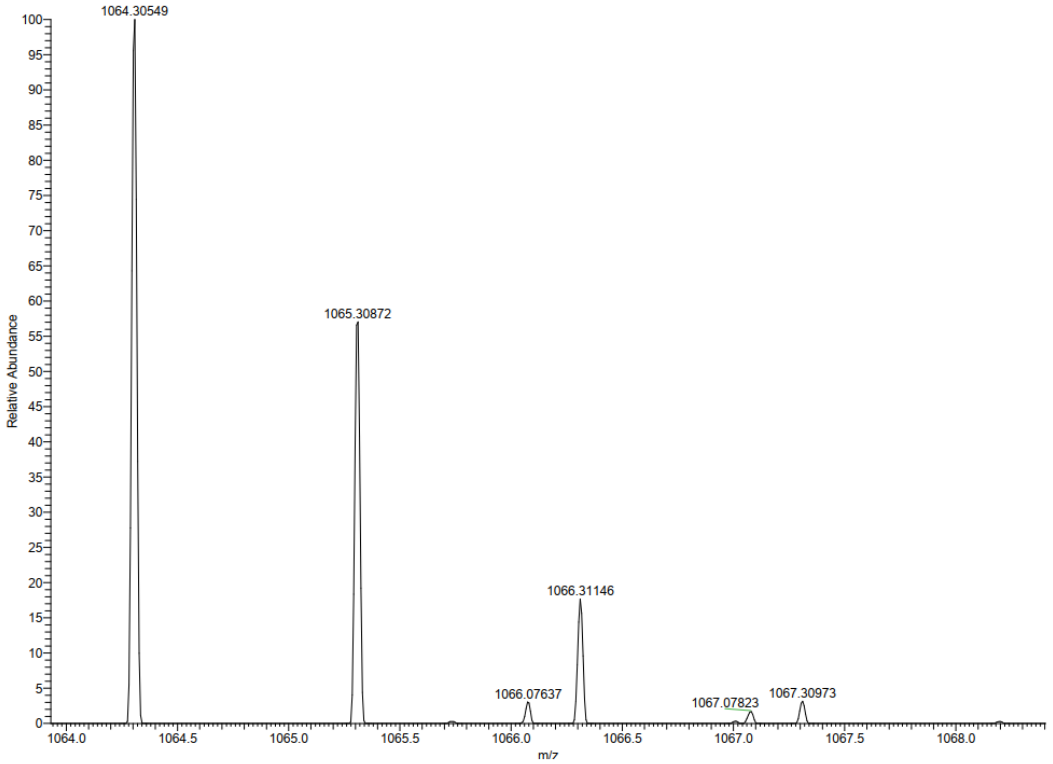

Figure S45. MALDI-HRMS spectrum of **8**.

MALDI-MS of (2*R*,3*S*,4*S*,5*R*,6*S*)-2-(acetoxymethyl)-6-(4-(((1-((2*R*,4*S*,5*R*)-5-((bis(4-methoxyphenyl) (phenyl) methoxy)-methyl)-4-(((2-cyanoethoxy)(diisopropylamino)phosphino)oxy)tetrahydrofuran-2-yl)-5-methyl-2-oxo-1,2-dihydro-pyrimidin4-yl)oxy)methyl)-2-nitrophenoxy)tetrahydro-2*H*-pyran-3,4,5-triyl triacetate (9)

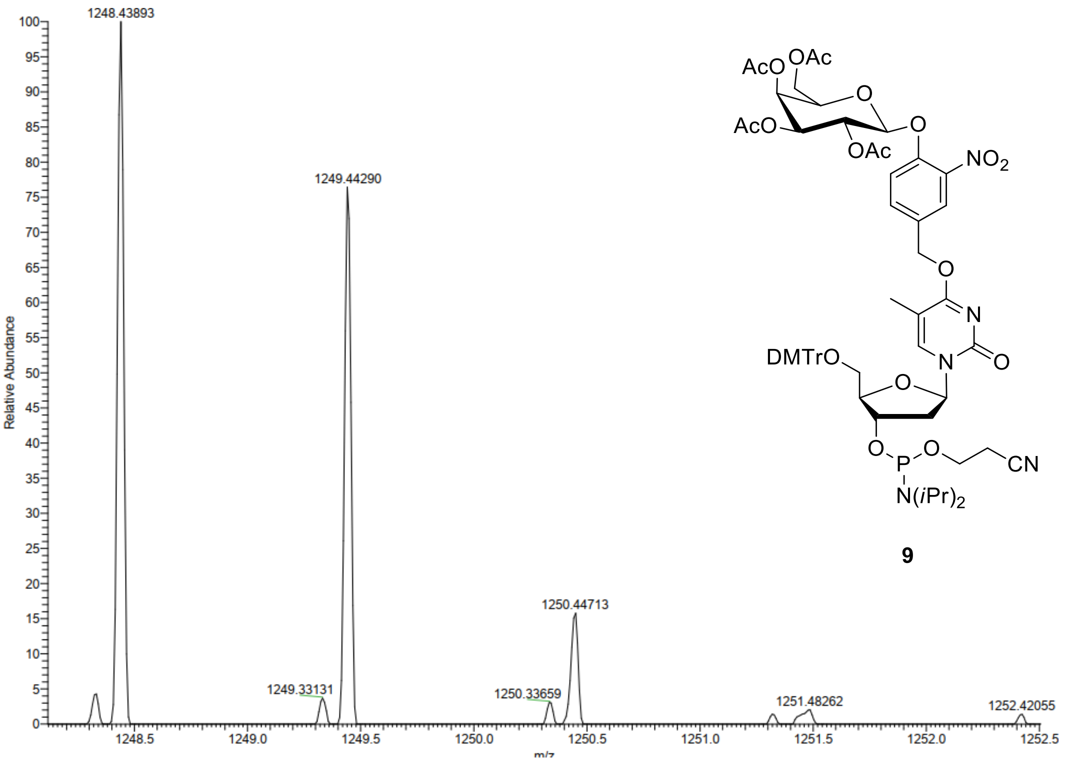

Figure S46. MALDI-HRMS spectrum of **9**.

ESI-MS of 4-(1-hydroxybut-3-yn-1-yl)-2-nitrophenol (10)

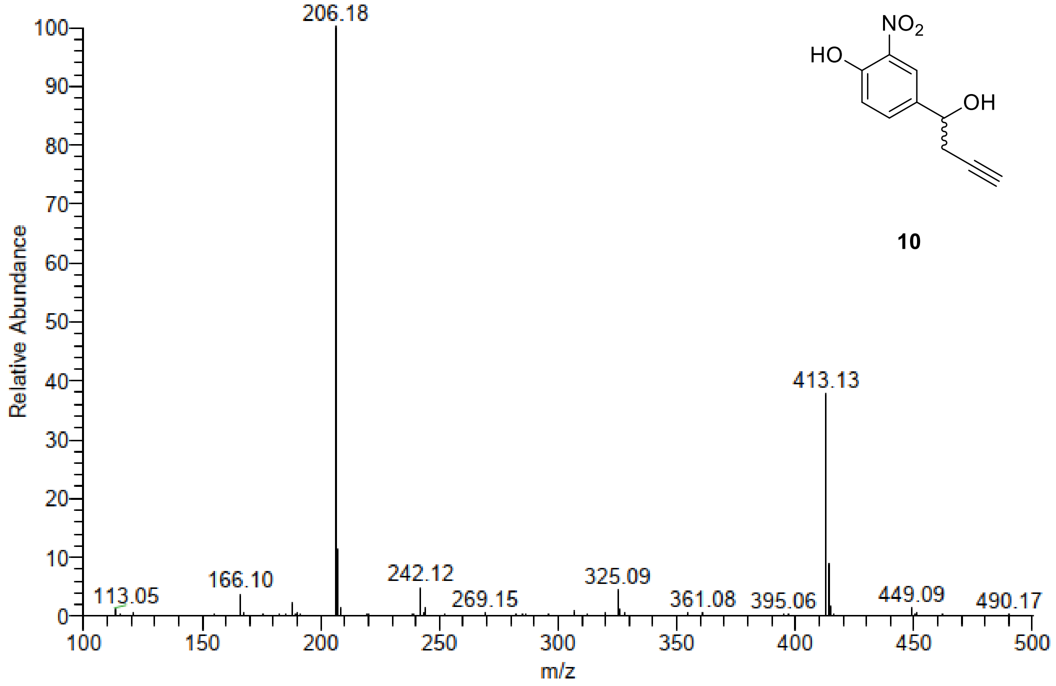

Figure S47. ESI-MS spectrum of **10**.

ESI-MS of (2*R*,3*S*,4*S*,5*R*,6*S*)-2-(acetoxymethyl)-6-(4-(1-hydroxybut-3-yn-1-yl)-2-nitrophenoxy)tetrahydro-2*H*-pyran-3,4,5-triyl triacetate (11)

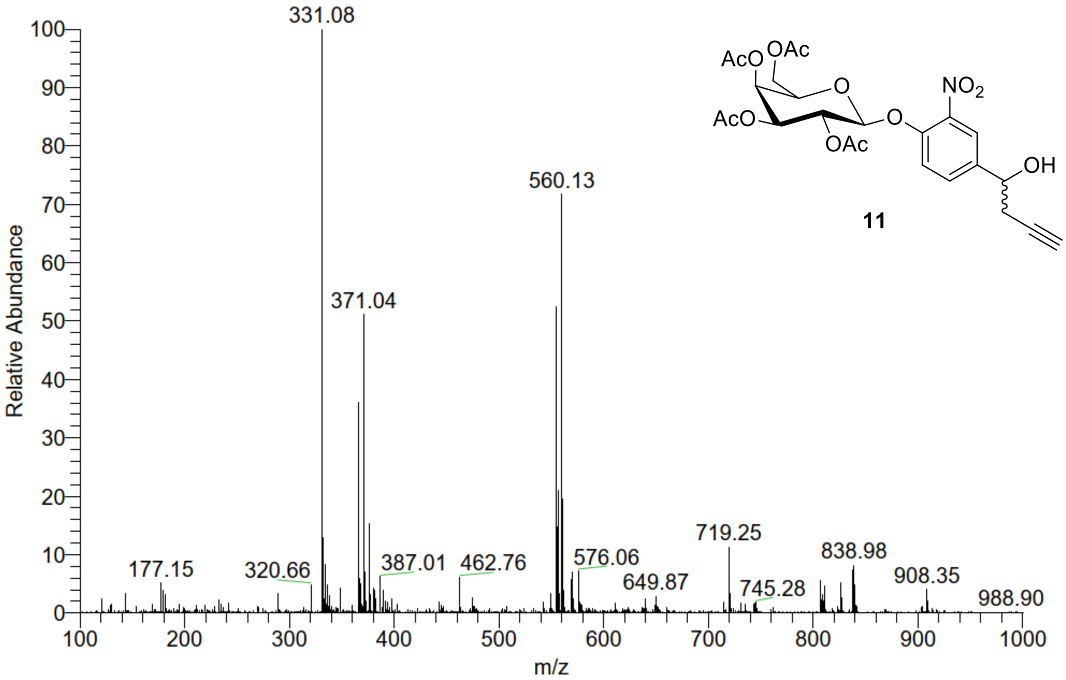

Figure S48. ESI-MS spectrum of **11**.

MALDI-MS of (2*R*,3*S*,4*S*,5*R*,6*S*)-2-(acetoxymethyl)-6-(4-(1-(((2-cyanoethoxy)(diisopropylamino)phosphino)oxy)but-3-yn-1-yl)-2-nitrophenoxy)tetrahydro-*2H*-pyran-3,4,5-triyl triacetate (12)

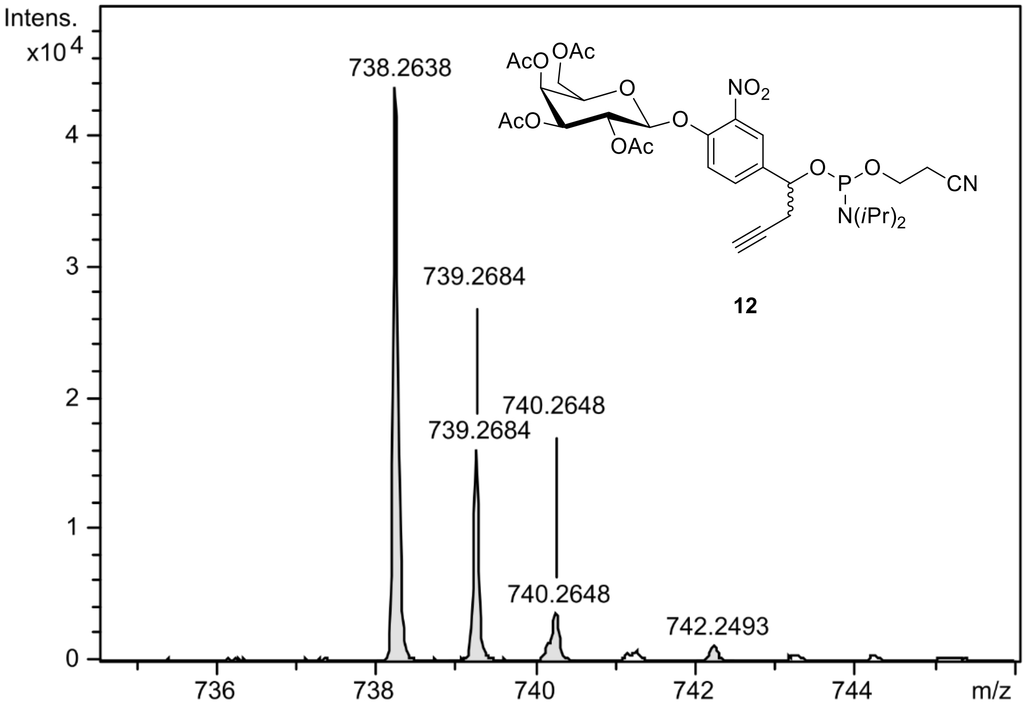

Figure S49. ESI-HRMS spectrum of **12**.

12. Mass Spectra (Oligonucleotides)

ON1 (5’-GCA TAA ATA AAG GTG-3’), calculated average mass: 4649.0663 Da

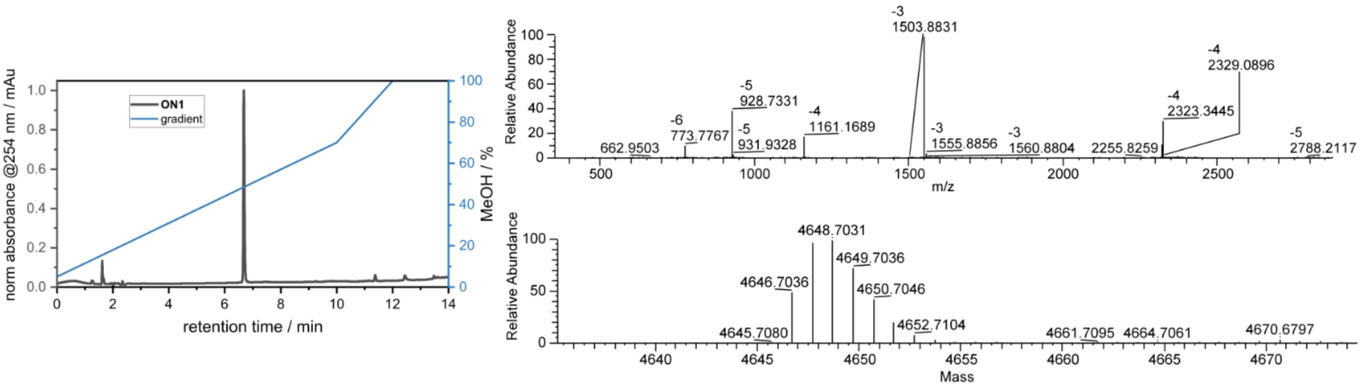

Figure S50. LC-MS spectra of **ON1**.

ON2 (5’-GCA TAA AT^{p}A AAG GTG-3’), calculated average mass: 4962.3264 Da

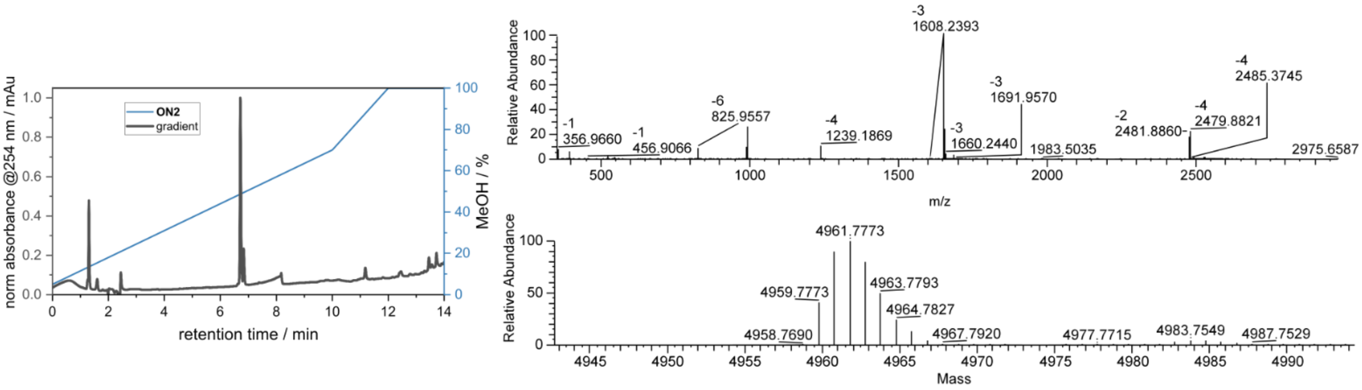

Figure S51. LC-MS spectra of **ON2.**

ON3 (5’-GCA TAA AT^{nb}A AAG GTG-3’), calculated average mass: 4962.3264 Da

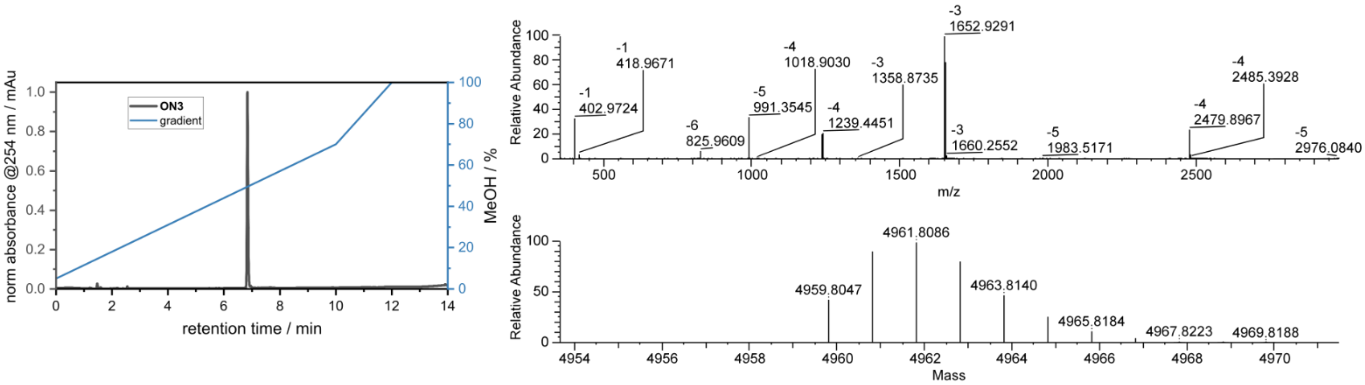

Figure S52. LC-MS spectra of **ON3**.

ON3-β-Gal (5'-GCA TAA ATA AAG GTG-3'), calculated average mass: 4649.0663 Da

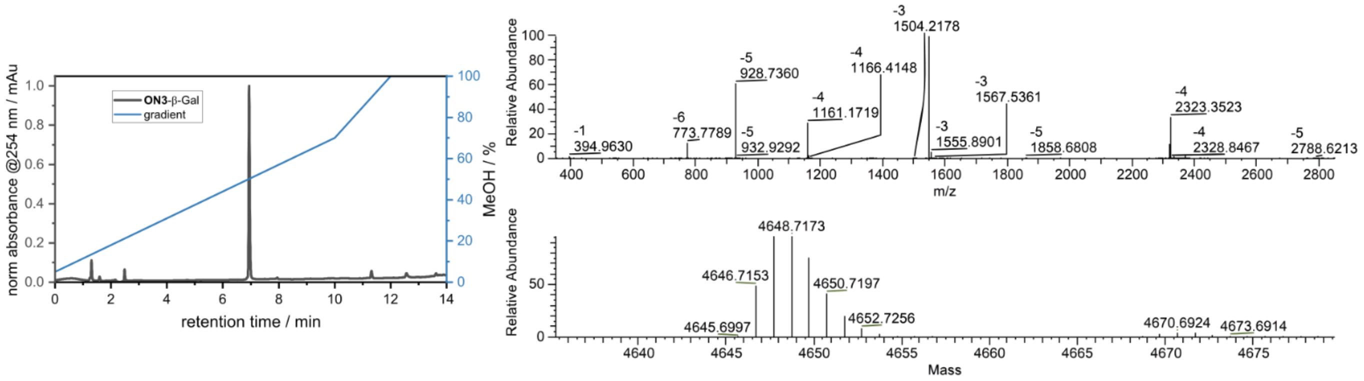

Figure S53. LC-MS spectra of **ON3**-β-Gal.

ON4 (5'$_{f}^{alkyne}$ GCA TAA ATA AAG GTG 3'^{alkyne}), calculated average mass: 5353.5765 Da

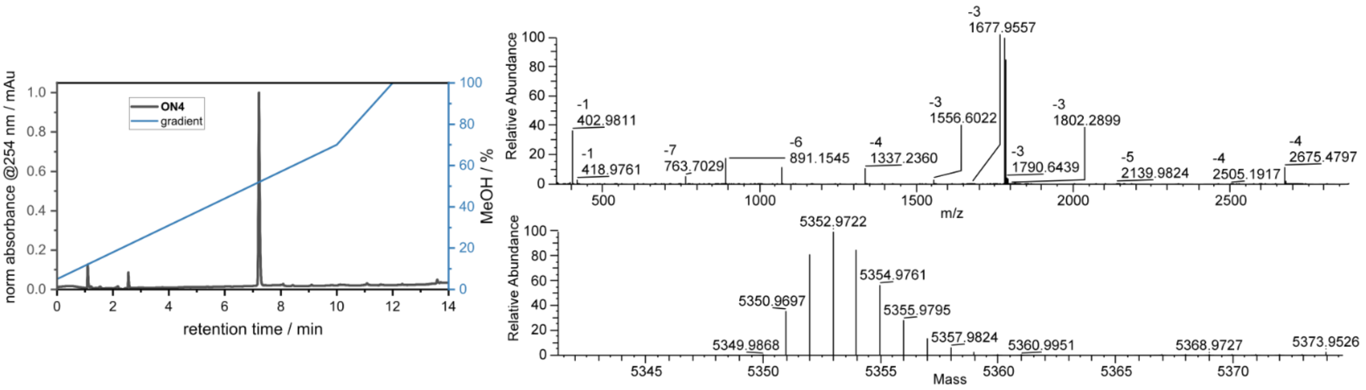

Figure S54. LC-MS spectra of **ON4**.

ON4-β-Gal (5'-PO_4- GCA TAA ATA AAG GTG 3'^{alkyne}), calculated average mass: 5002.2684 Da

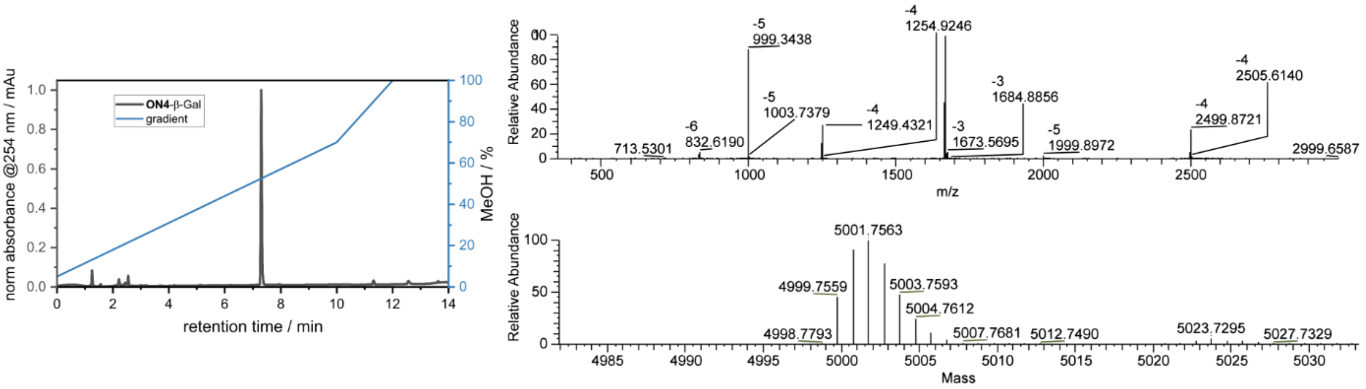

Figure S55. LC-MS spectra of **ON4-β-Gal**.

cON4 (5'$_{f}^{alkyne}$ GCA TAA ATA AAG GTG 3'^{alkyne} + AMB-linker), calculated average mass: 5541.7658 Da

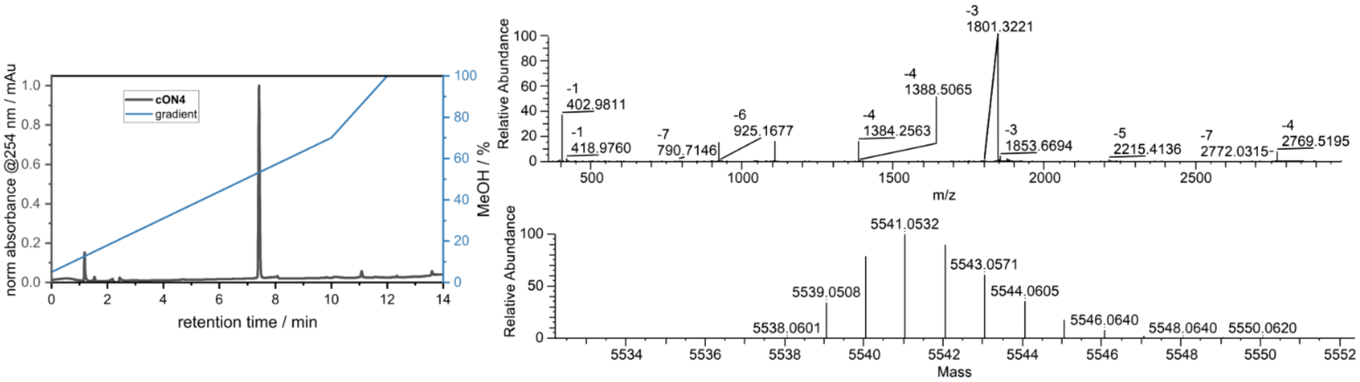

Figure S56. LC-MS spectra of **cON4**.

linON4 (5'$_{f}^{alkyne}$ GCA TAA ATA AAG GTG 3'^{alkyne}), calculated average mass: 5397.6405 Da

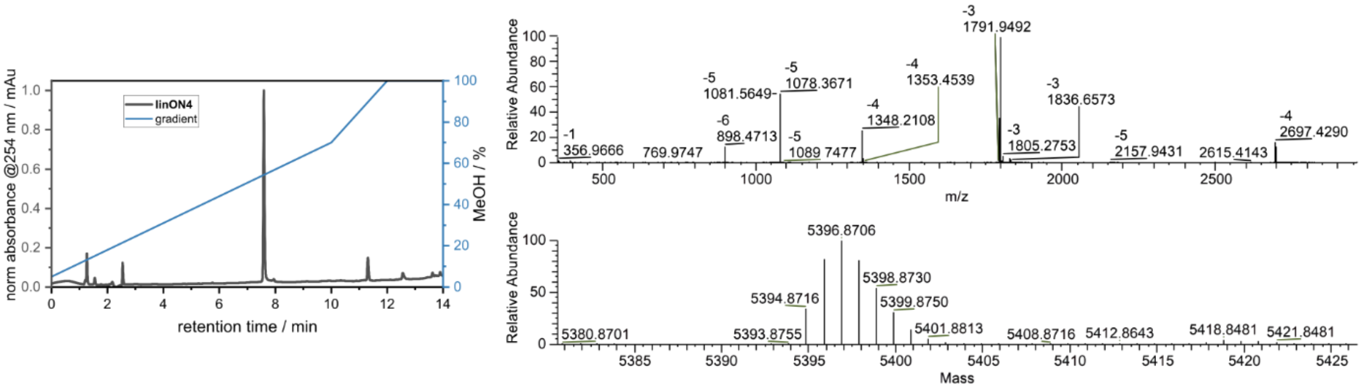

Figure S57. LC-MS spectra of **linON4**.

ON5 (5'-CAC CTT TAT TTA TGC-3'), calculated average mass: 4492.9406 Da

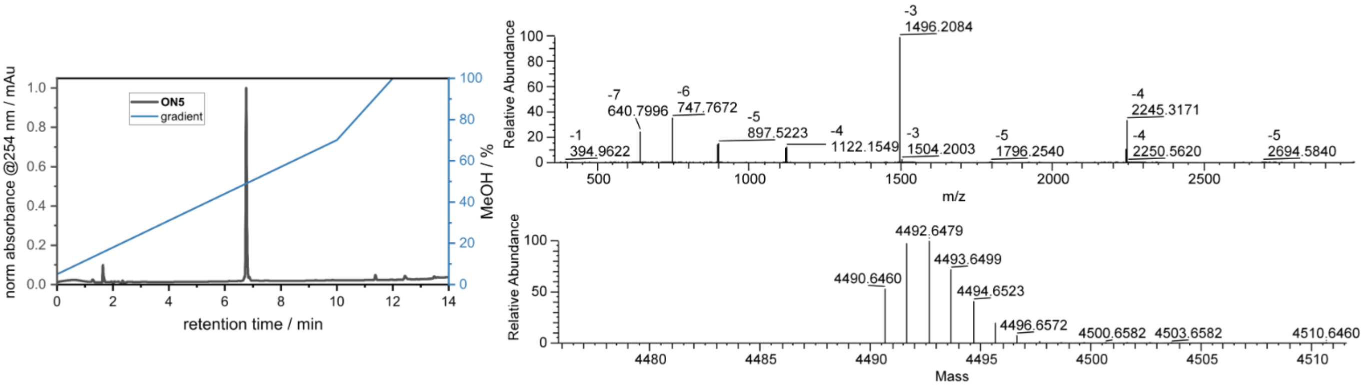

Figure S58. LC-MS spectra of **ON5**.

13. References

[1] S. Wingert, F. B. Thalheimer, N. Haetscher, M. Rehage, T. Schroeder, M. A. Rieger, *Stem Cells* **2016**, *34*, 699–710.
